# Synthesis and Conformational Analysis of Pyran Interhalide Analogues of Galactose, Mannose, Talose, and Glucose

**DOI:** 10.1002/chem.202501689

**Published:** 2025-07-14

**Authors:** Olivier Lessard, Mathilde Grosset‐Magagne, Pierangelo Metrangolo, Denis Giguère

**Affiliations:** ^1^ Département de Chimie 1045 av. De la Médecine Université Laval Québec City Quebec G1V 0A6 Canada; ^2^ Department of Chemistry, Materials, and Chemical Engineering “Giulio Natta” Politecnico di Milano Via L. Mancinelli 7 Milano 20131 Italy

**Keywords:** conformational analysis, halogen bonding, halogenated carbohydrates, pyran interhalides

## Abstract

There is growing interest in the preparation of multivicinal interhalide molecules. Herein, we described the synthesis of several pyran interhalide analogues of d‐galactose, d‐mannose, d‐talose, and d‐glucose. A robust and simple halogeno‐divergent strategy provided pyran‐bearing multivicinal interhalide stereocenters. However, a different strategy was implemented for the synthesis of glucose interhalides. The solid‐state conformational analysis of some interhalide analogues showed differences in the deviation of the intra‐annular torsion angles. Moreover, we observed the shortest F⋅⋅⋅I halogen bonding (XB) involving Csp^3^‐bound I and F atoms.

## Graphical abstract

The synthesis and conformational analysis of pyran interhalides unveiled the shortest F⋅⋅⋅I halogen bonding involving Csp^3^‐bound I and F atoms.

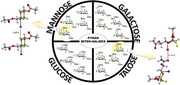

## 1 Introduction

Polyfluorinated carbohydrates have great biological potential, with many examples reported thus far.^[^ ^1^ ^]^ Pyran interhalide analogues of carbohydrates are scarcer, and only a few examples are reported as useful tools in glycobiology.^[^ ^2^ ^]^ The lack of efficient synthesis of sugar interhalides might explain the limited number of biological investigations of these interesting organohalogens. Only a handful of reports shows the study of synthetic^[^ ^3^ ^]^ and natural products^[^ ^4^ ^]^ having multivicinal interhalide stereocenters. Nevertheless, the incorporation of halogens into small molecules can improve cellular uptake and enhance membrane binding and permeation.^[^ ^5^ ^]^ Related to biological systems, the increase in binding affinity arises from the rich palette of noncovalent interactions given by halogen atoms, including halogen bonding (XB), that is, the interaction involving halogen atoms as electrophilic sites.^[^ ^6^ ^]^ XB was also successfully used for the activation of glycosyl halides.^[^ ^7^ ^]^

**Figure 1:**
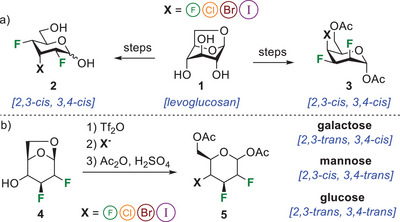
Synthesis of trihalogenated pyrans: a) Chiron approach to pyran interhalide analogues of allopyranoses **2** and talopyranoses **3**;^[^ ^9^, ^10^ ^]^ b) Synthetic strategy to multivicinal interhalide analogues of d‐galactose, d‐mannose, and d‐glucose (this work).

From our end, we developed synthetic routes to multivicinal organofluorines to unveil some of their unique properties, such as the lipophilicity, conformation, and biological activities.^[^ ^8^ ^]^ Moreover, we developed a halo‐divergent synthetic route of pyran interhalide analogues of d‐allose **2** from levoglucosan **1** (Figure 1a).^[^ ^9^ ^]^ Allopyranose interhalides **2** (incorporating the 2,3‐*cis*, 3,4‐*cis* relationship for the halogens) was the starting point to complex trihalo‐ and tetrahalo‐hexanetriols. Also, halogenated analogues of Pitolisant were prepared, highlighting an approach to increase the molecular dipole of piperidines. Recently, we reported the synthesis of pyran interhalide analogues of d‐talopyranose **3** that also integrates the 2,3‐*cis*, 3,4‐*cis* relationship for the halogens (Figure 1a).^[^ ^10^ ^]^ Talose interhalides **3** showed distinct solid‐state conformations because of 1,3‐diaxial repulsion between fluorine at C2 and halogen atoms. Additionally, we reported the first application of XB in the context of solid‐state ordering of pyran interhalides. Lastly, the group of Linclau reported the synthesis and lipophilicity evaluations of a series of dideoxygenated chloro‐fluorosugars.^[^ ^11^ ^]^ It was shown that the log *P* increases by an average of 1.37 log *P* units for the deoxychlorination products as compared to 0.83 log *P* for the corresponding deoxyfluorination products.

We wish to extend our approach to the synthesis of three sets of pyran interhalide analogues from 1,6‐anhydro‐2,3‐dideoxy‐2,3‐difluoro‐β‐d‐hexopyranoses **4** (Figure 1b). A robust and simple strategy involving a halogeno‐divergent route provided d‐galactose (2,3‐*trans*, 3,4‐*cis* relationship for the halogens), d‐mannose (2,3‐*cis*, 3,4‐*trans* relationship for the halogens), and d‐glucose (2,3‐*trans*, 3,4‐*trans* relationship for the halogens). The solid‐state conformational analysis and comparison of ^19^F resonances of some analogues add more data to the field of polyhalogenated molecules.

## 2 Results and discussions

**Scheme 1:**
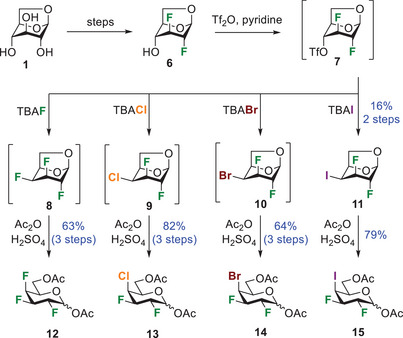
Synthesis of halogenated galactopyranose analogues **12 − 15** that integrate a 2,3‐*trans*, 3,4‐*cis* relationship for the halogens.

Our recent discovery that the nature and the stereochemistry of halogen atoms can have a strong impact on conformation and lipophilicity motivated us to investigate original pyran interhalides.^[^ ^9^, ^10^ ^]^ We first targeted galactopyranose interhalide analogues using an halo‐divergent synthetic route starting from known 1,6‐anhydro‐2,3‐dideoxy‐2,3‐difluoro‐β‐d‐glucopyranose **6**,^[^ ^8^, ^12^ ^]^ readily accessible from levoglucosan **1** (Scheme 1). Compound **6** was activated as a triflate, providing intermediate **7**, which was directly treated with a nucleophilic halogen source, providing the desired halogen substitution at C4 with inversion of configuration. Intermediates **8 − 10** were advanced to the next step without further purification, except for compound **11** because of the formation of side products after the iodination step. The halogenated **8 − 11** were treated under acetolysis conditions, providing galactopyranose analogues **12 − 15** in modest to good yields. The novel analogues integrate a 2,3‐*trans*, 3,4‐*cis* relationship for the halogens.

**Scheme 2:**
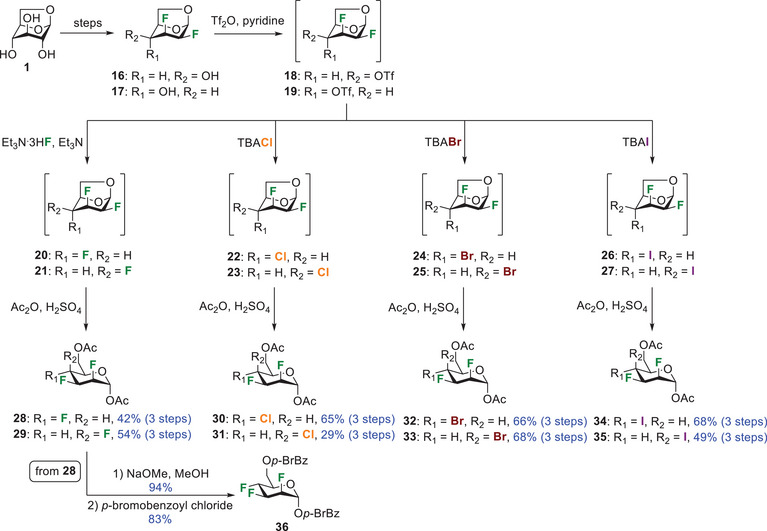
Synthesis of halogenated mannopyranose analogues **28**, **30**, **32**, and **34** and talopyranose analogues **29**, **31**, **33**, and **35** that integrate a 2,3‐*cis*, 3,4‐*trans* ^[^ ^10^ ^]^ and a 2,3‐*cis*, 3,4‐*cis* relationship for the halogens, respectively.

Next, we focused on the synthesis of mannopyranose interhalide analogues using the same strategy. As a point of comparison, we also report here the synthesis of talopyranose analogues.^[^ ^10^ ^]^ Starting from known difluorinated analogues **16** or **17**,^[^ ^8^ ^a]^ we were able to generate triflate intermediates **18** and **19** that were subjected to nucleophilic halogenation with inversion of configuration (Scheme 2). Halogenated mannopyranoses **20**, **22**, **24**, and **26**, along with talopyranoses **21**, **23**, **25**, and **27**, were treated under acetolysis conditions, affording halogenated pyran **28 − 35**. The installation of an equatorial chlorine and iodine proceeded in higher yield than the corresponding axial halogens (**30**: 65% vs. **31**: 29% and **34**: 68% vs. **35**: 49%). As for the bromine, the yields were similar for both isomers (**32**: 66% vs. **33**: 68%).

**Scheme 3:**
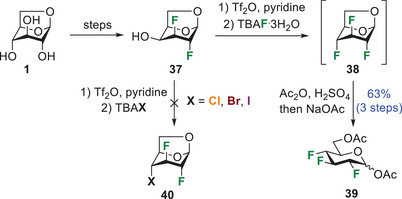
Synthesis of trifluorinated glucose analogue **39** and failed attempt to prepare glucopyranose interhalides **40**.

Our next task involved the preparation of halogenated glucose analogues using the same strategy as before. Thus, known 1,6‐anhydro‐2,3‐dideoxy‐2,3‐difluoro‐β‐d‐galactopyranose **37** ^[^ ^8^ ^a,^ ^12^ ^]^ was fluorinated at C4 using tetrabutylammonium fluoride (TBAF) via a triflate intermediate (Scheme 3). Acetolysis of **38** under acidic conditions provided trifluoroglucose analogue **39**. Despite many efforts, this strategy failed to produce interhalides **40**. In this instance, we observed formation of side products probably arising from an oxiranium‐like intermediate.^[^ ^8^ ^e]^ Consequently, we were compelled to use a different synthetic route to access pyran interhalide analogues of glucose.

**Scheme 4:**
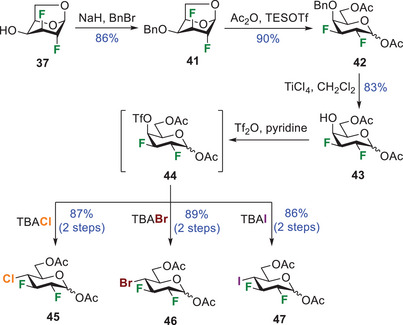
Synthesis of glucopyranose interhalide analogues **45 **–** 47** that integrate a 2,3‐*trans*, 3,4‐*trans* relationship for the halogens.

We rationalized that it would be easier to install a halogen equatorially on a pyran core via an S_N_2 reaction.^[^ ^13^ ^]^ Therefore, we cleaved the 1,6‐anhydro bridge, allowing the chair interconversion (^1^ *C*_4_ to ^4^ *C*_1_) prior to the nucleophilic halogenation steps. In this way, we first protected the alcohol **37** as benzyl ether in 86% yield (Scheme 4). Then, triethylsilyl triflate‐catalyzed acetolysis provided the desired diacetylated **42** in 90% yield, and a titanium tetrachloride‐mediated benzyl deprotection gave the corresponding free hydroxyl group at C4.^[^ ^14^ ^]^ We used the same nucleophilic substitution strategy as before. Thus, activation of the hydroxyl group **43** as triflate yielded intermediate **44** that was directly subjected to tetrabutylammonium chloride, bromide, or iodide, providing compounds **45**, **46**, and **47**, respectively. It is worth noting that in this case, the deoxyhalogenations proceeded in high yields over 2 steps (86 – 89%).

**Figure 2:**
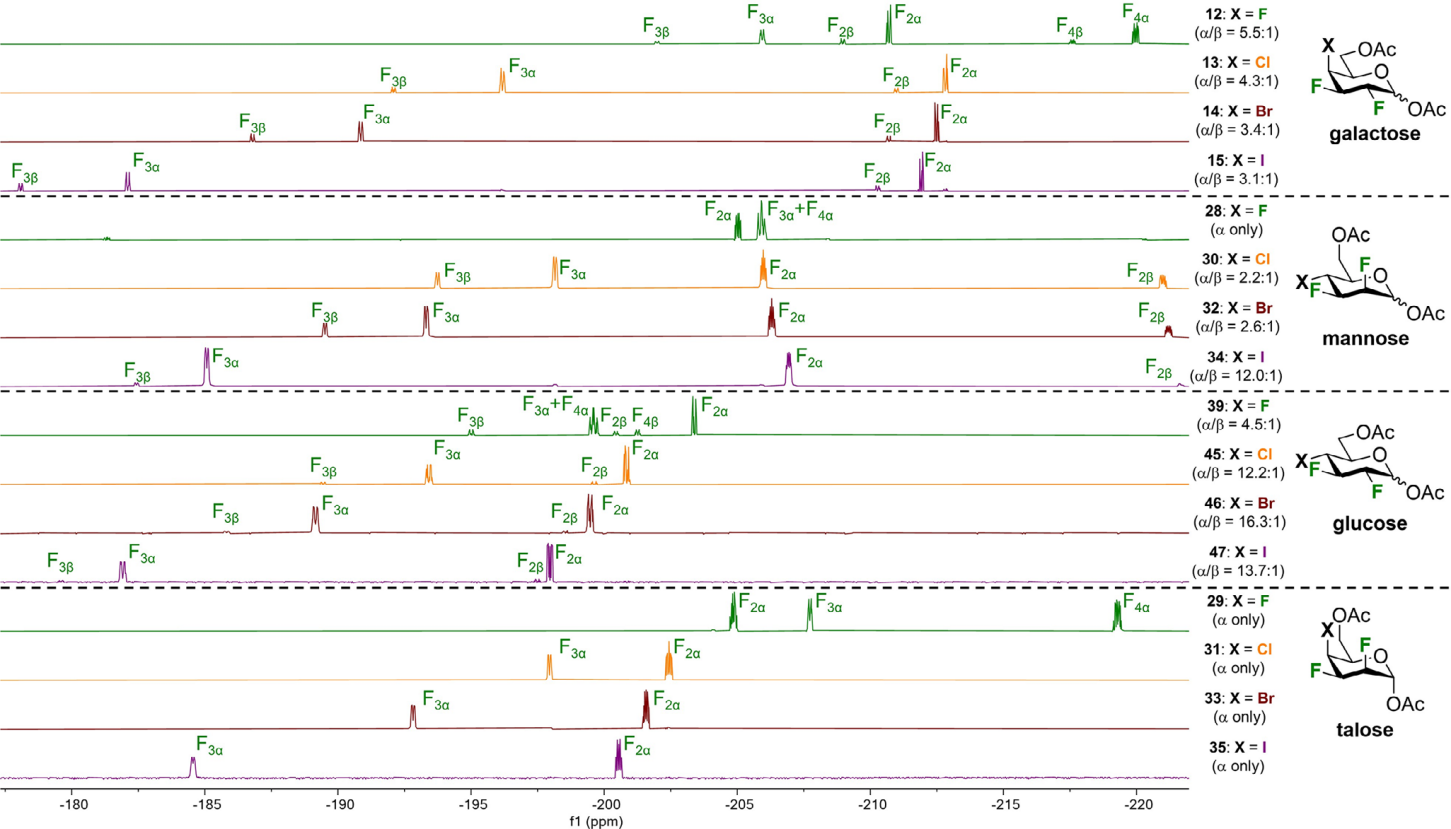
Direct comparison of ^19^F resonances (^19^F NMR; 470 MHz, CDCl_3_) of halogenated galactose analogues **12 **− **15**, mannose analogues **28**, **30**, **32**, and **34**, glucose analogues **39**, **45 **− **47**, and talose analogues **29**, **31**, **33**, and **35**.

With a set of pyran interhalide analogues of galactose, mannose, and glucose, we proceeded to compare their ^19^F resonance (^19^F NMR; 470 MHz, CDCl_3_) with talose interhalides (Figure 2).^[^ ^10^ ^]^ Firstly, all analogues adopt standard ^4^ *C*_1_‐like conformations. The ^19^F resonance of F3 expectedly occurs at a lower field than F2 for every trihalogenated pyran analogue. In the case of the glucose analogues, both ^19^F signals shift toward lower fields as the size of the halogen atom at the C4 position increases, from −203.39 to −197.96 ppm for F2 and from −199.69 to −181.94 ppm for F3. The same trend is observed for the talose analogue with shifts increasing from − 205.46 to **− **200.55 ppm for F2 and from **− **208.33 to **− **184.56 ppm for F3. In both series, the shift variation is more important for the F3 signal than the F2 signal. A similar trend is observed for the galactose analogues, except for the F2 signal of trifluorinated **12**, which is at a higher shift than the other analogues. The mannose series follows the general trend for the F3 signals, but the F2 signals surprisingly shift upfield with the increasing size of the halogen at the C4 position. The downfield shift of the F2 and F3 signals in the same series could be explained by steric deshielding.^[^ ^15^ ^]^ Due to the overlap of the van der Waals radii, it is hypothesized that the van der Waals forces of the halogen at the C4 position could restrict the electron motions of the fluorine atoms and therefore artificially lower their electron density. Lastly, the supporting information (Figure S1−S3) reports the ^19^F NMR predictions of acetylated pyran interhalides. Some NMR prediction tool don't consider the nature of the halogen at C4 for correctly predicting the chemical shift of fluorine atoms. For example, one of the biggest differences is the F4 signal of trifluorinated talose **29** (Δδ = −45.98 ppm). Consequently, the NMR predictions of such compounds remain very challenging, and this study adds more data to the field of NMR spectroscopy.

**Figure 3:**
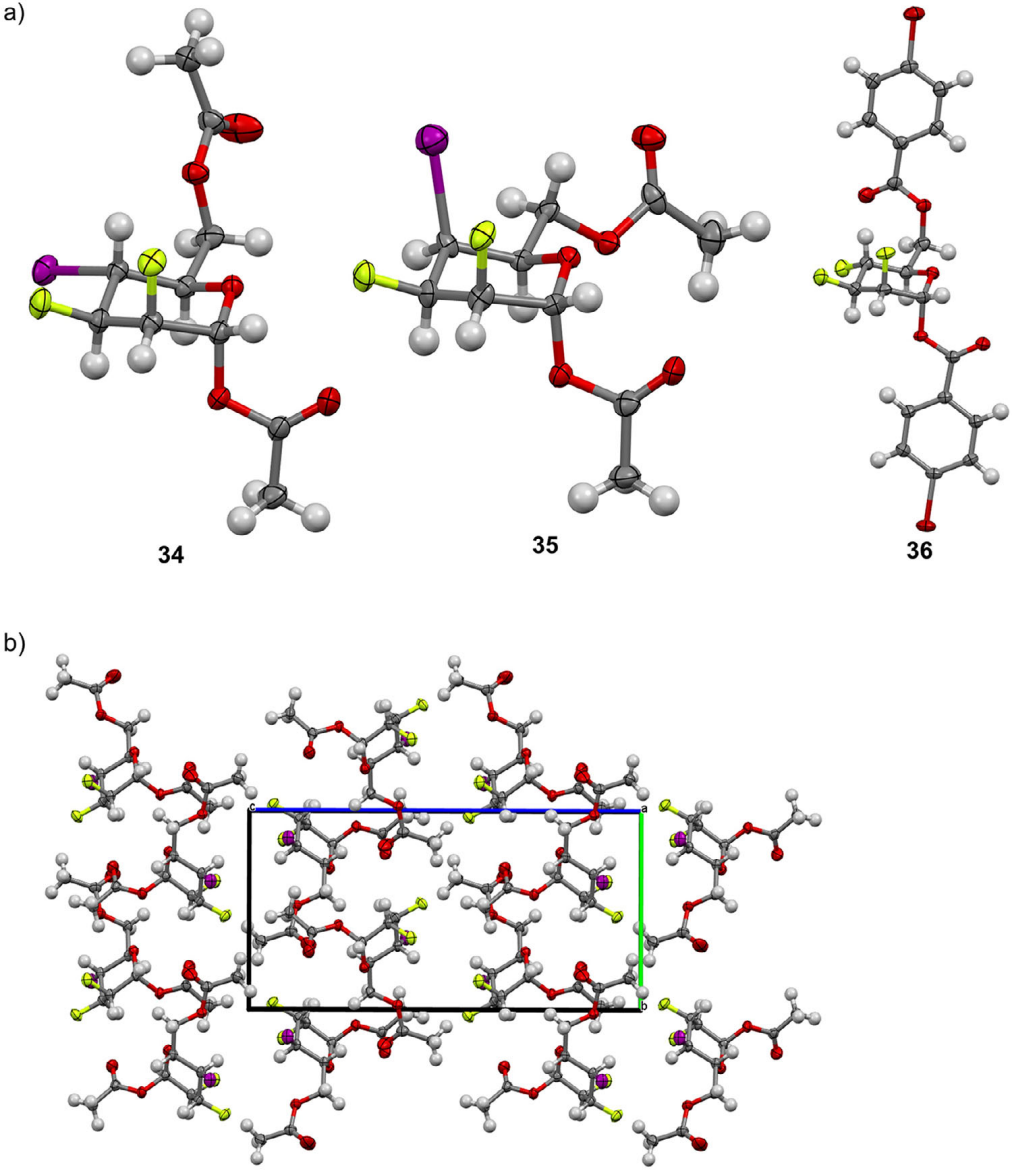
a) X‐ray analysis of compound **34 − 36**; b) extended packing arrangement (view along the *a*‐axis) of compound **34**. Oak Ridge Thermal‐Ellipsoid Plot (ORTEP) diagram showing 50% thermal ellipsoid probability: carbon (gray), oxygen (light red), fluorine (green), bromine (dark red), iodine (purple), and hydrogen (white).

The absolute configuration of iodides **34** ^[^ ^16^ ^]^ and **35** ^[^ ^17^ ^]^ and compound **36** ^[^ ^18^ ^]^ were unambiguously confirmed by single‐crystal diffraction analysis. Trifluorinated analogue **28** was not crystalline; therefore we were compelled to remove the acetyl protecting groups and generate the corresponding *p*‐bromobenzoate derivative **36** to obtain suitable crystalline material, as previously described (Scheme 2).^[^ ^8^ ^a]^ Our interest in the conformation of organohalogens motivated us to compare the solid‐state conformation of halogenated pyrans **34** and **36** ^8a^ with talose interhalide **35** (Figure 3a).^[^ ^10^ ^]^ Compounds **34** and **35** only differ by the stereochemistry of the iodine at C4. Similarly to its analogue **36**, compounds **34** and **35** adopt a standard ^4^ *C*_1_‐like conformation in the solid state. However, as for the C5 − C6 rotamer, compound **35** exhibits a *gt* conformation as opposed to a *gg* conformation for analogues **34** and **36**. For standard ^4^ *C*_1_‐like conformation, an equatorial substituent at C4 generally leads to a *gg* or a *gt* conformation, with the *tg* orientation forbidden.^[^ ^19^ ^]^ Figure 3b shows the extended packing arrangement of mannopyranose interhalide analogues **34**.

We recently reported that there is a strong deviation in the intra‐annular torsion angles for compound **35** arising from the repulsion of the axial fluorine at C2 and the axial iodine at C4.^[^ ^10^ ^]^ Compounds **34** and **36** have an equatorial halogen at C4; thus there is a reduced 1,3‐diaxial repulsion with C2─F, leading to a limited deviation from parallel alignment as shown in Table 1. The Newman projection of halogenated analogues shows deviations from parallel alignment for the C2─F and C4 substituents of 2.11° for **34** (R_1_ = H), 6.15° for **36** (R_1_ = H), and 18.59° for **35** (R_1_ = I). Thus, the stereochemistry of one halogen (the C4 iodine) can account for a difference in the deviation in the intra‐annular torsion angles of 16.48° (**34** vs. **35**). The greater deviation of **36** as compared to **34** is possibly due to the large *p*‐bromobenzoyl group, resulting in a *gg* conformation. As a comparison with natural carbohydrates, the deviations are 3.50° and 7.12° for the *gt* and the *gg* rotamers of native α‐d‐mannopyranose, respectively.^[^ ^20^ ^]^ For native α‐d‐talopyranose, the deviation is 5.92°.^[^ ^21^ ^]^

**Table 1:** 1,3‐Diaxial repulsion between CF2 and CH4 or CX4 bonds for compound **34 − 36**.^[^ ^a^ ^]^

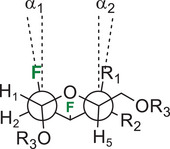				
Entry	Compounds	α_1_ (°)	α_2_ (°)	α_1_ + α_2_ (°)
1	**34** (R_1_ = H, R_2_ = I, R_3_ = Ac)	1.30	0.81	2.11
2	**35** (R_1_ = I, R_2_ = H, R_3_ = Ac)	7.06	11.53	18.59
3	**36** (R_1_ = H, R_2_ = F, R_3_ = *p*‐BrBz)	1.00	5.15	6.15

^[a]^ For α‐talopyranose: α_1_ = 5.58°, α_2_ = 0.34°, and α_1_ + α_2_ = 5.92°;^[^ ^21^ ^]^ for α‐mannopyranose (*gt* rotamer): α_1_ = 2.61°, α_2_ = 0.89°, and α_1_ + α_2_ = 3.50°; for α‐mannopyranose (*gg* rotamer): α_1_ = 2.20°, α_2_ = 4.92°, and α_1_ + α_2_ = 7.12°.^[^ ^20^ ^]^

We also assessed the Cremer–Pople ring puckering parameters (Table 2).^[^ ^22^ ^]^ These parameters take the form of a spherical polar set, *Q*, *θ*, and *φ*, for pyranoid rings.^[^ ^23^ ^]^ A smaller puckering amplitude (*Q*) values indicate a flattened ring. For an ideal cyclohexane chair, the puckering amplitude is 0.63 Å.^[^ ^22^ ^]^ The azimuthal angle (*θ*) represents the distortion of pyranose rings (*θ* = 0° is a perfect ^4^ *C*_1_ chair, and *θ* = 180° represents the ^1^ *C*_4_ chair). Expectedly, the distortion of the chair conformation of compound **35** is more important than compound **34**. In addition, the nature of the distortion is indicated by the meridian angle (*φ*). The distortion of the iodinated mannopyranose **34** is in a direction toward a ^4^ *H*_5_ conformation (*φ* ≈ 270°). The trifluorinated mannopyranose analogue **36** is distorted toward a ^2^ *H*_3_ conformation (*φ* ≈ 150°), and the iodinated talopyranose analogue **35** is distorted toward an *E*_5_ conformation (*φ* ≈ 300°). Once more, this set of data exemplifies the important conformational difference exerted by the stereochemistry of one iodine atom at C4 (**34** vs. **35**).

**Table 2:** Cremer–Pople ring puckering amplitudes (*Q*), theta (*θ*), and phi (*φ*) parameters.

	34	35	36
*Q* (Å)	0.559	0.523	0.529
*θ* (°)	5.235	7.248	1.609
*φ* (°)	263.056	307.711	149.255

**Figure 4:**
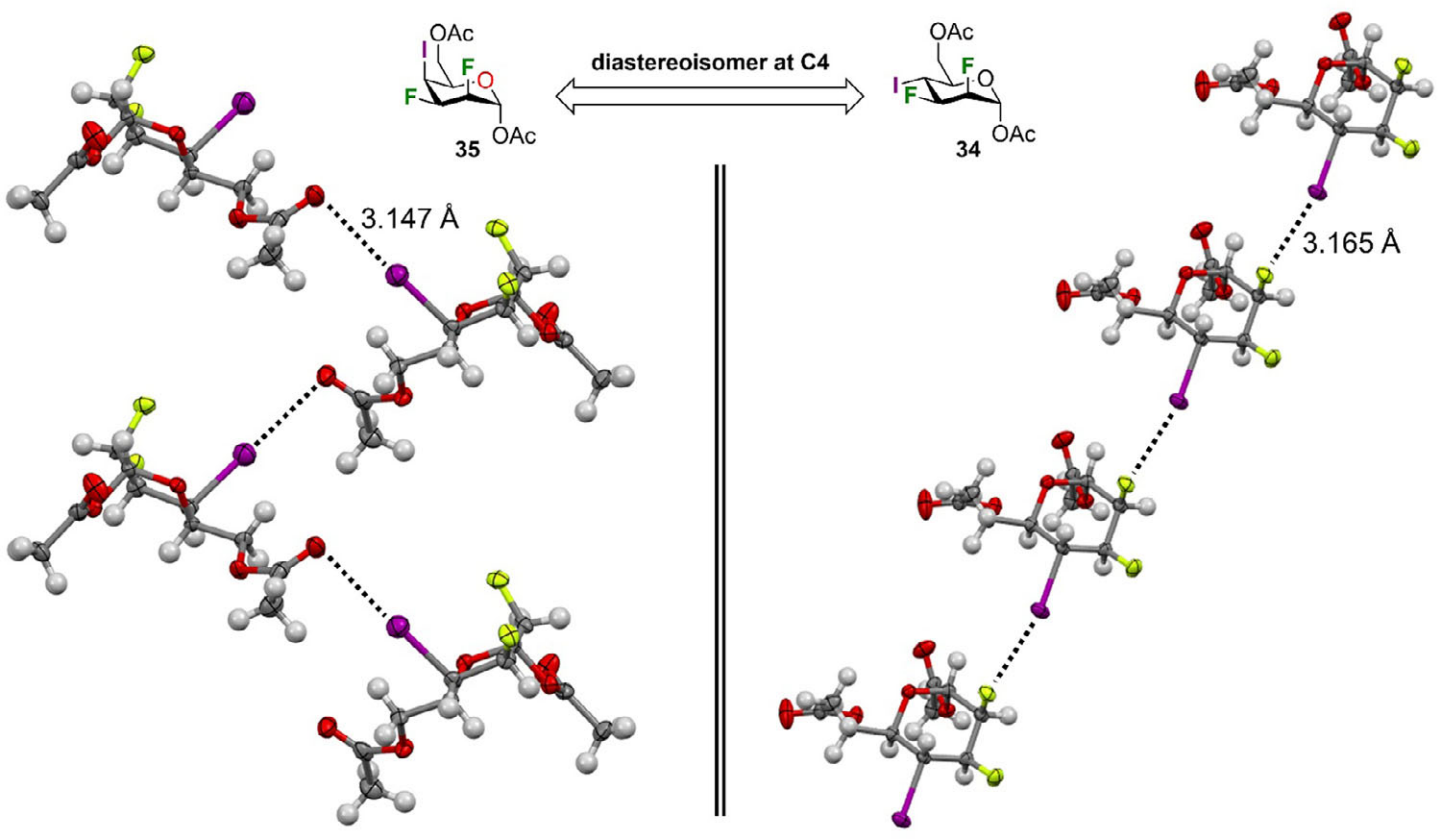
Packing arrangement of compound **34** and **35** showing XB interactions. ORTEP diagram showing 50% thermal ellipsoid probability: carbon (gray), oxygen (light red), fluorine (green), iodine (purple), and hydrogen (white).

Some of us recently reported the first example of XB stabilizing the crystal lattice of pyran interhalides.^[^ ^10^ ^]^ In particular, the iodinated talose interhalide **35** shows a very directional I⋅⋅⋅O contact involving the carbonyl oxygen atom of the acetate at C6 (IC⋅⋅⋅O distance = 3.147 Å; C‐I⋅⋅⋅O angle = 179.70°) of a nearby molecule (Figure 4). The observed distance roughly corresponds to a 10% reduction of the sum of the van der Waals radii for oxygen and iodine, that is, 1.52 and 1.98 Å,^[^ ^24^ ^]^ respectively, which is coherent with a medium‐strength XB. Similarly to other systems involving carbonyl oxygens, the iodine approach occurs in an out‐of‐plane mode (C = O⋅⋅⋅I angle = 104.23°).^[^ ^25^ ^]^ Interestingly, mannose interhalide **34**, instead, showed a completely different XB pattern. In particular, the C = O⋅⋅⋅I XB observed in **35** is replaced by a C‐F⋅⋅⋅I XB involving iodine and the fluorine atom at C3 (Figure 4). The F⋅⋅⋅I distance is 3.165 Å, which roughly corresponds to an 8% reduction of the sum of vdW radii for fluorine and iodine, that is, 1.45 and 1.98 Å,^[^ ^24^ ^]^ respectively, and the C‐I⋅⋅⋅F angle is 167.17°. The observed F⋅⋅⋅I XB is remarkably short, and, to the best of our knowledge, it is the shortest of its type, that is, involving Csp^3^‐bound I and F atoms, reported in the Cambridge Structural Database (CSD version 5.46, Update 1‐Feb 2025, 1 341 400 entries).^[^ ^26^ ^]^ Only FUMFUS shows a similar XB, although less directional.^[^ ^27^ ^]^ Whether or not organic fluorine may act as a hydrogen‐bond acceptor has been a controversial issue for more than four decades.^[^ ^28^ ^]^ The results reported in this article fuel the debate, clearly demonstrating that F⋅⋅⋅I interactions stabilize the crystal packing of mannose interhalides.

## 3 Conclusions

We described the synthesis of 4 halogenated pyran analogues of d‐galactose, d‐mannose, d‐talose, and d‐glucose. The synthetic strategy proceeded according to plan for all analogues except for glucopyranose interhalides. In this case, cleavage of the 1,6‐anhydro bridge preceded the deoxyhalogenation step in order to avoid formation of by‐products. Direct comparison of ^19^F resonances of all halogenated analogues showed interesting trends for the chemical shifts. All analogues adopt standard ^4^ *C*_1_‐like conformations, and the ^19^F resonance of F3 occurs at a lower field than F2 for all pyran interhalide analogues. Unexpectedly, the F2 signals of mannose shift upfield with the increasing size of the halogen at the C4 position. In the solid state, mannose interhalide **34** and talose interhalide **35** showed important conformational differences exerted by the stereochemistry of the iodine atom at C4. The Newman projections of halogenated analogues showed difference in the deviation in the intra‐annular torsion angles of 16.48°, and the Cremer–Pople ring puckering parameters showed differences in the distortion of the chair conformations. Furthermore, interhalides **34** and **35** show very different halogen bonding patterns stabilizing their crystal structures. In particular, the mannose derivative shows an unprecedented F⋅⋅⋅I halogen bonding, the shortest F⋅⋅⋅I halogen bonding involving Csp^3^‐bound I and F atoms. Finally, these findings shed more light on the intriguing nature of pyran interhalides for application in drug discovery, materials science, and other fields.

## Conflict of Interest

The authors declare no conflict of interest.

## Supporting information



Supporting Information

## Data Availability

The data that support the findings of this study are available in the supplementary material of this article.
