# Supplementary material for: Synthesis and Conformational Analysis of Pyran Interhalide Analogues of Galactose, Mannose, Talose, and Glucose
Source: Chemistry. 2025 Jul 14;31(43):e202501689. doi: 10.1002/chem.202501689 (PMC12319363; doi:10.1002/chem.202501689)
Supplement: Supplementary file 1 — Supporting Information [file CHEM-31-e202501689-s001.pdf]

Electronic Supporting Information

Synthesis and Conformational analysis of Pyran Inter-Halide Analogues of Galactose, Mannose, Talose, and Glucose

Olivier Lessard,[a] Mathilde Grosset-Magagne,[a] Pierangelo Metrangolo,[b] Denis Giguère[a,]*

[a]*Département de Chimie, 1045 av. De la Médecine, Université Laval, Québec City, Qc, G1V 0A6, Canada*

[b]*Department of Chemistry, Materials, and Chemical Engineering "Giulio Natta", Politecnico di Milano, Via L. Mancinelli 7, 20131 Milano, Italy*

E-mail: denis.giguere@chm.ulaval.ca

Table of contents

I.	Experimental section	S3
II.	^{19}F NMR spectra prediction	S19
III.	Crystal structure determination	S22
IV.	NMR spectra of compounds	S23
VI.	References	S56

I. Experimental section

General methods

All reactions were carried out under an argon atmosphere with dry solvents under anhydrous conditions, unless otherwise noted. Dry dichloromethane (CH_2Cl_2) was obtained by passing commercially available pre-dried, oxygen-free formulations through activated alumina columns using a Vacuum Atmospheres Inc. Solvent Purification System. Yields refer to chromatographically and spectroscopically (^{1}H NMR) homogeneous materials, unless otherwise stated. Reagents were purchased at the highest commercial quality available and used without further purification, unless otherwise stated. Reactions were monitored by thin-layer chromatography (TLC) carried out on 0.25 mm E. Merck silica gel plates (60F-254) using UV light as visualizing agent and charring with a $KMnO_4$ solution (1.5 g of $KMnO_4$, 10 g K_2CO_3, and 1.25 mL 10 % NaOH in 200 mL of water), or a phenol solution (3 g phenol, 5 mL conc. H_2SO_4 in 95 mL of EtOH), followed by heating with a heatgun as developing agents. SiliaFlash® P60 (particle size 40–63 mm, 230– 400 mesh) was used for flash column chromatography. NMR spectra were recorded on an Agilent DD2 spectrometer (at 500 MHz for ^{1}H, 470 MHz for ^{19}F, and 126 MHz for ^{13}C) and calibrated using residual undeuterated solvent peaks ($CDCl_3$ ^{1}H δ = 7.26 ppm, ^{13}C δ = 77.16 ppm) as an internal reference. Coupling constants (*J*) are reported in Hertz (Hz), and the following abbreviations were used to designate multiplicities: s = singlet, d = doublet, t = triplet, q= quartet, p = quintet, m = multiplet, br = broad. Assignments of NMR signals were made by homonuclear (COSY) and heteronuclear (HSQC, HMBC, and ^{19}F gc2HSQC) two-dimensional correlation spectroscopy. Infrared (IR) spectra were recorded using an ABB Bomem MB-Series Arid Zone FTIR MB-155 Spectrometer, with a ZnSe crystal plate. The absorptions are given in wavenumbers (cm^{-1}). High resolution mass spectra (HRMS) were measured with an Agilent 6210 LC Time of Flight mass spectrometer in electrospray mode (ESI). Either protonated molecular ions $[M + nH]^{n+}$, sodium adducts $[M + Na]^+$, ammonium adducts $[M + NH_4]^+$ or deprotonated molecular ions $[M - nH]^{n-}$ were used for empirical formula confirmation. Optical rotations were recorded on a JASCO DIP-360 digital polarimeter at 589 nm and are reported in units of 10^{-1} (deg cm^2 g^{-1}). Melting points were measured on a Stanford Research System OptiMelt MPA100 151 automated melting point apparatus.

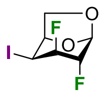

11

1,6-Anhydro-2,3,4-trideoxy-2,3-difluoro-4-iodo-β-D-galactopyranose (11). To a stirred solution of 1,6-anhydro-2,3-dideoxy-2,3-difluoro-β-D-glucopyranose **6**[1] (49.3 mg, 0.2968 mmol, 1.0 equiv.) in CH_2Cl_2 (1.5 mL) at 0 °C, were added pyridine (72.0 μL, 0.4451 mmol, 3.0 equiv.) and Tf_2O (75.0 μL, 0.4451 mmol, 1.5 equiv.). The mixture was stirred at room temperature for 30 min and then quenched with water. The mixture was extracted with CH_2Cl_2, and the combined organic phases were successively washed with an aqueous 1 M HCl solution and brine. The organic solution was dried over $MgSO_4$, filtered, and concentrated under reduced pressure. To a stirred solution of the crude triflate in CH_2Cl_2 (3.0 mL) was added a TBAI (329 mg, 0.8903 mmol, 3.0 equiv.). The mixture was stirred at a room temperature for 18 h and then quenched with water. The mixture was extracted with CH_2Cl_2, and the combined organic phases were successively washed with brine. The organic solution was dried over $MgSO_4$, filtered, and concentrated under reduced pressure. The crude residue was purified by flash column chromatography (silica gel, EtOAc/hexanes, 3:97) to give **11** as a white amorphous solid (13.4 mg, 0.04855 mmol, 16% over 2 steps). R_f = 0.31 (silica, acetone/toluene 1:19); $[\alpha]_D^{25}$ = +91.3 (c 0.5, $CHCl_3$); IR (ATR, diamond crystal) *v* 2988, 2962, 1745, 1219, 1205, 932 cm^{-1}; R_f = 0.45 (silica, EtOAc/hexanes 3:17); $[\alpha]_D^{25}$ = +9.15 (c 0.6, $CHCl_3$); IR (ATR, ZnSe) *v* 2961, 2924, 2851, 1323, 1130, 1045, 1030 cm^{-1}; ^1H NMR (500 MHz, $CDCl_3$) δ 5.73 (t, $^3J_{H1–H2}$ = $^3J_{H1–F2}$ = 1.9 Hz, 1H, H1), 4.69 (dddq, $^3J_{H3–F3}$ = 44.6 Hz, $^3J_{H3–F2}$ = 9.0 Hz, $^3J_{H3–H4}$ = 3.9 Hz, $^3J_{H3–H2}$ = 1.6 Hz, 1.6, 1.6 Hz, 1H, H3), 4.68 – 4.65 (m, 1H, H5), 4.59 (ddt, $^3J_{H2–F2}$ = 44.2 Hz, $^3J_{H2–F3}$ = 9.7 Hz, $^3J_{H2–H1}$ = $^3J_{H2–H3}$ = 2.0 Hz, 1H, H3), 4.57 (dtt, $^3J_{H4–F3}$ = 33.6 Hz, $^3J_{H4–H3}$ = 3.9 Hz, 3.7, 1.6, 1.6 Hz, 1H, H4), 4.39 (d, $^3J_{H6a–H6b}$ = 7.8 Hz, 1H, H6a), 3.79 (ddt, $^3J_{H6b–H6a}$ = 7.8 Hz, 5.2, 1.7, 1.7 Hz, 1H, H6b) ppm; ^{13}C {^1H} NMR (126 MHz, $CDCl_3$) δ 98.26 (d, $^2J_{C1–F2}$ = 25.3 Hz, 1C, C1), 86.77 (dd, $^1J_{C3–F3}$ = 184.0 Hz, $^2J_{C3–F2}$ = 33.4 Hz, 1C, C3), 84.63 (dd, $^1J_{C2–F2}$ = 184.6 Hz, $^2J_{C2–F3}$ = 30.5 Hz, 1C, C2), 76.33 (d, $^3J_{C5–F3}$ = 2.0 Hz, 1C, C5), 65.33 (d, $^4J_{C6–F3}$ = 2.9 Hz, 1C, C6), 22.82 (d, $^2J_{C4–F3}$ = 19.1 Hz, 1C, C4) ppm; ^{19}F NMR (470 MHz, $CDCl_3$) δ -177.90 (dddd, $^2J_{F3–H3}$ = 44.6 Hz, $^3J_{F3–H4}$ = 33.6 Hz, $^3J_{F3–F2}$ = 14.0 Hz, $^2J_{F3–H2}$ = 9.5 Hz, 1F, F3), -193.27 (dddd, $^2J_{F2–H2}$ = 44.2 Hz, $^2J_{F2–F3}$ = 14.1 Hz, $^2J_{F2–H3}$ = 9.1 Hz, $^2J_{F2–H1}$ = 1.9 Hz, 1F, F2) ppm; the compound does not ionize.

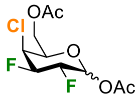

13

1,6-Di-*O*-acetyl-4-chloro-2,3,4-trideoxy-2,3-difluoro-α/β-D-galactopyranose (13). To a stirred solution of 1,6-anhydro-2,3-dideoxy-2,3-difluoro-β-D-glucopyranose **6**[1] (38.7 mg, 0.2330 mmol, 1.0 equiv.) in CH_2Cl_2 (1.2 mL) at 0 °C were added pyridine (56.6 µL, 0.6990 mmol, 3.0 equiv.) and Tf_2O (58.8 µL, 0.3495 mmol, 1.5 equiv.). The mixture was stirred at room temperature for 30 min and then quenched with water. The mixture was extracted with CH_2Cl_2, and the combined organic phases were successively washed with an aqueous 1 M HCl solution and brine. The organic solution was dried over $MgSO_4$, filtered, and concentrated under reduced pressure. To a stirred solution of the crude triflate in CH_2Cl_2 (2.3 mL) was added a TBACl (165 mg, 0.5937 mmol, 2.5 equiv.). The mixture was stirred at a room temperature for 24 h. The mixture was then cooled to 0 °C, and Ac_2O (0.66 mL, 6.9900 mmol, 30 equiv.) and H_2SO_4 (0.13 mL, 2.3300 mmol, 10 equiv.) were added. The mixture was stirred at room temperature for 18 h, then cooled to 0 °C. NaOAc (382 mg, 4.6600 mmol, 20 equiv.) was added and the mixture was stirred for an additional 20 min. Water was added and the mixture was extracted with CH_2Cl_2. The combined organic phases were successively washed with a saturated aqueous $NaHCO_3$ solution and brine. The organic solution was dried over $MgSO_4$, filtered, and concentrated under reduced pressure. The crude residue was purified by flash column chromatography (silica gel, acetone/toluene, 1:19) to give an anomeric mixture (α/β, 4.7:1) of **13** as a thick colorless oil (54.9 mg, 0.1915 mmol, 82% over 3 steps). R_f = 0.29 (silica, acetone/toluene 1:19); $[\alpha]_D^{25}$ = +105.4 (c 1.0, $CHCl_3$); IR (ATR, diamond crystal) *ν* 2953, 2922, 2853, 1745, 1215, 1157 cm^{-1}; only the α anomer has been attributed in ^{13}C NMR; ^1H NMR (500 MHz, $CDCl_3$) δ 6.46 (dd, $^3J_{H1–H2}$ = 4.1 Hz, $^4J_{H1–F3}$ = 4.0 Hz, 1H, H1α), 5.72 (dd, $^3J_{H1–H2}$ = 7.6 Hz, 5.0 Hz, 1H, 1Hβ), 5.17 – 4.99 (m, 2H, H2α, H3α), 4.93 – 4.73 (m, 2H, H2β, H3β), 4.57 (dddd, *J* = 4.2, 4.0, 2.8, 1.2 Hz, 1H, H4α), 4.50 (tdd, *J* = 3.7, 3.7, 2.0, 1.3 Hz, 1H, H4β), 4.36 – 4.20 (m, 4H, H5α, H5β, H6aα, H6aβ), 4.23 (dd, $^2J_{H6b–H6a}$ = 10.3 Hz, $^3J_{H6b–H5}$ = 5.3 Hz, 1H, H6bα), 4.04 (dddd, *J* = 6.5, 5.9, 1.6, 1.4 Hz, 1H, H6bβ), 2.17 (s, 3H, $COCH_3$β), 2.17 (s, 3H, $COCH_3$α), 2.09 (s, 3H, $COCH_3$β), 2.09 (s, 3H, $COCH_3$α) ppm; ^{13}C {^1H} NMR (126 MHz, $CDCl_3$) δ 170.51 (s, 1C, $COCH_3$), 168.54 (s, 1C, $COCH_3$), 89.10 (dd, $^2J_{C1–F2}$ = 22.0 Hz, $^3J_{C1–F3}$ = 9.1 Hz, 1C, C1), 86.29 (dd, $^1J_{C3–F3}$ = 195.5 Hz, $^2J_{C3–F2}$ = 19.6 Hz, 1C, C3), 84.84 (dd, $^1J_{C2–F2}$ = 191.2 Hz, $^2J_{C2–F3}$ = 18.6 Hz, 1C, C2), 68.61 (d, $^3J_{C5–F3}$ = 4.0 Hz, 1C, C5), 62.68 (d, $^4J_{C6–F3}$ = 2.7 Hz, 1C, C6), 58.43 (dd, $^2J_{C4–F3}$ = 17.0 Hz, $^3J_{C4–F2}$ = 7.4 Hz, 1C, C4), 20.94 (s, 1C, $COCH_3$), 20.85 (s, 1C, $COCH_3$) ppm; ^{19}F NMR (470 MHz, $CDCl_3$) δ -192.07 (dtd, *J* = 48.1, 13.5, 13.5, 3.3 Hz, 1F,

F3β), -196.17 (dtt, J = 47.7, 13.6, 13.4, 4.2, 4.2 Hz, 1F, F3α), -210.87 – -211.07 (m, 1F, F2β), -212.71 – -212.90 (m, 1F, F2α) ppm; HRMS calcd for $C_{10}H_{17}ClF_2NO_5^+$ $[M + NH_4]^+$ 304.0758 found 304.0757.

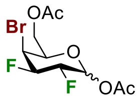

14

1,6-Di-*O*-acetyl-4-bromo-2,3,4-trideoxy-2,3-difluoro-α/β-D-galactopyranose (14). To a stirred solution of 1,6-anhydro-2,3-dideoxy-2,3-difluoro-β-D-glucopyranose **6**[1] (32.1 mg, 0.1932 mmol, 1.0 equiv.) in CH_2Cl_2 (1.0 mL) at 0 °C were added pyridine (47.0 μL, 0.5797 mmol, 3.0 equiv.) and Tf_2O (48.8 μL, 0.1898 mmol, 1.5 equiv.). The mixture was stirred at room temperature for 30 min and then quenched with water. The mixture was extracted with CH_2Cl_2, and the combined organic phases were successively washed with an aqueous 1 M HCl solution and brine. The organic solution was dried over $MgSO_4$, filtered, and concentrated under reduced pressure. To a stirred solution of the crude triflate in CH_2Cl_2 (1.9 mL) was added a TBABr (187 mg, 0.5796 mmol, 3 equiv.). The mixture was stirred at a room temperature for 24 h. The mixture was then cooled to 0 °C, and Ac_2O (0.55 mL, 5.7960 mmol, 30 equiv.) and H_2SO_4 (0.1 mL, 1.9320 mmol, 10 equiv.) were added. The mixture was stirred at room temperature for 18 h, then cooled to 0 °C. NaOAc (317 mg, 3.8640 mmol, 20 equiv.) was added and the mixture was stirred for an additional 20 min. Water was added and the mixture was extracted with CH_2Cl_2. The combined organic phases were successively washed with a saturated aqueous $NaHCO_3$ solution and brine. The organic solution was dried over $MgSO_4$, filtered, and concentrated under reduced pressure. The crude residue was purified by flash column chromatography (silica gel, acetone/toluene, 1:19) to give an anomeric mixture (α/β, 3.4:1) of **14** as a thick colorless oil (41.5 mg, 0.1242 mmol, 64% over 3 steps). R_f = 0.31 (silica, acetone/toluene 1:19); $[\alpha]_D^{25}$ = +91.3 (c 0.5, $CHCl_3$); IR (ATR, diamond crystal) v 2988, 2962, 1745, 1219, 1205, 932 cm^{-1}; ^1H NMR (500 MHz, $CDCl_3$) δ 6.45 (t, $^3J_{H1–H2}$ = $^3J_{H1–F2}$ = 4.2 Hz, 1H, H1α), 5.73 (dd, $^3J_{H1–H2}$ = 7.7 Hz, $^3J_{H1–F2}$ = 5.0 Hz, 1H, H1β), 5.09 (dddd, $^2J_{H2–F2}$ = 49.2 Hz, $^3J_{H2–F3}$ = 10.7 Hz, $^3J_{H2–H3}$ = 9.2 Hz, $^3J_{H2–H1}$ = 4.2 Hz, 1H, H2α), 4.93 (dddd, $^3J_{H3–F3}$ = 50.1 Hz, $^3J_{H3–F2}$ = 11.6 Hz, $^3J_{H3–H2}$ = 9.2 Hz, $^3J_{H3–H4}$ = 4.3 Hz, 1H, H3α), 4.85 (dddd, $^2J_{H2–F2}$ = 51.1 Hz, $^3J_{H2–F3}$ = 12.1 Hz, $^3J_{H2–H3}$ = 8.7 Hz, $^3J_{H2–H1}$ = 8.0 Hz, 1H, H2β), 4.74 (dddd, $^2J_{H3–F3}$ = 48.6 Hz, $^3J_{H3–F2}$ = 13.2 Hz, $^3J_{H3–H2}$ = 8.8 Hz, $^3J_{H3–H4}$ = 4.4 Hz, 1H, H3β), 4.58 (dddd, $^3J_{H4–H3}$ = 4.2 Hz, $^3J_{H4–F3}$ = 3.2 Hz, $^4J_{H4–F2}$ = 3.1 Hz, $^3J_{H4–H5}$ = 1.4 Hz, 1H, H4α), 4.52 (dddd, $^3J_{H4–H3}$ = 4.6 Hz, $^3J_{H4–F3}$ = 3.5 Hz, $^3J_{H4–H5}$ = 2.0 Hz, $^4J_{H4–H6a}$ = 1.2 Hz, 1H, H4β), 4.33 (ddd, $^2J_{H6a–H6b}$ = 11.6 Hz, $^3J_{H6a–H5}$ = 6.5 Hz, $^4J_{H6a–H4}$ = 1.2 Hz, 1H, H6aβ), 4.30 (ddd, $^2J_{H6a–H6b}$ = 10.9 Hz,

$^{3}J_{H6a-H5}$ = 6.1 Hz, 1.5 Hz, 1H, H6aα), 4.24 (dd, $^{2}J_{H6b-H6a}$ = 11.6 Hz, $^{3}J_{H6b-H5}$ = 5.7 Hz, 1H, H6bβ), 4.20 (dd, $^{2}J_{H6b-H6a}$ = 11.0 Hz, $^{3}J_{H6b-H5}$ = 6.0 Hz, 1H, H6bα), 4.16 (tt, $^{3}J_{H5-H6a}$ = $^{3}J_{H5-H6b}$ = 6.0 Hz, $^{3}J_{H5-H4}$ = $^{3}J_{H1-H2}$ = 1.5 Hz, 1H, H5α), 3.87 (ddt, $^{3}J_{H5-H6b}$ = 6.7 Hz, $^{3}J_{H5-H6a}$ = 5.6 Hz, $^{3}J_{H5-H4}$ = $^{3}J_{H1-H2}$ = 1.6 Hz, 1H, H5β), 2.20 (s, 3H, $COCH_3$β), 2.17 (s, 3H, $COCH_3$α), 2.09 (s, 3H, $COCH_3$β), 2.09 (s, 3H, $COCH_3$α) ppm; ^{13}C {^{1}H} NMR (126 MHz, $CDCl_3$) δ 170.51 (1C, $COCH_3$β), 170.51 (1C, $COCH_3$α), 168.96 (1C, $COCH_3$β), 168.51 (1C, $COCH_3$α), 91.39 (dd, $^{2}J_{C1-F2}$ = 25.4 Hz, $^{3}J_{C1-F2}$ = 10.6 Hz, 1C, C1β), 89.14 (dd, $^{2}J_{C1-F2}$ = 22.0 Hz, $^{3}J_{C1-F3}$ = 9.0 Hz, 1C, C1α), 88.43 (dd, $^{1}J_{C3-F3}$ = 196.7 Hz, $^{2}J_{C3-F2}$ = 20.0 Hz, 1C, C3β), 88.22 (dd, $^{1}J_{C2-F2}$ = 187.4 Hz, $^{2}J_{C2-F3}$ = 19.1 Hz, 1C, C2β), 85.92 (dd, $^{2}J_{C3-F3}$ = 195.0 Hz, $^{2}J_{C3-F2}$ = 20.0 Hz, 1C, C3α), 85.79 (dd, $^{1}J_{C2-F2}$ = 191.3 Hz, $^{2}J_{C2-F3}$ = 18.2 Hz, 1C, C2α), 70.82 (d, $^{3}J_{C5-F3}$ = 4.4 Hz, 1C, C5β), 68.24 (d, $^{3}J_{C5-F3}$ = 4.0 Hz, 1C, C5α), 64.27 (d, $^{4}J_{C6-F3}$ = 2.7 Hz, 1C, C6α), 64.06 (d, $^{4}J_{C6-F3}$ = 3.3 Hz, 1C, C6β), 50.95 (dd, $^{2}J_{C4-F3}$ = 16.9 Hz, $^{3}J_{C4-F2}$ = 7.3 Hz, 1C, C4α), 49.88 (dd, $^{2}J_{C4-F3}$ = 16.7 Hz, $^{3}J_{C4-F2}$ = 7.8 Hz, 1C, C4β), 29.85 (1C, $COCH_3$β), 20.94 (1C, $COCH_3$α), 20.92 (1C, $COCH_3$β), 20.84 (1C, $COCH_3$α) ppm; ^{19}F NMR (470 MHz, $CDCl_3$) δ -186.79 (dtd, $^{2}J_{F3-H3}$ = 49.1 Hz, $^{3}J_{F3-F2}$ = $^{3}J_{F3-H2}$ = 13.2 Hz, $^{3}J_{F3-H4}$ = 3.5 Hz, 1F, F3β), -190.85 (ddddd, $^{2}J_{F3-H3}$ = 49.9 Hz, $^{3}J_{F3-F2}$ = 14.2 Hz, $^{3}J_{F3-H2}$ = 10.7 Hz, 3.8, $^{3}J_{F3-H4}$ = 3.2 Hz, 1F, F3α), -210.70 (dtdd, $^{2}J_{F2-H2}$ = 51.0 Hz, $^{3}J_{F2-F3}$ = $^{3}J_{F2-H3}$ = 14.0 Hz, $^{3}J_{F2-H1}$ = 5.1 Hz, 2.2 Hz, 1F, F2β), -212.48 (dddd, $^{2}J_{F2-H2}$ = 49.2 Hz, $^{3}J_{F2-F3}$ = 14.2 Hz, $^{3}J_{F2-H3}$ = 11.5 Hz, $^{4}J_{F2-H4}$ = 2.8 Hz, 1F, F2α) ppm; HRMS calcd for $C_{10}H_{17}BrF_2NO_3^+$ [M + NH_4]$^+$ 348.0253 found 348.0251.

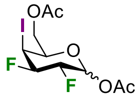

15

1,6-Di-*O*-acetyl-2,3,4-trideoxy-2,3-difluoro-4-iodo-α/β-D-galactopyranose (15). To a stirred solution of 1,6-anhydro-2,3,4-trideoxy-2,3-difluoro-4-iodo-β-D-galactopyranose **11** (9.0 mg, 0.03261 mmol, 1.0 equiv.) in CH_2Cl_2 (1.0 mL) at 0 °C, were added Ac_2O (92 μL, 0.9782 mmol, 30 equiv.) and H_2SO_4 (17 μL, 0.3261 mmol, 10 equiv.). The mixture was stirred at room temperature for 18 h, then cooled to 0 °C. NaOAc (53.5 mg, 0.6521 mmol, 20 equiv.) was added and the mixture was stirred for an additional 20 min. Water was added and the mixture was extracted with CH_2Cl_2. The combined organic phases were successively washed with a saturated aqueous $NaHCO_3$ solution and brine. The organic solution was dried over $MgSO_4$, filtered, and concentrated under reduced pressure. The crude residue was purified by flash column chromatography (silica gel, acetone/toluene, 1:24) to give an anomeric mixture (α/β, 4.3:1) of **15** as a yellow thick oil (9.7 mg, 0.02565 mmol, 79%). R_f = 0.27 (silica, acetone/toluene 1:19); $[\alpha]_D^{25}$ = +92.4

(c 0.5, $CHCl_3$); IR (ATR, ZnSe) ν 2957, 2924, 2361, 1747, 1221, 1082, 1055 cm^{-1}; ^{1}H NMR (500 MHz, $CDCl_3$) δ 6.40 (t, $^{3}J_{H1-H2}$ = $^{3}J_{H1-H2}$ = 4.2 Hz, 1H, H1α), 5.75 (dd, $^{3}J_{H1-H2}$ = 7.8 Hz, $^{3}J_{H1-F2}$ = 5.2 Hz, 1H, H1β), 5.05 (dddd, $^{2}J_{H2-F2}$ = 49.3 Hz, $^{3}J_{H2-F3}$ = 10.6 Hz, $^{3}J_{H2-H3}$ = 9.1 Hz, $^{3}J_{H2-H1}$ = 4.3 Hz, 1H, H2α), 4.84 (ddt, $^{2}J_{H2-F2}$ = 51.6 Hz, $^{3}J_{H2-F3}$ = 11.6 Hz, $^{3}J_{H2-H1}$ = $^{3}J_{H2-H3}$ = 8.3 Hz, 1H, H2β), 4.60 (dq, $^{3}J_{H4-H3}$ = 4.7 Hz, 2.4, 2.4, 2.4 Hz, 1H, H4α), 4.55 (dq, $^{3}J_{H4-H3}$ = 4.1 Hz, $^{3}J_{H4-H5}$ = 2.0 Hz, 2.0, 2.0 Hz, 1H, H4β), 4.36 (dddd, $^{2}J_{H3-F3}$ = 51.3 Hz, $^{3}J_{H3-F2}$ = 11.5 Hz, $^{3}J_{H3-H2}$ = 9.1 Hz, $^{3}J_{H3-H4}$ = 4.7 Hz, 1H, H3α), 4.28 (ddd, $^{2}J_{H6a-H6b}$ = 11.4 Hz, $^{3}J_{H6a-H5}$ = 6.4 Hz, 1.4 Hz, 1H, H6aα), 4.25 (dddd, $^{2}J_{H3-F3}$ = 50.1 Hz, $^{3}J_{H3-F2}$ = 13.4 Hz, $^{3}J_{H3-H2}$ = 8.6 Hz, $^{3}J_{H3-H4}$ = 4.8 Hz, 1H, H3β), 4.33 – 4.30 (m, 1H, H6aβ), 4.14 (dd, $^{2}J_{H6b-H6a}$ = 11.6 Hz, $^{3}J_{H6b-H5}$ = 5.4 Hz, 1H, H6bβ), 4.09 (dd, $^{2}J_{H6b-H6a}$ = 11.5 Hz, $^{3}J_{H6b-H5}$ = 5.8 Hz, 1H, H6bα), 3.51 (tt, $^{3}J_{H5-H6a}$ = $^{3}J_{H5-H6b}$ = 6.2 Hz, 1.8, 1.8 Hz, 1H, H5α), 3.23 (ddt, $^{3}J_{H5-H6a}$ = 7.0 Hz, $^{3}J_{H5-H6b}$ = 5.5 Hz, $^{3}J_{H5-H4}$ = 1.7 Hz, 1.7 Hz, 1H, H5β), 2.19 (s, 3H, $COCH_3$β), 2.16 (s, 3H, $COCH_3$α), 2.09 (s, 3H, $COCH_3$β), 2.08 (s, 3H, $COCH_3$α) ppm; ^{13}C {^{1}H} NMR (126 MHz, $CDCl_3$) δ 170.50 (1C, $\mathit{C}OCH_3$α), 170.48 (1C, $\mathit{C}OCH_3$β), 168.95 (1C, $\mathit{C}OCH_3$β), 168.50 (1C, $\mathit{C}OCH_3$α), 91.52 (dd, $^{2}J_{C1-F2}$ = 25.4 Hz, $^{3}J_{C1-F3}$ = 10.5 Hz, 1C, C1β), 89.81 (dd, $^{1}J_{C2-F2}$ = 187.7 Hz, $^{2}J_{C2-F3}$ = 18.6 Hz, 1C, C2β), 89.22 (dd, $^{2}J_{C1-F2}$ = 21.9 Hz, $^{3}J_{C1-F2}$ = 8.9 Hz, 1C, C1α), 88.36 (dd, $^{1}J_{C3-F3}$ = 195.2 Hz, $^{2}J_{C3-F2}$ = 19.6 Hz, 1C, C3β), 87.72 (dd, $^{1}J_{C2-F2}$ = 192.1 Hz, $^{2}J_{C2-F3}$ = 17.6 Hz, 1C, C2α), 85.86 (dd, $^{1}J_{C3-F3}$ = 194.0 Hz, $^{2}J_{C3-F2}$ = 20.0 Hz, 1C, C3α), 70.70 (d, $^{3}J_{C5-F3}$ = 4.8 Hz, 1C, C5β), 68.07 (d, $^{3}J_{C5-F3}$ = 4.3 Hz, 1C, C5α), 67.25 (d, $^{4}J_{C6-F3}$ = 2.9 Hz, 1C, C6α), 66.89 (d, $^{4}J_{C6-F3}$ = 3.4 Hz, 1C, C6β), 31.53 (dd, $^{2}J_{C4-F3}$ = 16.9 Hz, $^{3}J_{C4-F2}$ = 6.5 Hz, 1C, C4α), 30.08 (dd, $^{2}J_{C4-F3}$ = 16.9 Hz, $^{3}J_{C4-F2}$ = 7.0 Hz, 1C, C4β), 20.94(1C, $CO\mathit{C}H_3$α), 20.91 (1C, $CO\mathit{C}H_3$β), 20.81 (1C, $CO\mathit{C}H_3$α), 20.81 (1C, $CO\mathit{C}H_3$β) ppm; ^{19}F NMR (470 MHz, $CDCl_3$) δ -178.08 (dt, $^{2}J_{F3-H3}$ = 50.1 Hz, $^{3}J_{F3-F2}$ = $^{2}J_{F3-H2}$ = 12.8 Hz, 1F, F3β), -182.10 (dt, $^{2}J_{F3-H3}$ = 51.0 Hz, $^{3}J_{F3-F2}$ = 14.3 Hz, 12.0 Hz, 1F, F3α), -210.27 (dtd, $^{2}J_{F2-H2}$ = 51.4 Hz, $^{3}J_{F2-F3}$ = 13.8 Hz, $^{3}J_{F2-H3}$ = 13.7 Hz, $^{3}J_{F2-H1}$ = 5.0 Hz, 1F, F2β), -211.92 (dddd, $^{2}J_{F2-H2}$ = 49.4 Hz, $^{3}J_{F2-F3}$ = 14.3 Hz, $^{3}J_{F2-H3}$ = 11.5 Hz, 2.8 Hz, 1F, F2α) ppm; HRMS calcd for $C_{10}H_{17}F_2INO_3^+$ $[M + NH_4]^+$ 396.0114 found 396.0111.

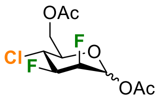

30

1,6-Di-*O*-acetyl-4-chloro-2,3,4-trideoxy-2,3-difluoro-α/β-D-mannopyranose (30). To a stirred solution of 1,6-anhydro-2,3-dideoxy-2,3-difluoro-β-D-glucopyranose **6**[1] (30.0 mg, 0.1806 mmol, 1.0 equiv.) in CH_2Cl_2 (2.0 mL) at 0 °C were added pyridine (0.141 mL, 1.752 mmol, 9.7 equiv.) and Tf_2O (0.0696 mL,

0.4154 mmol, 2.3 equiv.). The mixture was stirred at room temperature for 30 min and then quenched with water. The mixture was extracted with CH_2Cl_2, and the combined organic phases were successively washed with an aqueous 1 M HCl solution and brine. The organic solution was dried over $MgSO_4$, filtered, and concentrated under reduced pressure. To a stirred solution of the crude triflate in CH_3CN (3.5 mL) was added TBACl (93 mg, 0.3354 mmol, 2.0 equiv.) at 80 °C for 48 h. The volatiles were evaporated under a stream of air and the crude was dissolved in CH_2Cl_2 (0.5 mL). The mixture was then cooled to 0 °C, and Ac_2O (0.51 mL, 5.417 mmol, 30 equiv.) and H_2SO_4 (0.10 mL, 1.806 mmol, 10 equiv.) were added. The mixture was stirred at room temperature for 18 h, then cooled to 0 °C. NaOAc (296 mg, 3.612 mmol, 20 equiv.) was added and the mixture was stirred for an additional 20 min. Water was added and the mixture was extracted with CH_2Cl_2. The combined organic phases were successively washed with a saturated aqueous $NaHCO_3$ solution and brine. The organic solution was dried over $MgSO_4$, filtered, and concentrated under reduced pressure. The crude residue was purified by flash column chromatography (silica gel, EtOAc/hexanes, 0:1 → 1:1) to give an anomeric mixture (α/β, 2.3:1) of **30** as a yellow oil (33.5 mg, 0.1169 mmol, 65% over 3 steps). R_f = 0.5 (silica, EtOAc/hexanes 2:3); $[\alpha]_D^{25}$ = +48 (*c* = 2.5, $CHCl_3$); IR (ATR, diamond crystal) *v* 2964, 1742, 1373, 1215, 1141, 977 cm^{-1}; ^1H NMR (500 MHz, $CDCl_3$) δ 6.33 (ddd, $^3J_{H1–F2}$ = 6.6 Hz, $^3J_{H1–H2}$ = 4.9 Hz, 2.2 Hz, 1H, H1α), 5.74 (dd, $^3J_{H1–F2}$ = 18.4 Hz, $^3J_{H1–H2}$ = 2.4 Hz, 1H, H1β), 4.96 (ddd, $^2J_{H2–F2}$ = 51.4 Hz, $^3J_{H2–F3}$ = 7.3 Hz, $^3J_{H2–H1}$ = 2.4 Hz, 0.63 Hz, 1H, H2β), 4.92 – 4.77 (m, 1H, H2α), 4.79 (dddd, $^2J_{H3–F3}$ = 46.1 Hz, $^3J_{H3–F2}$ =26.0 Hz, $^3J_{H3–H4}$ = 10.2 Hz, $^3J_{H3–H2}$ = 2.7 Hz, 1H, H3α), 4.61 (dddd, $^2J_{H3–F3}$ = 45.4 Hz, $^3J_{H3–F2}$ =25.1 Hz, $^3J_{H3–H4}$ = 10.1 Hz, $^3J_{H3–H2}$ = 2.5 Hz, 1H, H3β), 4.51 (ddd, $^2J_{H6a–H6b}$ = 12.4 Hz, $^3J_{H6a–H5}$ = 1.9 Hz, $^4J_{H6a–H4}$ = 1.6 Hz, 1H, H6aα), 4.46 (dt, $^2J_{H6a–H6b}$ = 12.4 Hz, $^3J_{H6a–H5}$ = $^4J_{H6a–H4}$ = 2.1 Hz, 1H, H6aα), 4.38 (dd, $^2J_{H6b–H6a}$ = 12.4 Hz, $^3J_{H6b–H5}$ = 4.7 Hz, 1H, H6bβ), 4.38 (dd, $^2J_{H6b–H6a}$ = 12.4 Hz, $^3J_{H6b–H5}$ = 4.1 Hz, 1H, H6bα), 4.31 (tdd, $^3J_{H4–H3}$ = $^3J_{H4–H5}$ = 10.2 Hz, $^3J_{H4–F3}$ = 9.1 Hz, $^4J_{H4–H6a}$ = 1.3 Hz, 1H, H4α), 4.22 (tdd, $^3J_{H4–H3}$ = $^3J_{H4–H5}$ = 10.2 Hz, $^3J_{H4–F3}$ = 9.3 Hz, $^4J_{H4-H6}$ = 1.5 Hz, 1H, H4β), 4.03 (dddt, $^3J_{H5-H4}$ = 11.4 Hz, 4.0, 1.9, 0.9 Hz, 1H, H5α), 3.80 – 3.74 (m, 1H, H5β), 2.20 (s, 3H, COC*H*3α), 2.16 (s, 3H, COC*H*3β), 2.11 (s, 6H, COC*H*3α, COC*H*3β) ppm; ^{13}C {^1H} NMR (126 MHz, $CDCl_3$) δ 170.50 (2C, *C*OCH3α, *C*OCH3β), 168.53 (1C, *C*OCH3β), 167.78 (1C, *C*OCH3α), 90.86 (dd, $^1J_{C3–F3}$ = 196.0 Hz, $^2J_{C3–F2}$ = 17.2 Hz, 1C, C3β), 90.21 – 89.90 (m, 1C, C1β), 90.47 (dd, $^2J_{C1–F2}$ = 30.3 Hz, $^3J_{C1–F3}$ = 7.0 Hz, 1C, C1α), 89.11 (dd, $^1J_{C3–F3}$ = 194.0 Hz, $^2J_{C3–F2}$ = 17.2 Hz, 1C, C3α), 86.39 (dd, $^1J_{C2–F2}$ = 193.4 Hz, $^2J_{C2–F3}$ = 17.5 Hz, 1C, C2β), 85.36 (dd, $^1J_{C2–F2}$ = 184.3 Hz, $^2J_{C2–F3}$ = 16.9 Hz, 1C, C2α), 74.49 (d, $^3J_{C5–F3}$ = 5.5 Hz, 1C, C5β), 72.71 (d, $^3J_{C5–F3}$ = 5.1 Hz, 1C, C5α), 62.20 (d, $^4J_{C6–F3}$ = 2.3 Hz, 1C, C6β), 62.20 (d, $^4J_{C6–F3}$ = 1.9 Hz, 1C, C6α), 52.48 (dd, $^2J_{C4–F3}$ = 19.5 Hz, $^3J_{C4–F2}$ = 1.8 Hz, 1C, C4α), 52.12 (dd,

${}^{2}J_{C4–F3}$ = 19.5 Hz, ${}^{3}J_{C4–F2}$ = 1.9 Hz, 1C, C4β), 20.84 (2C, 2 × CO*C*H3), 20.80 (2C, 2 × CO*C*H3) ppm; ^{19}F NMR (470 MHz, $CDCl_3$) δ -193.74 (dddd, ${}^{2}J_{F3–H3}$ = 45.3 Hz, ${}^{3}J_{F3-F2}$ = 16.3 Hz, ${}^{3}J_{F3–H4}$ = 9.3 Hz, ${}^{3}J_{F3–H2}$ = 7.4 Hz, 1F, F3β), -198.15 (ddddd, ${}^{2}J_{F3–H3}$ = 46.1 Hz, ${}^{3}J_{F3–F3}$ = 16.3 Hz, ${}^{3}J_{F3–H4}$ = 9.0 Hz, ${}^{3}J_{F3–H2}$ = 6.9 Hz, 6.88 Hz 1F, F3α), -205.98 (dddd, ${}^{2}J_{F2–H2}$ = 48.5 Hz, ${}^{3}J_{F2–H3}$ = 25.9 Hz, ${}^{3}J_{F2–F3}$ = 16.4 Hz, ${}^{3}J_{F2–H1}$ = 6.6 Hz, 1F, F2α), -220.99 (ddt, ${}^{2}J_{F2–H2}$ = 51.4 Hz, ${}^{3}J_{F2–H3}$ = 25.2 Hz, ${}^{3}J_{F2-H1}$ = 18.4 Hz, ${}^{3}J_{F2–F3}$ = 16.3 Hz, 1F, F2β) ppm; HRMS calcd for $C_{10}H_{17}ClF_2NO_5^+$ $[M + NH_4]^+$ 304.0758 found 304.0835.

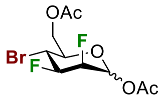

32

1,6-Di-*O*-acetyl-4-bromo-2,3,4-trideoxy-2,3-difluoro-α/β-D-mannopyranose (32). To a stirred solution of 1,6-anhydro-2,3-dideoxy-2,3-difluoro-β-D-glucopyranose **6**[1] (50.0 mg, 0.3010 mmol, 1.0 equiv.) in CH_2Cl_2 (3.5 mL) at 0 °C were added pyridine (0.24 mL, 2.920 mmol, 9.7 equiv.) and Tf_2O (0.12 mL, 0.6923 mmol, 2.3 equiv.). The mixture was stirred at room temperature for 30 min and then quenched with water. The mixture was extracted with CH_2Cl_2, and the combined organic phases were successively washed with an aqueous 1 M HCl solution and brine. The organic solution was dried over $MgSO_4$, filtered, and concentrated under reduced pressure. To a stirred solution of the crude triflate in CH_3CN (6.0 mL) was added TBABr (194 mg, 0.6020 mmol, 2.0 equiv.) at 80 °C for 48 h. The volatiles were evaporated under a stream of air and the crude was dissolved in CH_2Cl_2 (0.8 mL). The mixture was then cooled to 0 °C, and Ac_2O (0.85 mL, 9.029 mmol, 30 equiv.) and H_2SO_4 (0.121 mL, 3.010 mmol, 10 equiv.) were added. The mixture was stirred at room temperature for 18 h, then cooled to 0 °C. NaOAc (494 mg, 6.020 mmol, 20 equiv.) was added and the mixture was stirred for an additional 20 min. Water was added and the mixture was extracted with CH_2Cl_2. The combined organic phases were successively washed with a saturated aqueous $NaHCO_3$ solution and brine. The organic solution was dried over $MgSO_4$, filtered, and concentrated under reduced pressure. The crude residue was purified by flash column chromatography (silica gel, EtOAc/hexanes, 0:1 → 1:1) to give an anomeric mixture (α/β, 2.5:1) of **32** as a yellow oil (65.4 mg, 0.1975 mmol, 66% over 3 steps). R_f = 0.48 (silica, EtOAc/hexanes 1:1); $[\alpha]_D^{25}$ = +309 (*c* = 2.5, $CHCl_3$); IR (ATR, diamond crystal) *v* 3315, 2962, 1742, 1371, 1217, 1140, 976 cm^{-1}; ^{1}H NMR (500 MHz, $CDCl_3$) δ 6.34 (td, ${}^{3}J_{H1–H2}$ = ${}^{3}J_{H1–F2}$ = 5.6 Hz, 2.1 Hz, 1H, H1α), 5.74 (dd, ${}^{3}J_{H1–F2}$ = 18.5 Hz, ${}^{3}J_{H1–H2}$ = 1.9 Hz, 1H, H1β), 4.99 – 4.72 (m, 3H, H2α, H2β, H3α), 4.67 (dddd, ${}^{3}J_{H3–F3}$ = 44.9 Hz, ${}^{3}J_{H3–F2}$ = 24.9 Hz, ${}^{3}J_{H3–H4}$ = 10.4 Hz, ${}^{3}J_{H3–H2}$ = 2.4 Hz, 1H, H3β), 4.55 (dt, ${}^{2}J_{H6a–H6b}$ = 12.3 Hz, ${}^{3}J_{H6a–H5}$ = 1.9 Hz, 1.9 Hz, 1H,

H6aβ), 4.50 (dt, $^{2}J_{H6a–H6b}$ = 12.3 Hz, $^{3}J_{H6a–H5}$ = 2.1 Hz, 2.1 Hz, 1H, H6aα), 4.43 (dd, $^{2}J_{H6b–H6a}$ = 12.3 Hz, $^{3}J_{H6b–H5}$ = 5.8 Hz, 1H, H6bβ), 4.42 (dd, $^{2}J_{H6b–H6a}$ = 12.4 Hz, $^{3}J_{H6b–H5}$ = 4.2 Hz, 1H, H6bα), 4.33 (tdd, $^{3}J_{H4–H3}$ = $^{3}J_{H4–H5}$ = 10.7 Hz, $^{3}J_{H4–F3}$ = 8.2 Hz, 1.3 Hz, 1H, H4α), 4.23 (tdd, $^{3}J_{H4–H3}$ = $^{3}J_{H4–H5}$ = 10.3 Hz, $^{3}J_{H4–F3}$ = 8.6 Hz, 1.3 Hz, 1H, H4β), 4.13 (dddt, $^{3}J_{H5–H4}$ = 10.8 Hz, $^{3}J_{H5–H6b}$ = 4.1 Hz, $^{3}J_{H5–H6a}$ = 2.0 Hz, 1.0, 1.0 Hz, 1H, H5α), 3.86 (dddd, $^{3}J_{H5–H4}$ = 10.6 Hz, $^{3}J_{H5–H6b}$ = 5.5 Hz, $^{3}J_{H5–H6a}$ = 2.3 Hz, 1.1 Hz, 1H, H5β), 2.20 (s, 3H, COC*H*$_3$β), 2.16 (s, 3H, COC*H*$_3$α), 2.11 (s, 6H, COC*H*$_3$α, COC*H*$_3$β) ppm; ^{13}C {^{1}H} NMR (126 MHz, CDCl$_3$) δ 170.62 (2C, *C*OCH$_3$α, *C*OCH$_3$β), 168.67 (1C, *C*OCH$_3$β), 167.91 (1C, *C*OCH$_3$α), 91.03 (dd, $^{1}J_{C3–F3}$ = 195.5 Hz, $^{2}J_{C3–F2}$ = 17.2 Hz, 1C, C3β), 90.64 (dd, $^{2}J_{C1–F2}$ = 30.3 Hz, $^{3}J_{C1–F3}$ = 7.0 Hz, 1C, C1α), 90.15 (dd, $^{2}J_{C1–F2}$ = 15.7 Hz, $^{3}J_{C1–F3}$ = 10.5 Hz, 1C, C1β), 89.08 (dd, $^{1}J_{C3–F3}$ = 193.6 Hz, $^{2}J_{C3–F2}$ = 17.2 Hz, 1C, C3α), 86.52 (dd, $^{1}J_{C2–F2}$ = 194.1 Hz, $^{2}J_{C2–F3}$ = 17.9 Hz, 1C, C2β), 85.38 (dd, $^{1}J_{C2–F2}$ = 185.2 Hz, $^{2}J_{C2–F3}$ = 17.2 Hz, 1C, C2α), 74.59 (d, $^{3}J_{C5–F3}$ = 5.7 Hz, 1C, C5β), 72.77 (d, $^{3}J_{C5–F3}$ = 5.0 Hz, 1C, C5α), 63.07 (d, $^{4}J_{C6–F3}$ = 2.0 Hz, 1C, C6α), 63.01 (d, $^{4}J_{C6–F3}$ = 2.5 Hz, 1C, C6β), 43.13 (dd, $^{2}J_{C4–F3}$ = 19.5 Hz, $^{3}J_{C4–F2}$ = 1.2 Hz, 1C, C4α), 42.49 (dd, $^{2}J_{C4–F3}$ = 19.3 Hz, $^{3}J_{C4–F2}$ = 1.5 Hz, 1C, C4β), 20.91 (1C, CO*C*H$_3$α), 20.89 (1C, CO*C*H$_3$β), 20.86 (2C, CO*C*H$_3$α, CO*C*H$_3$β) ppm; ^{19}F NMR (470 MHz, Chloroform-*d*) δ -189.51 (ddt, $^{2}J_{F3–H3}$ = 45.2 Hz, $^{3}J_{F3–F2}$ = 16.1 Hz, $^{3}J_{F3–H2}$ = $^{3}J_{F3–H4}$ = 8.1 Hz, 1F, F3β), -193.31 (ddq, $^{2}J_{F3–H3}$ = 46.1 Hz, $^{3}J_{F3–F2}$ = 15.8 Hz, $^{3}J_{F3–H4}$ = 8.2 Hz, $^{3}J_{F3–H2}$ = 6.8 Hz, 6.8 Hz, 1F, F3α), -206.29 (dddd, $^{2}J_{F2–H2}$ = 48.9 Hz, $^{3}J_{F2–H3}$ = 26.2 Hz, $^{3}J_{F2–F3}$ = 15.8 Hz, $^{3}J_{F2–H1}$ = 6.0 Hz, 1F, F2α), -221.20 (ddt, $^{2}J_{F2–H2}$ = 51.4, $^{3}J_{F2–H3}$ = 25.0 Hz, $^{3}J_{F2–H1}$ = 17.9 Hz, $^{3}J_{F2–F3}$ = 15.5 Hz, 1F, F3α) ppm; HRMS calcd for $C_{10}H_{17}BrF_2NO_5^+$ [M + NH$_4$]$^+$ 348.0253 found 348.0331.

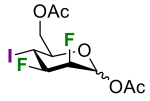

34

1,6-Di-*O*-acetyl-2,3,4-trideoxy-2,3-difluoro-4-iodo-α/β-D-mannopyranose (34). To a stirred solution of 1,6-anhydro-2,3-dideoxy-2,3-difluoro-β-D-glucopyranose **6**[1] (50.0 mg, 0.3010 mmol, 1.0 equiv.) in CH_2Cl_2 (3.5 mL) at 0 °C were added pyridine (0.24 mL, 2.920 mmol, 9.7 equiv.) and Tf_2O (0.12 mL, 0.6923 mmol, 2.3 equiv.). The mixture was stirred at room temperature for 30 min and then quenched with water. The mixture was extracted with CH_2Cl_2, and the combined organic phases were successively washed with an aqueous 1 M HCl solution and brine. The organic solution was dried over $MgSO_4$, filtered, and concentrated under reduced pressure. To a stirred solution of the crude triflate in CH_3CN (6.0 mL) was added TBAI (222 mg, 0.6020 mmol, 2.0 equiv.) at 80 °C for 48 h. The volatiles were evaporated

under a stream of air and the crude was dissolved in CH_2Cl_2 (0.8 mL). The mixture was then cooled to 0 °C, and Ac_2O (0.85 mL, 9.029 mmol, 30 equiv.) and H_2SO_4 (0.121 mL, 3.010 mmol, 10 equiv.) were added. The mixture was stirred at room temperature for 18 h, then cooled to 0 °C. NaOAc (494 mg, 6.020 mmol, 20 equiv.) was added and the mixture was stirred for an additional 20 min. Water was added and the mixture was extracted with CH_2Cl_2. The combined organic phases were successively washed with a saturated aqueous $NaHCO_3$ solution and brine. The organic solution was dried over $MgSO_4$, filtered, and concentrated under reduced pressure. The crude residue was purified by flash column chromatography (silica gel, EtOAc/hexanes, 0:1 → 1:1) to give an anomeric mixture (α/β, 15.7:1) of **34** as a yellow oil (77.1 mg, 0.2039 mmol, 68% over 3 steps). The resulting product was recrystallized from chloroform to give colorless crystals. R_f = 0.43 (silica, EtOAc/hexanes 2:3); m.p. = 116 – 128 °C; $[\alpha]_D^{25}$ = +53.7 (*c* = 2.5, $CHCl_3$); IR (ATR, diamond crystal) *v* 3057, 2928, 1740, 1371, 1215, 1138, 976, 704 cm^{-1}; only the α anomer has been attributed in ^1H NMR and ^{13}C NMR; ^1H NMR (500 MHz, $CDCl_3$) δ 6.36 (td, $^{3}J_{H1–F2}$ = 5.6 Hz, 5.3 Hz, $^{3}J_{H1–H2}$ = 2.5 Hz, 1H, H1), 4.86 (dddt, $^{2}J_{H3–F3}$ = 44.6 Hz, $^{3}J_{H3–F2}$ = 25.5 Hz, $^{3}J_{H3–H4}$ = 11.0 Hz, 2.2, 2.2 Hz, 1H, H4), 4.70 (ddq, $^{2}J_{H2–F2}$ = 49.2 Hz, $^{3}J_{H2–F3}$ = 6.5 Hz, $^{3}J_{H2–H1}$ = 2.3 Hz, 2.3, 2.3 Hz, 1H, H2), 4.58 (ddt, $^{3}J_{H6a–H6b}$ = 12.5 Hz, $^{3}J_{H6a–H5}$ = 2.4 Hz, 2.3, 2.3 Hz, 1H, H6a), 4.49 (ddd, $^{3}J_{H6b–H6a}$ = 12.4 Hz, $^{3}J_{H6b–H5}$ = 4.3 Hz, 2.0 Hz, 1H, H6b), 4.39 (tdd, $^{3}J_{H4–H3}$ = $^{3}J_{H4–H5}$ = 11.1 Hz, $^{3}J_{H4–F3}$ = 6.7 Hz, 1.6 Hz, 1H, H5), 4.25 – 4.19 (m, 1H, H5), 2.17 (s, 3H, $COCH_3$), 2.13 (s, 3H, $COCH_3$) ppm; ^{13}C {^1H} NMR (126 MHz, $CDCl_3$) δ 170.60 (1C, $\mathit{C}OCH_3$), 167.95 (1C, $\mathit{C}OCH_3$), 90.89 (dd, $^{2}J_{C1–F2}$ = 30.1 Hz, $^{3}J_{C1–F3}$ = 7.1 Hz, 1C, C1), 89.85 (dd, $^{1}J_{C3–F3}$ = 192.3 Hz, $^{2}J_{C3–F2}$ = 17.3 Hz, 1C, C3), 84.83 (dd, $^{1}J_{C2–F2}$ = 185.6 Hz, $^{2}J_{C2–F3}$ = 17.5 Hz, 1C, C2), 73.53 (d, $^{3}J_{C5–F3}$ = 5.6 Hz, 1C, C5), 64.66 (d, $^{4}J_{C6–F3}$ = 2.2 Hz, 1C, C6), 20.92 (1C, $CO\mathit{C}H_3$), 20.85 (1C, $CO\mathit{C}H_3$), 20.53 (dd, $^{2}J_{C4–F3}$ = 19.1 Hz, $^{3}J_{C4–F2}$ = 1.0 Hz, 1C, C4) ppm; ^{19}F NMR (470 MHz, Chloroform-*d*) δ -182.46 (ddt, *J* = 43.8, 15.6, 8.0, 8.0 Hz, 1F, F3β), -185.07 (ddt, $^{2}J_{F3–H3}$ = 45.1 Hz, $^{3}J_{F3–F2}$ = 15.6 Hz, $^{3}J_{F3–H2}$ = $^{2}J_{F3–H4}$ = 7.2 Hz, 1F, F3α), -206.96 (dddd, $^{2}J_{F2–H2}$ = 49.0 Hz, $^{3}J_{F2–H3}$ = 25.4 Hz, $^{3}J_{F2–F3}$ = 16.3 Hz, $^{3}J_{F2–H1}$ = 5.7 Hz, 1F, F2α), -221.68 (ddt, *J* = 51.3, 24.9, 16.9, 16.9 Hz, 1F, F2β) ppm; HRMS calcd for $C_{10}H_{17}IF_2NO_5^+$ $[M + NH_4]^+$ 396.0114 found 396.0188.

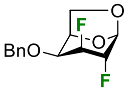

41

1,6-Anhydro-4-*O*-benzyl-2,3-dideoxy-2,3-difluoro-β-D-galactopyranoside (41). To a stirred solution of compound 1,6-anhydro-2,3-dideoxy-2,3-difluoro-β-D-galactopyranose **37**[1] (65.0 mg, 0.3913 mmol) in anhydrous DMF (4 mL, 0.1 M) at 0 °C under a nitrogen atmosphere, were added NaH (60% dispersion in

mineral oil, 23.5 mg, 0.5869 mmol, 1.5 equiv.), BnBr (70 µL, 0.5689 mmol, 1.5 equiv.), and TBAI (36.2 mg, 0.0978 mmol, 0.25 equiv.). The mixture was stirred at room temperature for 2 h and then quenched with water. The mixture was extracted with CH_2Cl_2. The combined organic phases were successively washed with an aqueous 1 M HCl solution and a saturated aqueous $NaHCO_3$ solution. The organic phase was dried over $MgSO_4$, filtered, and concentrated under reduced pressure. The crude oil was purified by flash column chromatography (silica gel, EtOAc/hexanes, 1:9 → 1:4) to give **41** as a colorless oil (86.1 mg, 0.3360 mmol, 86% yield). R_f = 0.57 (silica, EtOAc/hexanes 3:7); $[\alpha]_D^{25} = -56.05$ (c 0.6, $CHCl_3$); IR (ATR, diamond crystal) ν 3032, 2962, 2908, 2876, 1454, 1140, 1038 cm^{-1}; ^{1}H NMR (500 MHz, $cdcl_3$) δ 7.42 – 7.30 (m, 5H, HAr), 5.50 (t, $^{3}J_{H1-H2} = {}^{3}J_{H1-F2}$ = 1.5 Hz, 1H, H1), 4.92 (dddp, $^{2}J_{H3-F3}$ = 47.1 Hz, $^{3}J_{H3-F2}$ = 9.2 Hz, $^{3}J_{H3-H4}$ = 4.7 Hz, $^{3}J_{H3-H2} = {}^{4}J_{H3-H5}$ = 1.6 Hz, 1.6, 1.6 Hz, 1H, H3), 4.75 (d, $^{2}J_{C\underline{H}aHbPh-CHa\underline{H}bPh}$ = 11.8 Hz, 1H, CH_2Ph), 4.61 (d, $^{2}J_{CHa\underline{H}bPh-C\underline{H}aHbPh}$ = 11.7 Hz, 1H, CH_2Ph), 4.60 (ddt, $^{2}J_{H2-F2}$ = 44.1 Hz, $^{3}J_{H2-F3}$ = 11.3 Hz, $^{3}J_{H2-H1} = {}^{3}J_{H2-H3}$ = 1.6 Hz, 1H, H2), 4.49 (ddd, $^{3}J_{H5-H6a}$ = 5.4 Hz, $^{3}J_{H5-H4}$ = 4.4 Hz, $^{4}J_{H5-H3}$ = 1.5 Hz, 1H, H5), 4.30 (d, $^{2}J_{H6a-H6b}$ = 7.4 Hz, 1H, H6a), 3.87 (ddd, $^{3}J_{H4-F3}$ = 26.8 Hz, $^{3}J_{H4-H3}$ = 4.8 Hz, $^{3}J_{H4-H5}$ = 3.5 Hz, 1H, H4), 3.70 (dd $^{2}J_{H6b-H6a}$ = 7.4 Hz, $^{3}J_{H6b-H5}$ = 5.4 Hz, 1H) ppm; ^{13}C {^{1}H} NMR (126 MHz, $CDCl_3$) δ 137.25 (1C, CAr), 128.81 (1C, CAr), 128.44 (1C, CAr), 128.02 (1C, CAr), 98.42 (d, $^{2}J_{C1-F2}$ = 25.8 Hz, 1C, C1), 86.48 (dd, $^{1}J_{C2-F2}$ = 182.1 Hz, $^{2}J_{C2-F3}$ = 27.6 Hz, 1C, C2), 85.24 (dd, $^{1}J_{C3-F3}$ = 184.7 Hz, $^{2}J_{C3-F2}$ = 31.7 Hz, 1C, C3), 72.65 (d, $^{3}J_{C5-F3}$ = 1.0 Hz, 1C, C5), 71.30 (1C, *C*H_2Ph), 70.82 (d, $^{2}J_{C4-F3}$ = 16.6 Hz, 1C, C4), 64.54 (d, $^{4}J_{C6-F3}$ = 3.8 Hz, 1C, C6) ppm; ^{19}F NMR (470 MHz, $CDCl_3$) δ -194.30 (ddd, $^{2}J_{F2-H2}$ = 43.8 Hz, $^{3}J_{F2-F3}$ = 15.8 Hz, $^{3}J_{F2-H3}$ = 9.4 Hz, 1F, F2), -206.42 (dddd, $^{2}J_{F3-H3}$ = 47.1 Hz, $^{3}J_{F3-H4}$ = 27.0 Hz, $^{3}J_{F3-F2}$ = 15.8 Hz, $^{3}J_{F3-H2}$ = 11.2 Hz, 1F, F3) ppm; HRMS calcd for $C_{13}H_{18}F_2NO_3^+$ $[M + NH_4]^+$ 274.1249 found 274.1251.

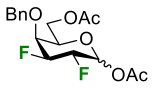

42

1,6-Di-*O*-acetyl-4-*O*-benzyl-2,3-dideoxy-2,3-difluoro-α/β-D-galactopyranoside (42). To a stirred solution of 1,6-anhydro-4-*O*-benzyl-2,3-dideoxy-2,3-difluoro-β-D-galactopyranoside **41** (77.3 mg, 0.3017 mmol) in Ac_2O (2 mL) at 0 °C were added two drops of TESOTf (≈ 20−30 µL, cat) under a N_2 atmosphere. After 1 h, a saturated aqueous $NaHCO_3$ solution was added, and the mixture was stirred for 30 min. The reaction mixture was extracted with EtOAc. The combined organic phases were washed with a saturated aqueous $NaHCO_3$ solution and brine. The organic phase was dried over $MgSO_4$, filtered, and concentrated under reduced pressure. The crude residue was purified by flash column chromatography (silica gel,

EtOAc/hexanes, 1:4 → 3:7) to give **42** as an anomeric mixture (α/β, 10:1) as an amorphous white solid (97.5 mg, 0.2721 mmol, 90% yield). R_f = 0.26 (silica, EtOAc/hexanes 1:3); $[\alpha]_D^{25}$ = +60.42 (c 0.3, $CHCl_3$); IR (ATR, diamond crystal) *v* 3032, 2953, 2922, 1744, 1371, 1221, 1078 cm^{-1}; only the α anomer has been attributed in ^{1}H NMR and ^{13}C NMR; ^{1}H NMR (500 MHz, $CDCl_3$) δ 7.39 – 7.29 (m, 6H, H_{Ar}), 6.44 (t, $^{3}J_{H1–H2}$ = 4.3 Hz, $^{3}J_{H1–F2}$ = 4.3 Hz, 1H, H1), 5.13 (dddd, $^{2}J_{H2–F2}$ = 49.7 Hz, $^{3}J_{H2–F3}$ = 11.5 Hz, $^{3}J_{H2–H3}$ = 9.5 Hz, $^{3}J_{H2–H1}$ = 4.2 Hz, 1H, H2), 5.01 (dddd, $^{2}J_{H3–F3}$ = 49.2 Hz, $^{3}J_{H3–F2}$ = 12.0 Hz, $^{3}J_{H3–H2}$ = 9.6 Hz, $^{3}J_{H3–H4}$ = 3.3 Hz, 1H, H3), 4.92 (d, $^{2}J_{C\underline{H}aHbPh–CHa\underline{H}bPh}$ = 11.3 Hz, 1H, CH_2Ph), 4.59 (d, $^{2}J_{CHa\underline{H}bPh–C\underline{H}aHbPh}$ = 11.3 Hz, 1H, CH_2Ph), 4.19 (ddd, $^{2}J_{H6a–H6b}$ = 10.4 Hz, $^{3}J_{H6a–H5}$ = 5.8 Hz, 1.3 Hz, 1H, H6a), 4.10 (ddd, $^{3}J_{H4–F3}$ = 6.9 Hz, $^{3}J_{H4–H3}$ = 4.3 Hz, $^{3}J_{H4–H5}$ = 1.3 Hz, 1H, H4), 4.08 (dd, $^{2}J_{H6b–H6a}$ = 10.6 Hz, $^{3}J_{H6b–H5}$ = 6.3 Hz, 1H, H6b), 4.05 (ddt, $^{3}J_{H5–H6b}$ = 6.3 Hz, $^{3}J_{H5–H6a}$ = 5.8 Hz, $^{3}J_{H5–H4}$ = 1.5 Hz, 1.5 Hz, 1H, H5), 2.14 (s, 3H, COCH_3), 1.98 (s, 3H, COCH_3) ppm; ^{13}C {^{1}H} NMR (126 MHz, $CDCl_3$) δ 170.52 (1C, *C*OCH_3), 168.87 (1C, *C*OCH_3), 137.16 (1C, Ar), 128.69 (2C, Ar), 128.67 (2C, Ar), 128.40 (1C, Ar), 90.05 (dd, $^{1}J_{C3-F3}$ = 190.0 Hz, $^{2}J_{C3-F2}$ = 17.6 Hz, 1C, C3), 89.58 (dd, $^{2}J_{C1-F2}$ = 22.4 Hz, $^{3}J_{C1-F3}$ = 9.6 Hz, 1C, C1), 85.73 (dd, $^{1}J_{C2-F2}$ = 190.1 Hz, $^{2}J_{C2-F3}$ = 18.9 Hz, 1C, C2), 75.16 (d, $^{4}J_{\underline{C}H2Ph-F3}$ = 4.4 Hz, 1C, *C*H_2Ph), 74.25 (dd, $^{2}J_{C4-F3}$ = 16.0 Hz, $^{3}J_{C4-F2}$ = 7.7 Hz, 1C, C4), 70.04 (dd, $^{3}J_{C5-F3}$ = 6.8 Hz, $^{4}J_{C5-F2}$ = 0.9 Hz, 1C, C5), 62.17 (d, $^{4}J_{C6-F3}$ = 2.5 Hz, 1C, C6), 20.98 (1C, CO*C*H_3), 20.86 (1C, CO*C*H_3) ppm; ^{19}F NMR (470 MHz, $CDCl_3$) δ -199.19 (dtd, $^{2}J_{F3–H3}$ = 48.9 Hz, $^{3}J_{F3–F2}$ = 13.1 Hz, $^{3}J_{F3–H2}$ = 13.0 Hz, $^{3}J_{F3–H4}$ = 5.8 Hz, F3β), -203.02 (ddddd, $^{2}J_{F3–H3}$ = 49.5 Hz, $^{3}J_{F3–F2}$ = 12.3 Hz, $^{3}J_{F3–H2}$ = 12.2 Hz, $^{3}J_{F3–H4}$ = 6.4 Hz, 5.0 Hz, F3α), -209.84 (dtt, $^{2}J_{F2–H2}$ = 52.1 Hz, $^{3}J_{F2–H2}$ = $^{3}J_{F2–F2}$ = 13.8 Hz, 3.9 Hz, 3.9 Hz, F2β), -211.60 (dtd, $^{2}J_{F2–H2}$ = 49.7 Hz, $^{3}J_{F2–F3}$ = $^{3}J_{F2–H3}$ = 12.6 Hz, $^{3}J_{F2–H1}$ = 4.0 Hz, F2α) ppm; HRMS calcd for $C_{17}H_{24}F_2NO_6^-$ $[M + NH_4]^+$ 376.1566 found 376.1572.

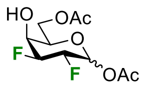

43

1,6-Di-*O*-acetyl-2,3-dideoxy-2,3-difluoro-α/β-D-galactopyranoside (43). To a stirred solution of 1,6-di-*O*-acetyl-4-*O*-benzyl-2,3-dideoxy-2,3-difluoro-α/β-D-galactopyranoside **42** (58.4 mg, 0.1630 mmol) in anhydrous CH_2Cl_2 (1.6 mL, 0.1 M) at 0 °C was added $TiCl_4$ (71 µL, 0.6520 mmol, 4 equiv.) under a N_2 atmosphere. After 45 min, water was added. The reaction mixture was extracted with EtOAc. The combined organic phases were washed with brine. The combined organic phases were dried over $MgSO_4$, filtered, and concentrated under reduced pressure. The crude residue was purified by flash column chromatography (silica gel, EtOAc/hexanes, 1:4 → 1:1) to give **43** as an anomeric mixture (α/β 16:1) as

an amorphous white solid (36.5 mg, 0.1361 mmol, 83% yield). R_f = 0.31 (silica, EtOAc/hexanes 1:1); $[\alpha]_D^{25}$ = +129.58 (c 0.5, $CHCl_3$); IR (ATR, diamond crystal) v 3485, 2972, 2926, 1757, 1724, 1377, 1211, 1034 cm^{-1}; only the α anomer has been attributed in ^1H NMR and ^{13}C NMR, and ^{19}F NMR; ^1H NMR (500 MHz, $CDCl_3$) δ 6.45 (dd, $^3J_{H1–H2}$ = 4.2 Hz, $^3J_{H1–F2}$ = 3.9 Hz, 1H, H1), 5.06 (dddd, $^2J_{H2–F2}$ = 49.7 Hz, $^3J_{H2–F3}$ = 12.4 Hz, $^3J_{H2–H3}$ = 9.3 Hz, $^3J_{H2–H1}$ = 4.2 Hz, 1H, H2), 4.93 (dddd, $^2J_{H3–F3}$ = 49.8 Hz, $^3J_{H3–F2}$ = 12.6 Hz, $^3J_{H3–H2}$ = 9.3 Hz, $^3J_{H3–H4}$ = 3.5 Hz, 1H, H3), 4.38 (dd, $^2J_{H6a–H6b}$ = 11.6 Hz, $^3J_{H6a–H5}$ = 6.4 Hz, 1H, H6a), 4.30 (dqd, $^3J_{H4–F3}$ = 7.0 Hz, $^3J_{H4–H3}$ = $^3J_{H4–H5}$ = $^3J_{H4–OH}$ = 3.4 Hz, 1.3 Hz, 1H, H4), 4.24 (ddd, $^2J_{H6b–H6a}$ = 11.6 Hz, $^3J_{H6b–H5}$ = 6.4 Hz, $^4J_{H6b–H4}$ = 1.3 Hz, 1H, H6b), 4.09 (t, $^3J_{H5–H6a}$ = $^3J_{H5–H6b}$ = 6.4 Hz, 1H, H5), 2.49 (dt, $^3J_{OH–H4}$ = 3.0 Hz, 1.6, 1.6 Hz, 1H, OH4), 2.16 (s, 3H, COCH_3), 2.09 (s, 3H, COCH_3). ppm; ^{13}C {^1H} NMR (126 MHz, $CDCl_3$) δ 171.05 (1C, COCH$_3$), 168.85 (1C, COCH$_3$), 89.46 (dd, $^2J_{C1–F2}$ = 22.5 Hz, $^3J_{C1–F3}$ = 9.7 Hz, 1C, C1), 88.76 (dd, $^1J_{C3–F3}$ = 186.0 Hz, $^2J_{C3–F2}$ = 18.1 Hz, 1C, C2), 85.23 (dd, $^2J_{C2–F2}$ = 190.1 Hz, $^2J_{C2–F3}$ = 19.0 Hz, 1C, C2), 69.61 (dd, $^3J_{C5–F3}$ = 5.6 Hz, $^4J_{C5–F2}$ = 0.8 Hz, 1C, C5), 68.07 (dd, $^2J_{C4–F3}$ = 17.6 Hz, $^3J_{C4–F2}$ = 7.7 Hz, 1C, C4), 62.13 (d, $^4J_{C6–F3}$ = 2.8 Hz, 1C, C6), 20.98 (1C, COCH$_3$), 20.94 (1C, COCH$_3$) ppm; ^{19}F NMR (470 MHz, $CDCl_3$) δ -204.22 (ddddd, $^2J_{F3–H3}$ = 50.1 Hz, $^3J_{F3–F2}$ = 14.0 Hz, $^3J_{F3–H2}$ = 12.6 Hz, $^3J_{F3–H4}$ = 7.0 Hz, 5.6 Hz, 1F, F3), -211.87 (dddd, $^2J_{F2–H2}$ = 49.8 Hz, $^3J_{F2–F3}$ = 14.0 Hz, $^3J_{F2–H3}$ = 12.7 Hz, $^3J_{F2–H1}$ = 3.8 Hz, 1F, F2). ppm; HRMS calcd for $C_{10}H_{18}F_2NO_6^+$ $[M + NH_4]^+$ 286.1097 found 286.1101.

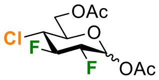

45

1,6-Di-*O*-acetyl-4-chloro-2,3,4-trideoxy-2,3-difluoro-α/β-D-glucopyranose (45). To a stirred solution of 1,6-di-*O*-acetyl-2,3-dideoxy-2,3-difluoro-α/β-D-galactopyranoside **43** (53.9 mg, 0.2010 mmol, 1.0 equiv.) in CH_2Cl_2 (2.0 mL) at 0 °C, were added pyridine (48.8 µL, 0.6029 mmol, 3 equiv.) and Tf_2O (50.8 µL, 0.3014 mmol, 1.5 equiv.). The mixture was stirred at room temperature for 1.5 h and then quenched with water. The mixture was extracted with CH_2Cl_2, and the combined organic phases were successively washed with an aqueous 1 M HCl solution and brine. The organic solution was dried over $MgSO_4$, filtered, and concentrated under reduced pressure. To a stirred solution of the crude triflate in CH_2Cl_2 (2.0 mL) was added TBACl (168 mg, 0.6029 mmol, 3 equiv.). The mixture was stirred at room temperature for 27 h and then quenched with water. The mixture was extracted with CH_2Cl_2, and the combined organic phases were washed with a brine. The organic solution was dried over $MgSO_4$, filtered, and concentrated under reduced pressure. The crude residue was purified by flash column chromatography (silica gel,

acetone/toluene, 1:4 → 3:7) to give an anomeric mixture (α/β, 14.7:1) of **45** as a thick colorless oil (49.9 mg, 0.1741 mmol, 87% over 2 steps). R_f = 0.37 (silica, EtOAc/hexanes 3:7); $[\alpha]_D^{25}$ = +80.95 (c 0.5, $CHCl_3$); IR (ATR, diamond crystal) *ν* 2961, 2924, 2854, 1763, 1744, 1454, 1211, 1074, 802 cm^{-1}; only the α anomer has been attributed in ^{1}H NMR, ^{13}C NMR, and ^{19}F NMR; ^{1}H NMR (500 MHz, $CDCl_3$) δ 6.44 (dd, $^3J_{H1–H2}$ = 4.1 Hz, $^4J_{H1–F3}$ = 3.9 Hz, 1H, H1), 4.89 (dddd, $^2J_{H3–F3}$ = 51.8 Hz, $^3J_{H3–F2}$ = 12.3 Hz, $^3J_{H3–H4}$ = 9.5 Hz, $^3J_{H3–H2}$ = 8.8 Hz, 1H, H3), 4.68 (dddd, $^2J_{H2–F2}$ = 48.9 Hz, $^3J_{H2–F3}$ = 12.1 Hz, $^3J_{H2–H3}$ = 8.7 Hz, $^3J_{H2–H1}$ = 4.1 Hz, 1H, H2), 4.43 – 4.34 (m, 2H, H6a, H6b), 4.07 (dt, $^3J_{H5–H4}$ = 10.8 Hz, $^3J_{H5–H6a}$ = $^3J_{H5–H6b}$ = 3.7 Hz, 1H, H5), 3.97 (ddd, $^3J_{H4–F3}$ = 10.9 Hz, $^3J_{H4–H5}$ = 10.8 Hz, $^3J_{H4–H3}$ = 9.5 Hz, 1H, H4), 2.20 (s, 3H, COC*H*$_3$), 2.11 (s, 3H, COC*H*$_3$) ppm; ^{13}C {^{1}H} NMR (126 MHz, $CDCl_3$) δ 170.47 (1C, *C*OCH$_3$), 168.50 (1C, *C*OCH$_3$), 91.35 (dd, $^1J_{C3–F3}$ = 190.8 Hz, $^2J_{C3–F2}$ = 20.0 Hz, 1C, C3), 88.62 (dd, $^2J_{C1–F2}$ = 22.4 Hz, $^3J_{C1–F3}$ = 9.7 Hz, 1C, C1), 86.74 (dd, $^1J_{C2–F2}$ = 196.0 Hz, $^2J_{C2–F2}$ = 18.7 Hz, 1C, C2), 71.60 (d, $^3J_{C5–F3}$ = 5.1 Hz, 1C, C5), 61.96 (s, 1C, C6), 53.72 (dd, $^2J_{C4–F3}$ = 18.6 Hz, $^3J_{C4–F2}$ = 6.8 Hz, 1C, C4), 20.91 (1C, CO*C*H$_3$), 20.85 (1C, CO*C*H$_3$) ppm; ^{19}F NMR (470 MHz, $CDCl_3$) δ -189.44 (dq, *J* = 50.4, 13.8, 13.8, 10.4 Hz, 1F, F3β), -193.40 (ddddd, $^2J_{F3–H3}$ = 51.9 Hz, $^3J_{F3–F2}$ = 14.8 Hz, $^3J_{F3–H2}$ = 12.9 Hz, $^3J_{F3–H4}$ = 10.9 Hz, $^4J_{F3–H1}$ = 3.9 Hz, 1F, F3α), -199.62 (dtd, *J* = 51.2, 14.5, 14.5, 3.2 Hz, 1F, F2β), -200.84 (dt, $^2J_{F2–H2}$ = 48.9 Hz, $^3J_{F2–F3}$ = 14.7 Hz, $^3J_{F2–H3}$ = 12.3 Hz, 1F, F2α) ppm; HRMS calcd for $C_{10}H_{17}ClF_2NO_5^+$ $[M + NH_4]^+$ 304.0758 found 304.0760.

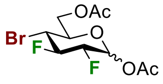

46

1,6-Di-*O*-acetyl-4-bromo-2,3,4-trideoxy-2,3-difluoro-α/β-D-glucopyranose (46). To a stirred solution of 1,6-di-*O*-acetyl-2,3-dideoxy-2,3-difluoro-α/β-D-galactopyranoside **43** (49.6 mg, 0.1849 mmol, 1.0 equiv.) in CH_2Cl_2 (1.8 mL) at 0 °C, were added pyridine (44.9 μL, 0.5548 mmol, 3 equiv.) and Tf_2O (46.7 μL, 0.2774 mmol, 1.5 equiv.). The mixture was stirred at room temperature for 1.5 h and then quenched with water. The mixture was extracted with CH_2Cl_2, and the combined organic phases were successively washed with an aqueous 1 M HCl solution and brine. The organic solution was dried over $MgSO_4$, filtered, and concentrated under reduced pressure. To a stirred solution of the crude triflate in CH_2Cl_2 (1.8 mL) was added TBABr (179 mg, 0.5448 mmol, 3 equiv.). The mixture was stirred at room temperature for 27 h and then quenched with water. The mixture was extracted with CH_2Cl_2, and the combined organic phases were washed with a brine. The organic solution was dried over $MgSO_4$, filtered, and concentrated under reduced pressure. The crude residue was purified by flash column chromatography (silica gel,

acetone/toluene, 1:3) to give an anomeric mixture (α/β, 14.7:1) of **46** as a thick colorless oil (54.5 mg, 0.1646 mmol, 89% over 2 steps). R_f = 0.42 (silica, EtOAc/hexanes 3:7); $[\alpha]_D^{25}$ = +70.45 (c 1.0, $CHCl_3$); IR (ATR, diamond crystal) *ν* 2961, 2924, 2854, 1761, 1742, 1454, 1213, 1072 cm^{-1}; only the α anomer has been attributed in ^1H NMR, ^{13}C NMR, and ^{19}F NMR; ^1H NMR (500 MHz, $CDCl_3$) δ 6.46 (t, $^3J_{H1–H2}$ = 3.9 Hz, $^4J_{H1–F3}$ = 3.9 Hz, 1H, H1), 4.96 (dddd, $^2J_{H3–F3}$ = 51.2 Hz, $^3J_{H3–F2}$ = 12.0 Hz, $^3J_{H3–H4}$ = 10.1 Hz, $^3J_{H3–H2}$ = 8.8 Hz, 1H, H3), 4.67 (dddd, $^2J_{H2–F2}$ = 48.9 Hz, $^3J_{H2–F3}$ = 12.8 Hz, $^3J_{H2–H3}$ = 8.7 Hz, $^3J_{H2–H1}$ = 4.0 Hz, 1H, H2), 4.51 – 4.36 (m, 2H, H6a, H6b), 4.16 (dt, $^3J_{H5–H4}$ = 11.0 Hz, $^3J_{H5–H6a}$ = $^3J_{H5–H6b}$ = 3.1 Hz, 1H, H5), 3.98 (q, $^3J_{H4–H5}$ = 10.5 Hz, $^3J_{H4–H3}$ = $^3J_{H4–F3}$ = 10.3 Hz, 1H, H4), 2.20 (s, 3H, COCH_3), 2.11 (s, 3H, COCH_3) ppm; ^{13}C {^1H} NMR (126 MHz, $CDCl_3$) δ 170.44 (1C, *C*OCH$_3$), 168.51 (1C, *C*OCH$_3$), 91.28 (dd, $^1J_{C3–F3}$ = 190.3 Hz, $^2J_{C3–F2}$ = 20.1 Hz, 1C, C3), 88.70 (dd, $^2J_{C1–F2}$ = 22.4 Hz, $^3J_{C1–F3}$ = 9.6 Hz, 1C, C1), 86.71 (dd, $^1J_{C2–F2}$ = 196.9 Hz, $^2J_{C2–F3}$ = 19.1 Hz, 1C, C2), 71.64 (d, $^3J_{C5–F3}$ = 5.1 Hz, 1C, C5), 62.68 (1C, C6), 43.59 (dd, $^2J_{C4–F3}$ = 18.9 Hz, $^3J_{C3–F2}$ = 6.3 Hz, 1C, C4), 20.92 (1C, CO*C*H$_3$), 20.85 (1C, CO*C*H$_3$) ppm; ^{19}F NMR (470 MHz, $CDCl_3$) δ -185.80 (dtd, *J* = 49.5, 14.4, 14.0, 10.4 Hz, 1F, F3β), -189.15 (dqd, $^2J_{F3–H3}$ = 51.3 Hz, $^3J_{F3–F2}$ = 15.0 Hz, $^3J_{F3–H2}$ = 12.8 Hz, $^3J_{F3–H4}$ = 10.4 Hz, $^4J_{F3–H1}$ = 3.9 Hz, 1F, F3α), -198.53 (dtd, *J* = 51.1, 14.4, 14.4, 3.1 Hz, F2β), -199.46 (ddd, $^2J_{F2–H2}$ = 49.0 Hz, $^3J_{F2–F3}$ = 15.0 Hz, $^3J_{F2–H3}$ = 11.9 Hz, 1F, F2α) ppm; HRMS calcd for $C_{10}H_{17}BrF_2NO_5^+$ $[M + NH_4]^+$ 348.0253 found 348.0249.

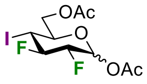

47

1,6-Di-*O*-acetyl-2,3,4-trideoxy-2,3-difluoro-4-iodo-α/β-D-glucopyranose (47). To a stirred solution of 1,6-di-*O*-acetyl-2,3-dideoxy-2,3-difluoro-α/β-D-galactopyranoside **43** (48.0 mg, 0.1790 mmol, 1.0 equiv.) in CH_2Cl_2 (1.8 mL) at 0 °C, were added pyridine (43.4 μL, 0.5369 mmol, 3 equiv.) and Tf_2O (45.2 μL, 0.2684 mmol, 1.5 equiv.). The mixture was stirred at room temperature for 1.5 h and then quenched with water. The mixture was extracted with CH_2Cl_2, and the combined organic phases were successively washed with an aqueous 1 M HCl solution and brine. The organic solution was dried over $MgSO_4$, filtered, and concentrated under reduced pressure. To a stirred solution of the crude triflate in CH_2Cl_2 (1.8 mL) was added TBAI (198 mg, 0.5369 mmol, 3 equiv.). The mixture was stirred at room temperature for 27 h and then quenched with water. The mixture was extracted with CH_2Cl_2, and the combined organic phases were washed with a brine. The organic solution was dried over $MgSO_4$, filtered, and concentrated under reduced pressure. The crude residue was purified by flash column chromatography (silica gel, acetone/toluene, 1:3) to give an anomeric mixture (α/β, 12.2:1) of **47** as a thick colorless oil (57.9 mg,

0.1531 mmol, 86% over 2 steps). R_f = 0.43 (silica, EtOAc/hexanes 3:7); $[\alpha]_D^{25}$ = +65.7 (c 0.5, $CHCl_3$); IR (ATR, diamond crystal) ν 2957, 2924, 2854, 1761, 1742, 1454, 1213, 1070 cm^{-1}; only the α anomer has been attributed in ^{1}H NMR, ^{13}C NMR, and ^{19}F NMR; ^{1}H NMR (500 MHz, $CDCl_3$) δ 6.47 (dd, $^3J_{H1-H2}$ = 4.1 Hz, $^4J_{H1-F3}$ = 3.9 Hz, 1H, H1), 4.97 (dddd, $^2J_{H3-F3}$ = 50.5 Hz, $^3J_{H3-F2}$ = 11.4 Hz, $^3J_{H3-H4}$ = 10.6 Hz, $^3J_{H3-H2}$ = 8.4 Hz, 1H, H3), 4.65 (dddd, $^2J_{H2-F2}$ = 49.1 Hz, $^3J_{H2-F3}$ = 12.6 Hz, $^3J_{H2-H3}$ = 8.7 Hz, $^3J_{H2-H1}$ = 4.1 Hz, 1H, H2), 4.54 – 4.45 (m, 2H, H6a, H6b), 4.22 (dt, $^3J_{H5-H4}$ = 11.4 Hz, $^3J_{H5-H6a}$ = $^3J_{H5-H6b}$ = 3.1 Hz, 1H, H5), 4.01 (ddd, $^3J_{H4-H5}$ = 11.4 Hz, $^3J_{H4-H3}$ = 10.6 Hz, $^3J_{H4-F3}$ = 9.1 Hz, 1H), 2.19 (s, 3H, $COCH_3$), 2.10 (s, 3H, $COCH_3$) ppm; ^{13}C {^{1}H} NMR (126 MHz, $CDCl_3$) δ 170.43 (s, 1C, $COCH_3$), 168.58 (s, 1C, $COCH_3$), 92.00 (dd, $^1J_{C3-F3}$ = 189.2 Hz, $^2J_{C3-F2}$ = 20.0 Hz, 1C, C3), 88.92 (dd, $^2J_{C1-F2}$ = 22.5 Hz, $^3J_{C1-F3}$ = 9.9 Hz, 1C, C1), 86.27 (dd, $^1J_{C2-F2}$ = 197.5 Hz, $^2J_{C2-F3}$ = 19.3 Hz, 1C, C2), 72.45 (d, $^3J_{C5-F3}$ = 5.6 Hz, 1C, C5), 64.09 (s, 1C, C6), 20.93 (s, 1C, $COCH_3$), 20.84 (s, 1C, $COCH_3$), 20.63 (dd, $^2J_{C4-F3}$ = 18.7 Hz, $^3J_{C4-F2}$ = 5.3 Hz, 1C, C4) ppm; ^{19}F NMR (470 MHz, $CDCl_3$) δ -179.59 (dtd, J = 48.4, 14.7, 14.6, 7.7 Hz, 1F, F3β), -181.90 (ddddd, $^2J_{F3-H3}$ = 50.3 Hz, $^3J_{F3-F2}$ = 16.3 Hz, $^3J_{F3-H2}$ = 12.6 Hz, $^3J_{F3-H4}$ = 9.0 Hz, $^4J_{F3-H1}$ = 3.9 Hz, 1F, F3α), -197.48 (dddd, J = 51.4, 16.2, 13.4, 3.2 Hz, 1F, F2β), -197.95 (ddd, $^2J_{F2-H2}$ = 49.1 Hz, $^3J_{F2-F3}$ = 16.1 Hz, $^3J_{F2-H3}$ = 11.4 Hz, 1F, F2α) ppm; HRMS calcd for $C_{10}H_{17}F_2INO_5^+$ $[M + NH_4]^+$ 396.0114 found 396.0101.

II. ^{19}F NMR spectra prediction

^{19}F NMR chemical shifts were predicted using two NMR prediction tools: the MestReNova software, the Machine Learning Predictions of ^{19}F NMR chemical shifts online tool developed by Li et al.,[2] and nmrshiftdb2 online tool.[3] The chemical shifts were predicted for the α anomers only and compared to the experimental values.

As shown on **Figure S1**, most Δδ values are negative, which mean that the chemical shift predicted with MestReNova are more downfield than the experimental signals. The few exceptions are the F4 signal of galactose analogue **12** and the F3 signals of iodinated galactose **15**, iodinated mannose **34**, and the three talose analogues **31**, **33**, and **35**. The carbohydrate series with the most accurate prediction is the mannose analogues **28**, **30**, **32**, and **34** with an average of the absolute Δδ values of 4.23 ppm. The smallest difference is for the F2 signal of chlorinated talose **31** (Δδ = 0.06 ppm), and the biggest difference is for the F4 of trifluorinated talose **29** (Δδ = -45.98 ppm).

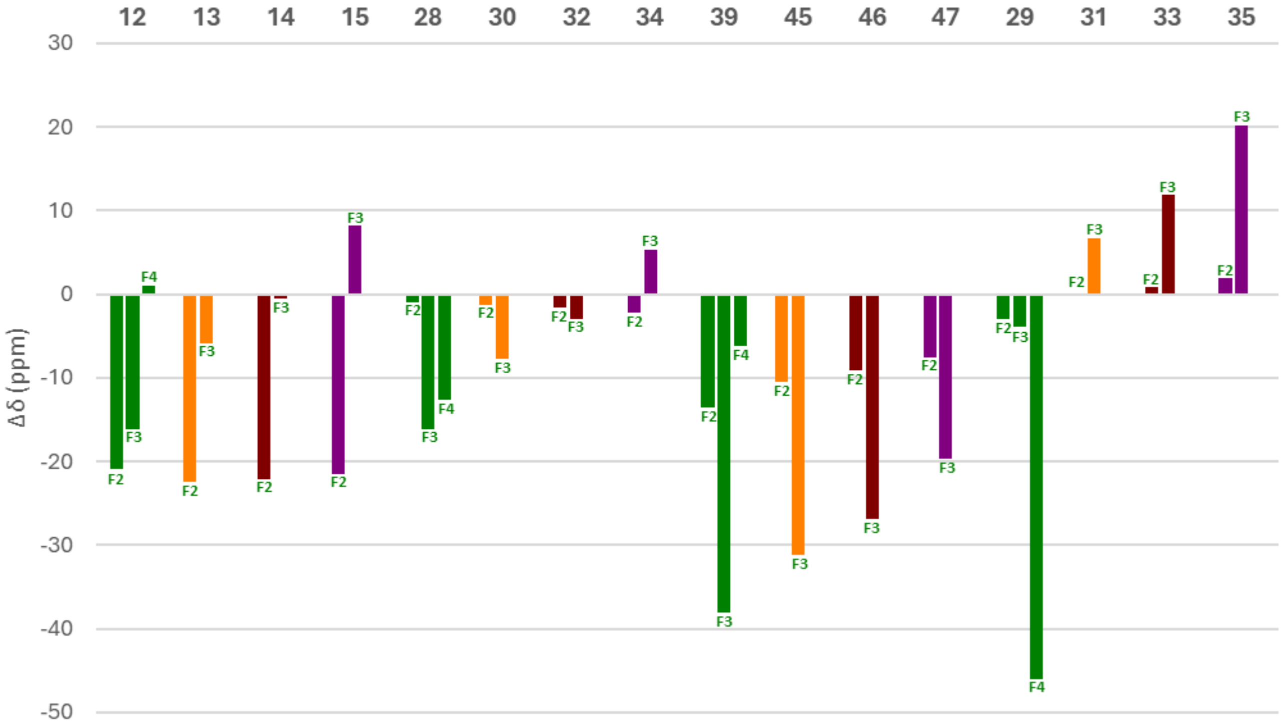

Figure S1. Graphically depicted ^{19}F NMR chemical shift differences (Δδ, ppm) between experimental spectra and predicted spectra (using MestReNova's prediction tool) for α anomers of trihalogenated carbohydrates.

Figure S2 shows that 21 of the 36 chemical shifts predicted with the tool developed by Li et al. are more downfield, and 15 are more upfield. All F2 signals are predicted more downfield than the experimental values, except for the F2 of iodinated glucose **47**. Apart from the trifluorinated compound **12**, **28**, and **29**, the F3 shifts are predicted more upfield than the experimental values. The F4 signals of trifluorinated compounds are predicted more downfield, except for glucose **39**. The carbohydrate series with the most accurate prediction is the glucose analogues **39**, and **45**–**47** with an average of the absolute Δδ values of 4.66 ppm. The smallest difference is for the F2 signal of iodinated glucose **47** (Δδ = 0.05 ppm), and the biggest difference is for the F3 of iodinated glucose **47** (Δδ = 17.00 ppm).

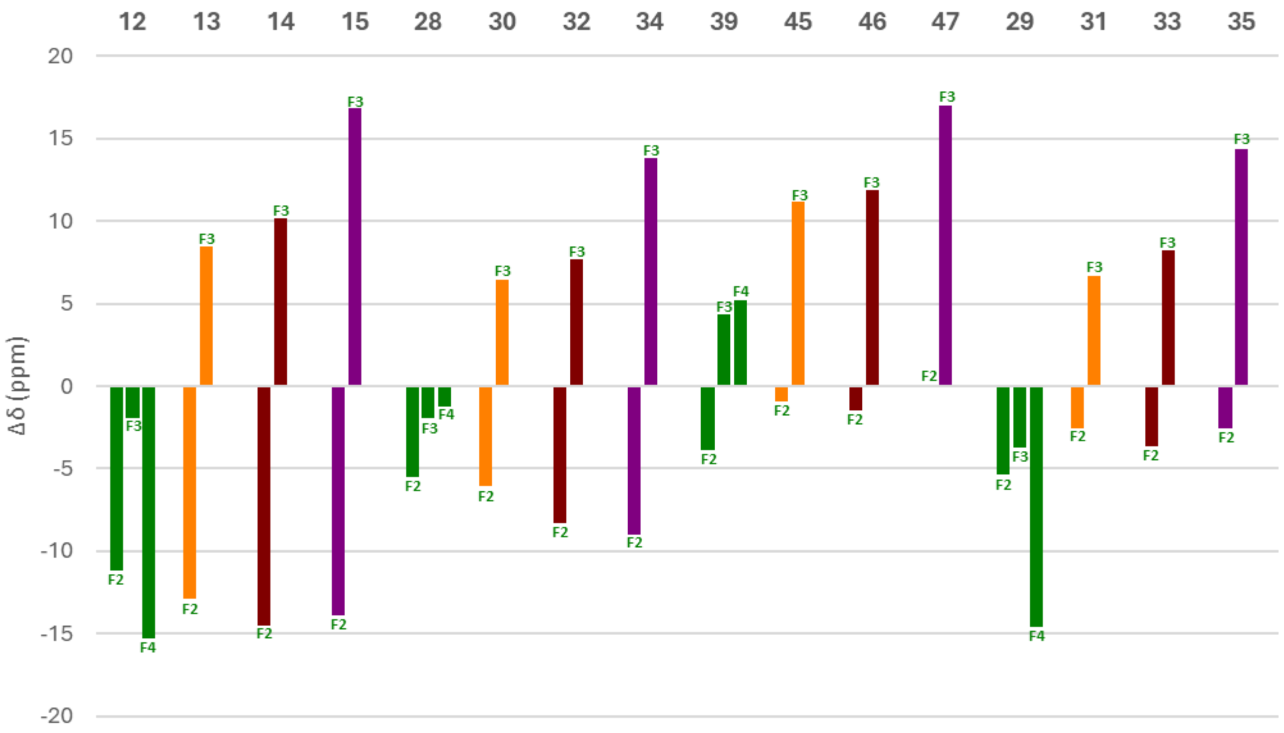

Figure S2. Graphically depicted ^{19}F NMR chemical shift differences (Δδ, ppm) between experimental spectra and predicted spectra (using the prediction tool developed by Li et al.[2]) for α anomers of trihalogenated carbohydrates.

As shown on **Figure S3**, most Δδ values are negative, which mean that the chemical shift predicted with nmrshiftdb2 are more downfield than the experimental signals. The few exceptions are the F3 signal of chlorinated glucose analogue **45**, and of all the brominated and iodinated analogues. The carbohydrate series with the most accurate prediction is the glucose analogues **39**, **45–47** with an average of the absolute Δδ values of 4.72 ppm. The smallest difference is for the F3 signal of chlorinated glucose **45** (Δδ = 0.43 ppm), and the biggest difference is for the F4 of trifluorinated galactose **12** (Δδ = -26.72 ppm).

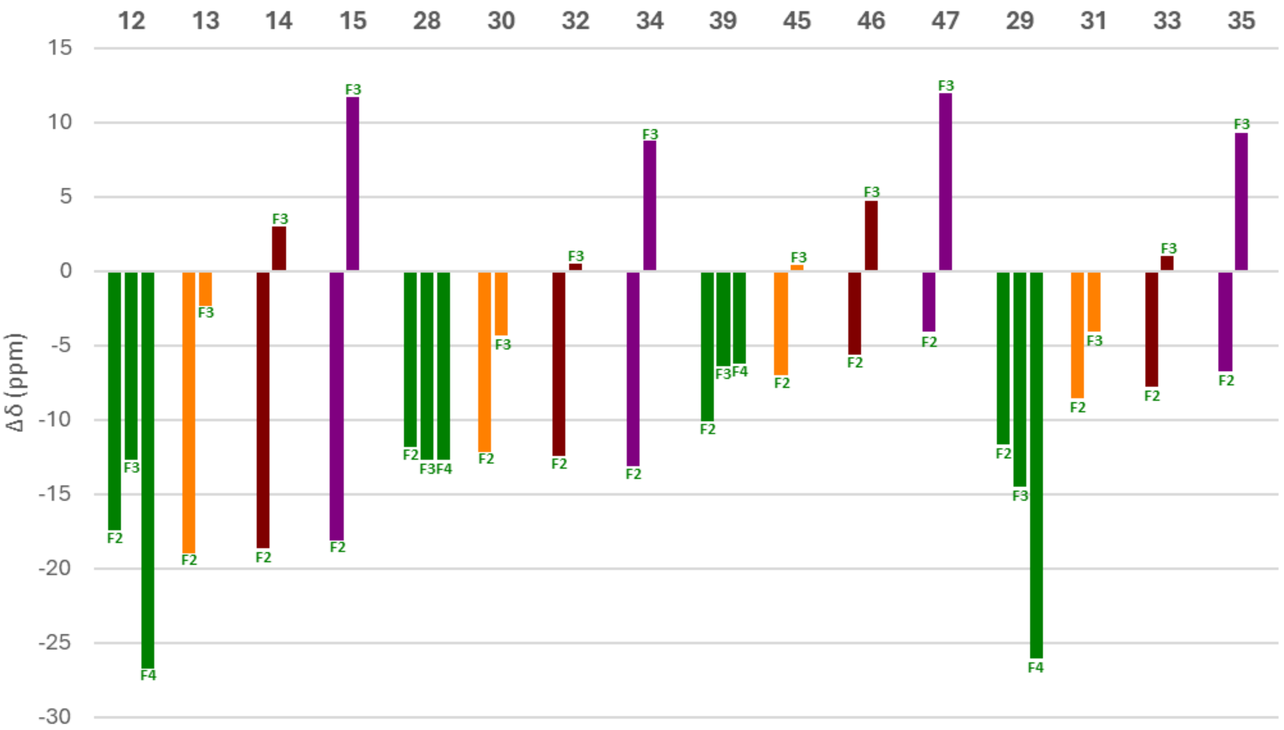

Figure S3. Graphically depicted ^{19}F NMR chemical shift differences (Δδ, ppm) between experimental spectra and predicted spectra (using the nmrshift2 tool[3]) for α anomers of trihalogenated carbohydrates.

III. Crystal structure determination

Table S1. Crystal data and structure refinement for compound **34**

Empirical formula	$C_{10}H_{13}F_2IO_5$
Formula weight	378.10
Temperature [K]	150
Crystal system	orthorhombic
Space group (number)	$P2_12_12_1$ (19)
a [Å]	8.0206(2)
b [Å]	9.2560(3)
c [Å]	18.2337(6)
α [°]	90
β [°]	90
γ [°]	90
Volume [Å^3]	1353.65(7)
Z	4
ρ_{calc} [gcm^{-3}]	1.855
μ [mm^{-1}]	12.984
F(000)	736
Crystal size [mm^3]	0.24×0.19×0.17
Crystal colour	clear light colourless
Crystal shape	block
Radiation	Ga K_α (λ=1.34139 Å)
2θ range [°]	8.44 to 126.96 (0.75 Å)
Index ranges	$-10 \leq h \leq 10$<br>$-11 \leq k \leq 12$<br>$-23 \leq l \leq 24$
Reflections collected	40449
Independent reflections	3372<br>R_{int} = 0.0443<br>R_{sigma} = 0.0178
Completeness to θ = 53.594°	99.6 %
Data / Restraints / Parameters	3372/0/165
Absorption correction T_{min}/T_{max} (method)	0.1786/0.3145 (multi-scan)
Goodness-of-fit on F^2	1.083
Final R indexes [$I \geq 2\sigma(I)$]	R_1 = 0.0215<br>wR_2 = 0.0537
Final R indexes [all data]	R_1 = 0.0218<br>wR_2 = 0.0539
Largest peak/hole [eÅ^{-3}]	0.34/−1.12
Flack X parameter	−0.006(3)

IV. NMR spectra of compounds

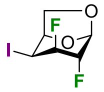

11

^{1}H NMR Spectrum
($CDCl_3$, 500 MHz)

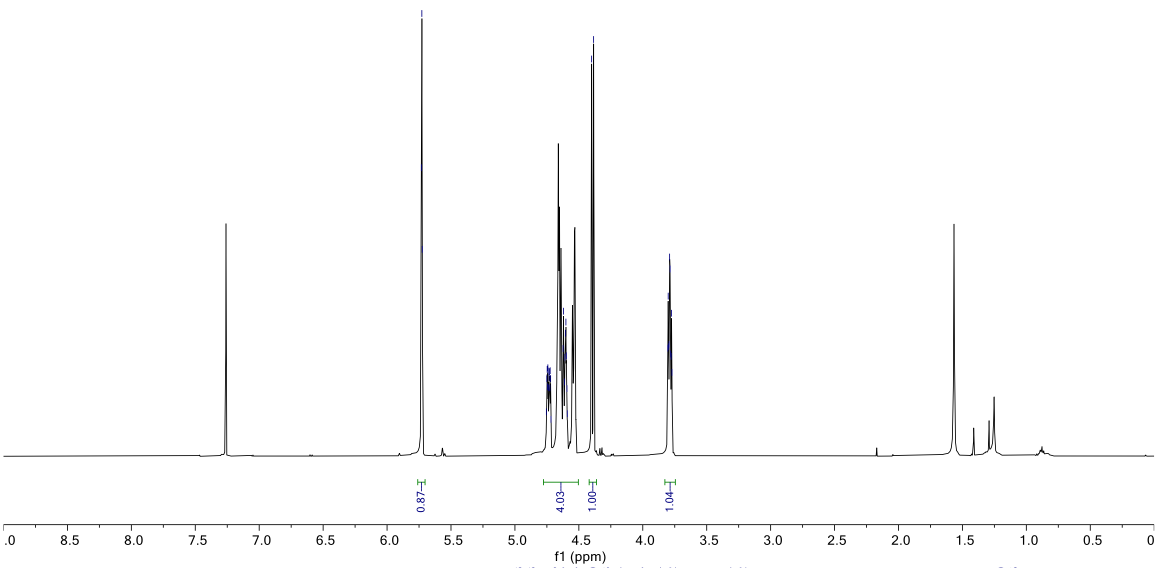

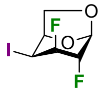

11

^{13}C NMR Spectrum
($CDCl_3$, 126 MHz)

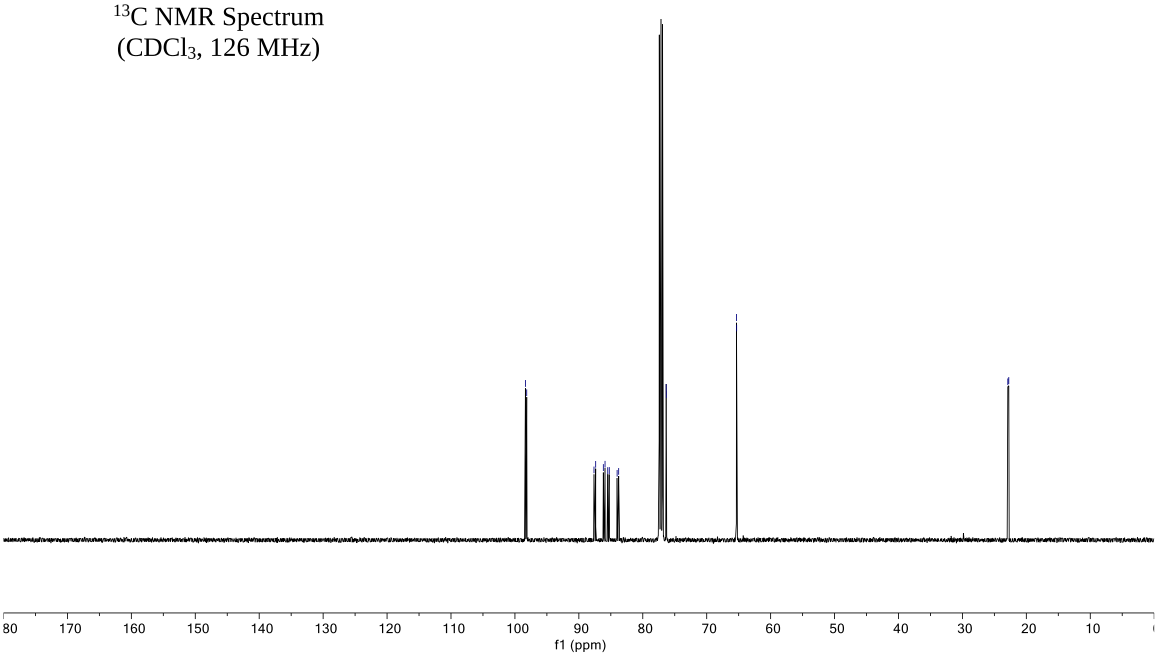

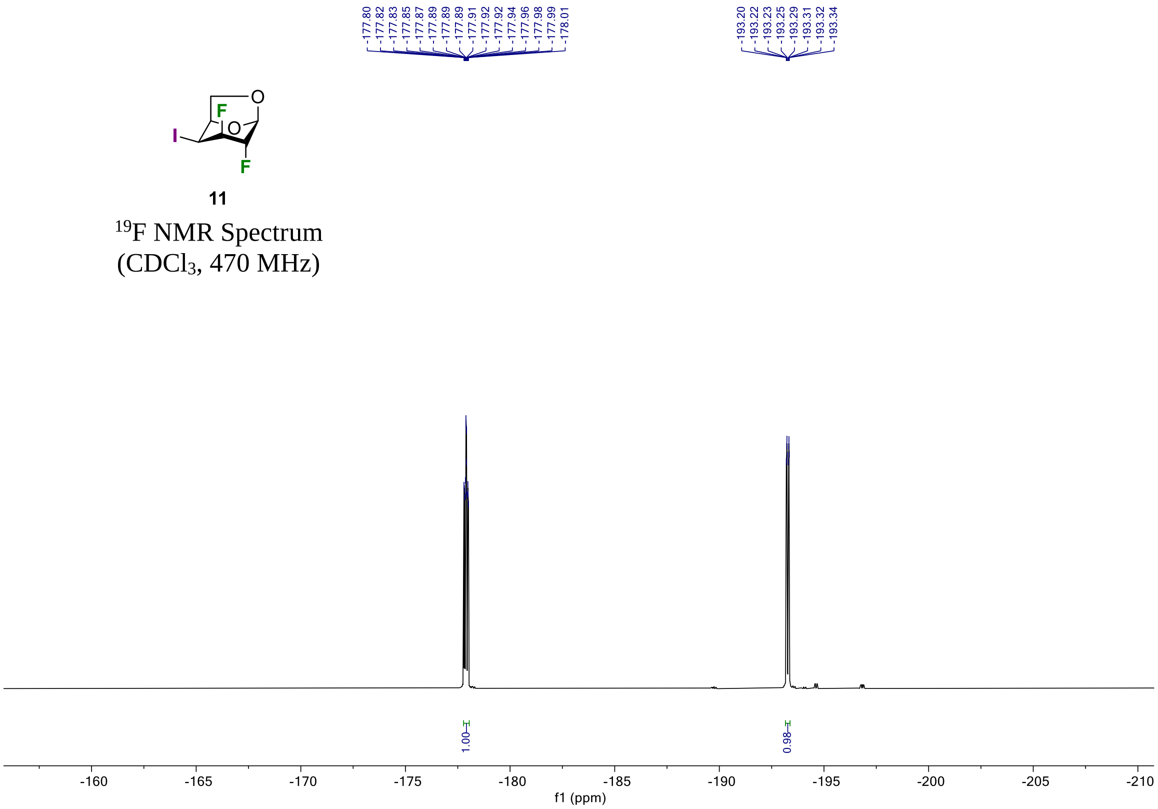
-177.80
-177.82
-177.83
-177.85
-177.87
-177.89
-177.89
-177.89
-177.91
-177.92
-177.92
-177.94
-177.96
-177.98
-177.99
-178.01
-193.20
-193.22
-193.23
-193.25
-193.29
-193.31
-193.32
-193.34
11
^{19}F NMR Spectrum
($CDCl_3$, 470 MHz)
1.00
0.98
-160 -165 -170 -175 -180 -185 -190 -195 -200 -205 -210
f1 (ppm)

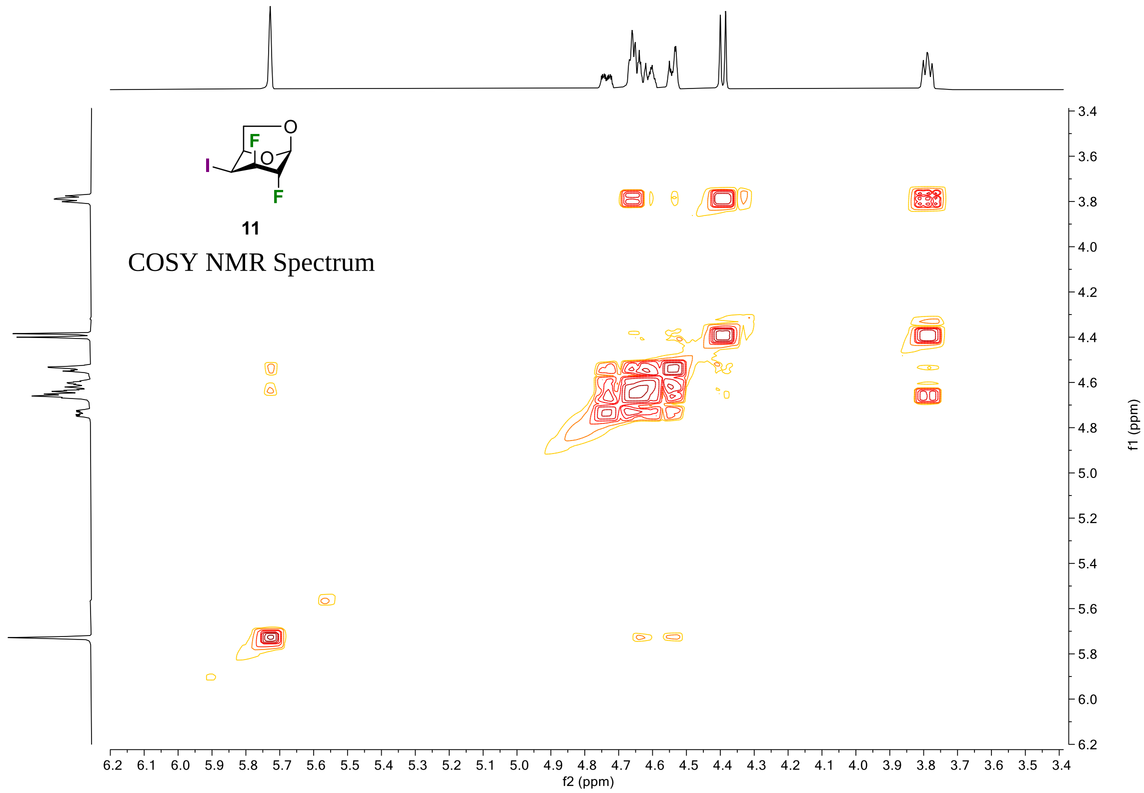
11
COSY NMR Spectrum
f2 (ppm)
f1 (ppm)

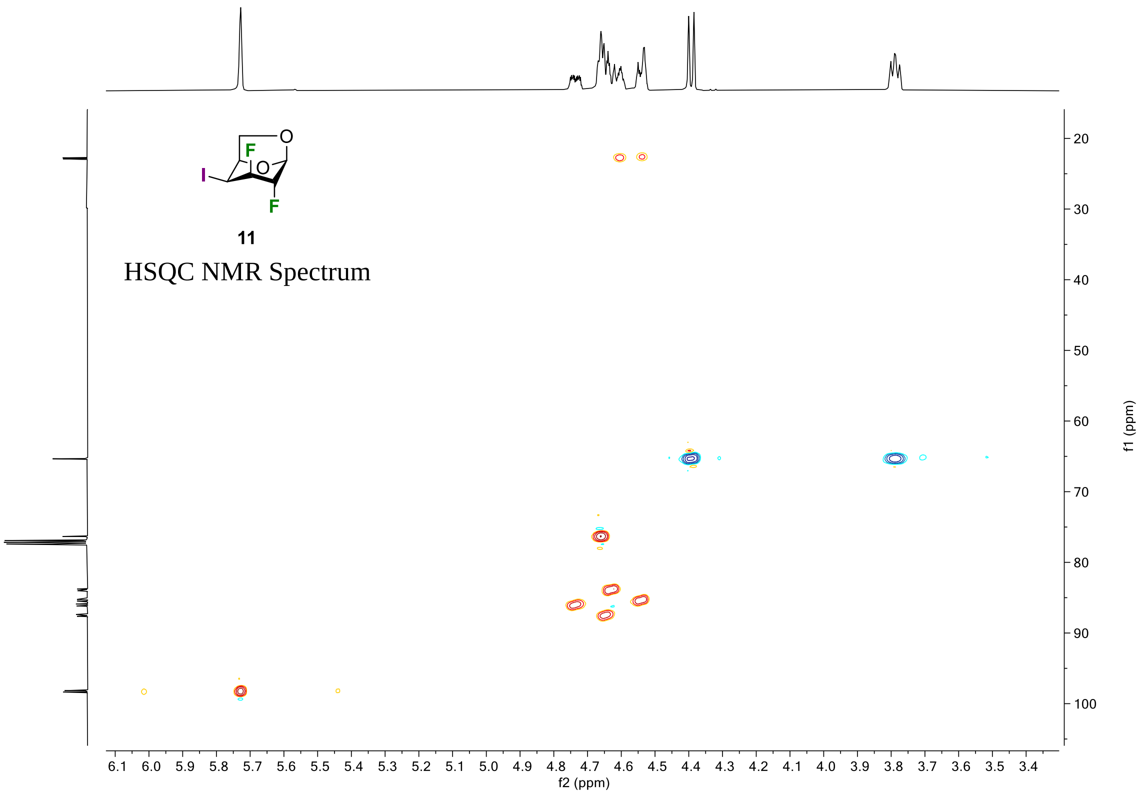

11

HSQC NMR Spectrum

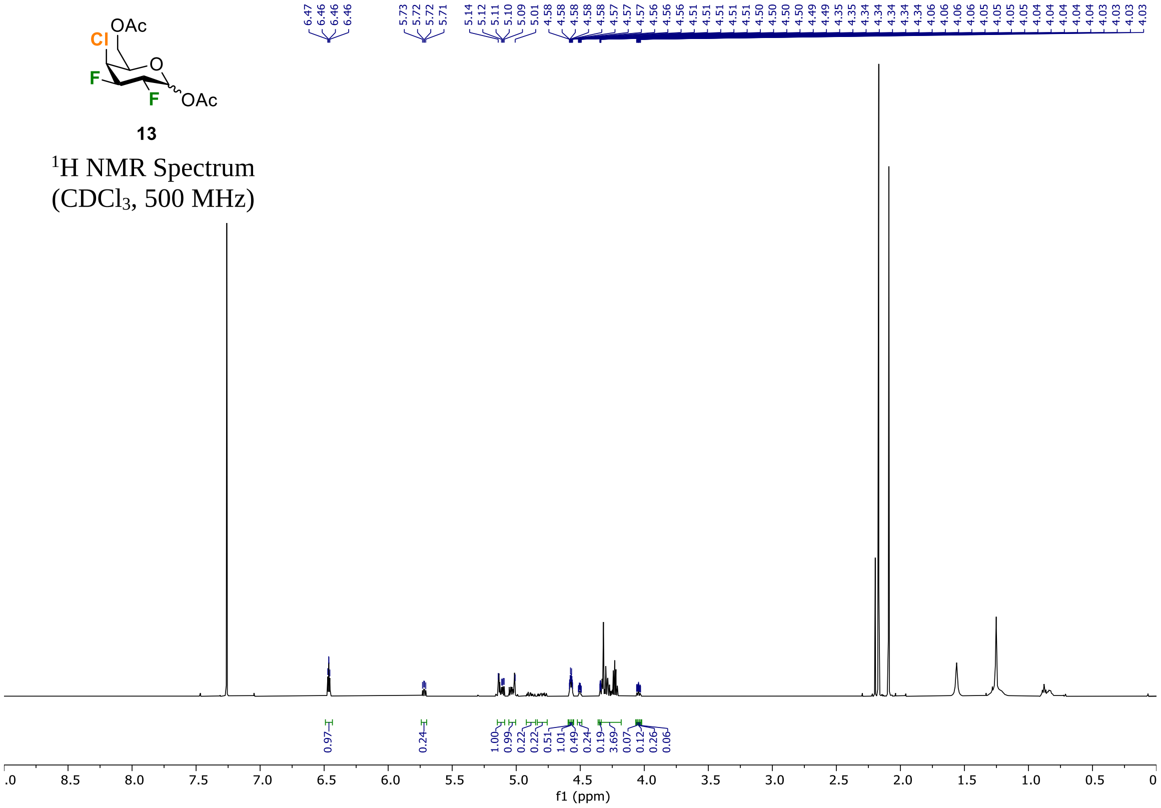

13

^{1}H NMR Spectrum
($CDCl_3$, 500 MHz)

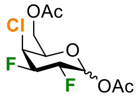

13

^{13}C NMR Spectrum
($CDCl_3$, 126 MHz)

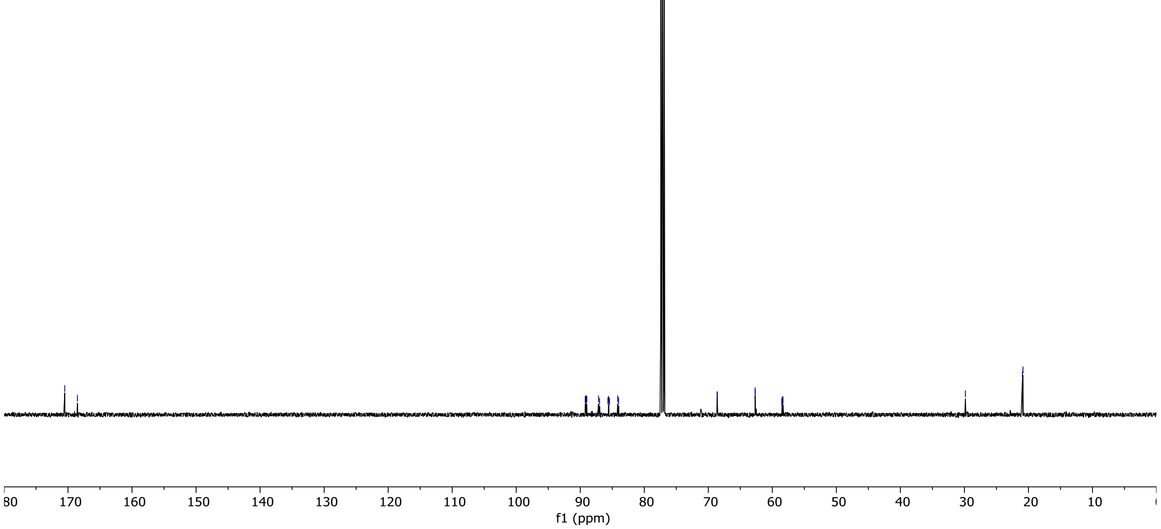

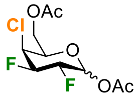

13

^{19}F NMR Spectrum
($CDCl_3$, 470 MHz)

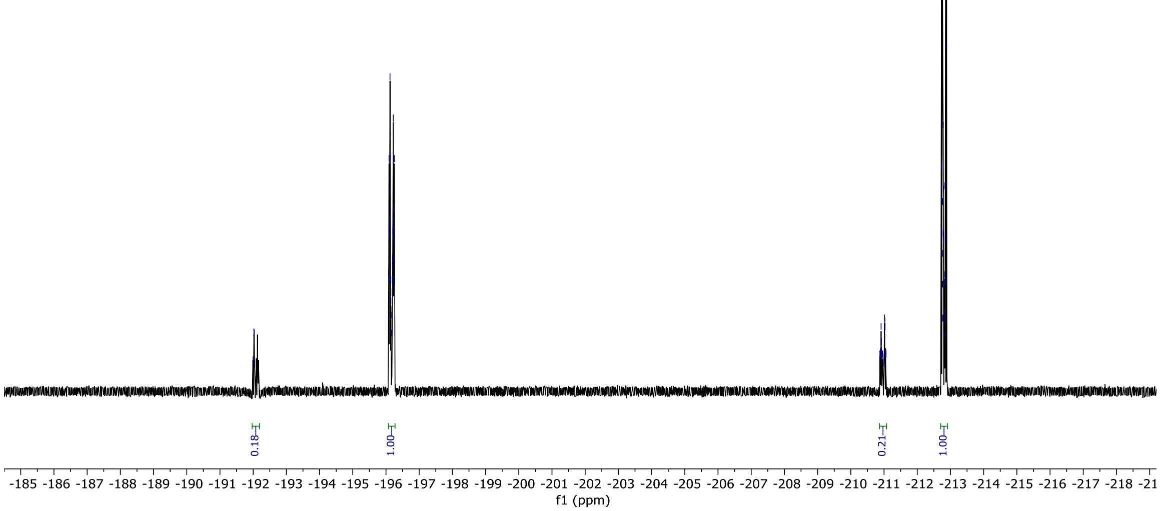

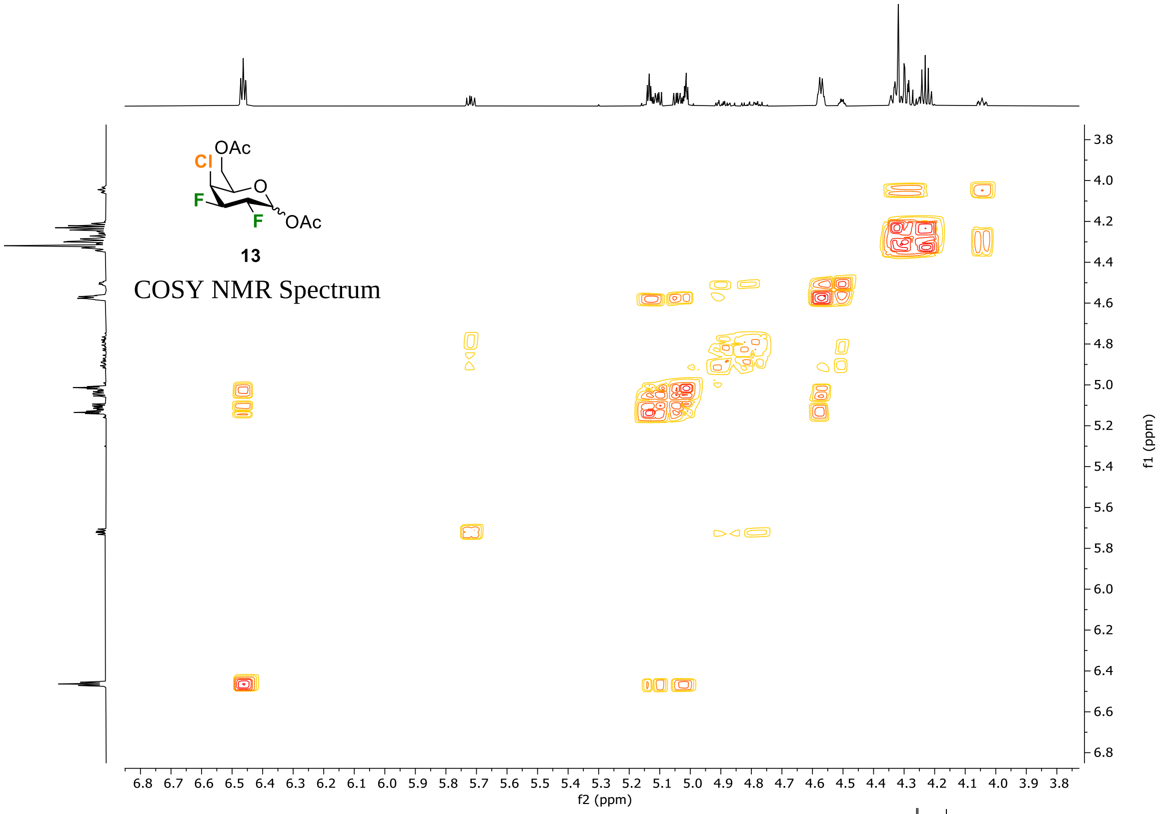

OAc
Cl
F
F
OAc
13
COSY NMR Spectrum
f2 (ppm)
f1 (ppm)

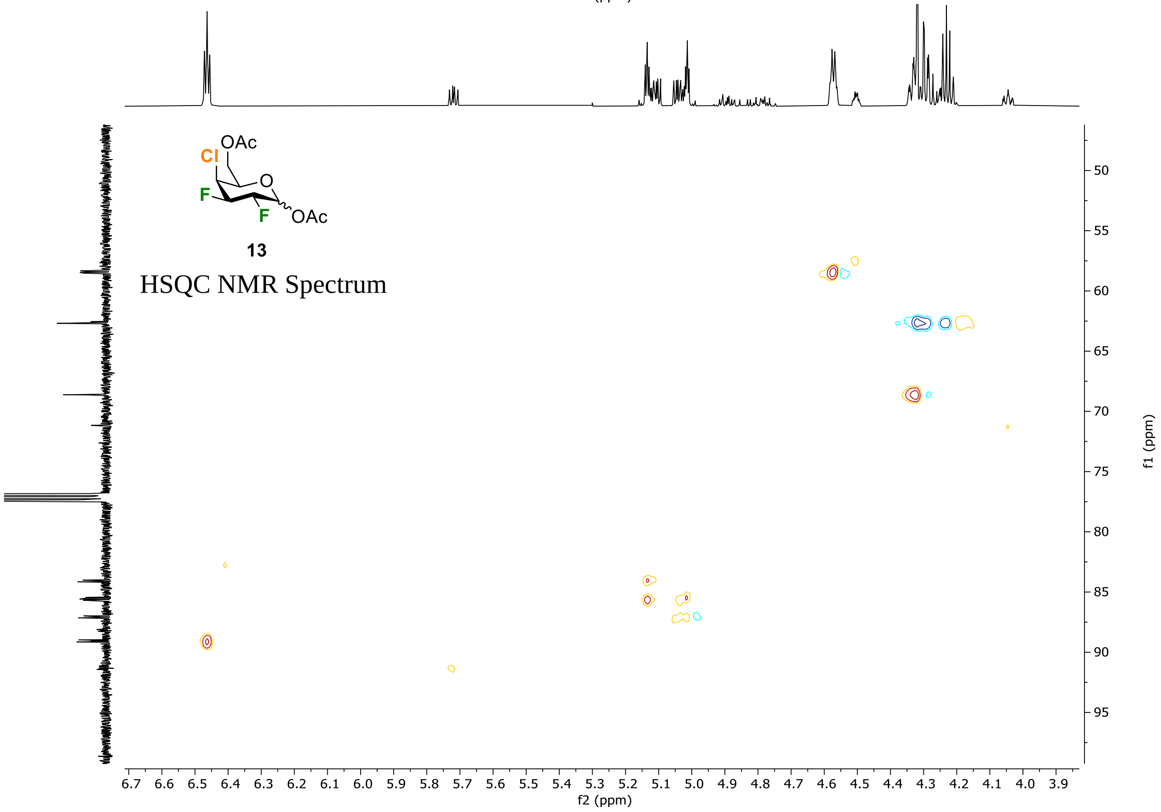

OAc
Cl
F
F
OAc
13
HSQC NMR Spectrum
f2 (ppm)
f1 (ppm)

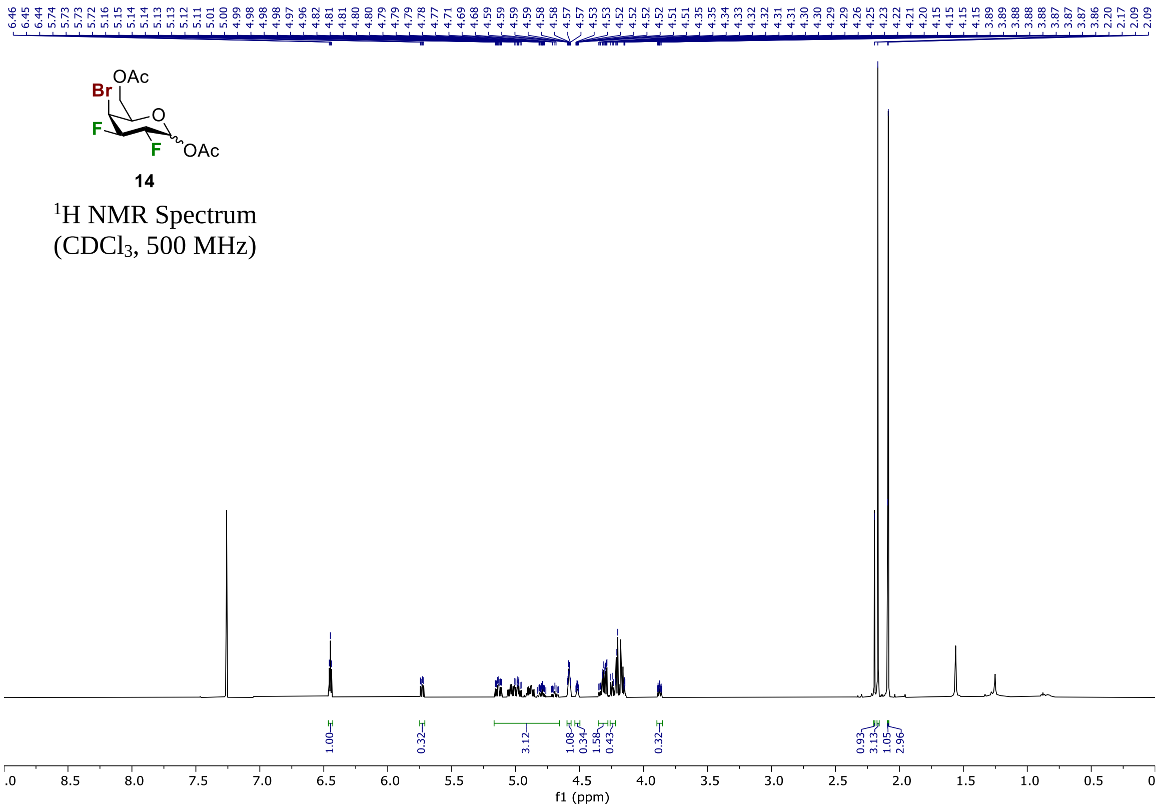

^{1}H NMR Spectrum
($CDCl_3$, 500 MHz)

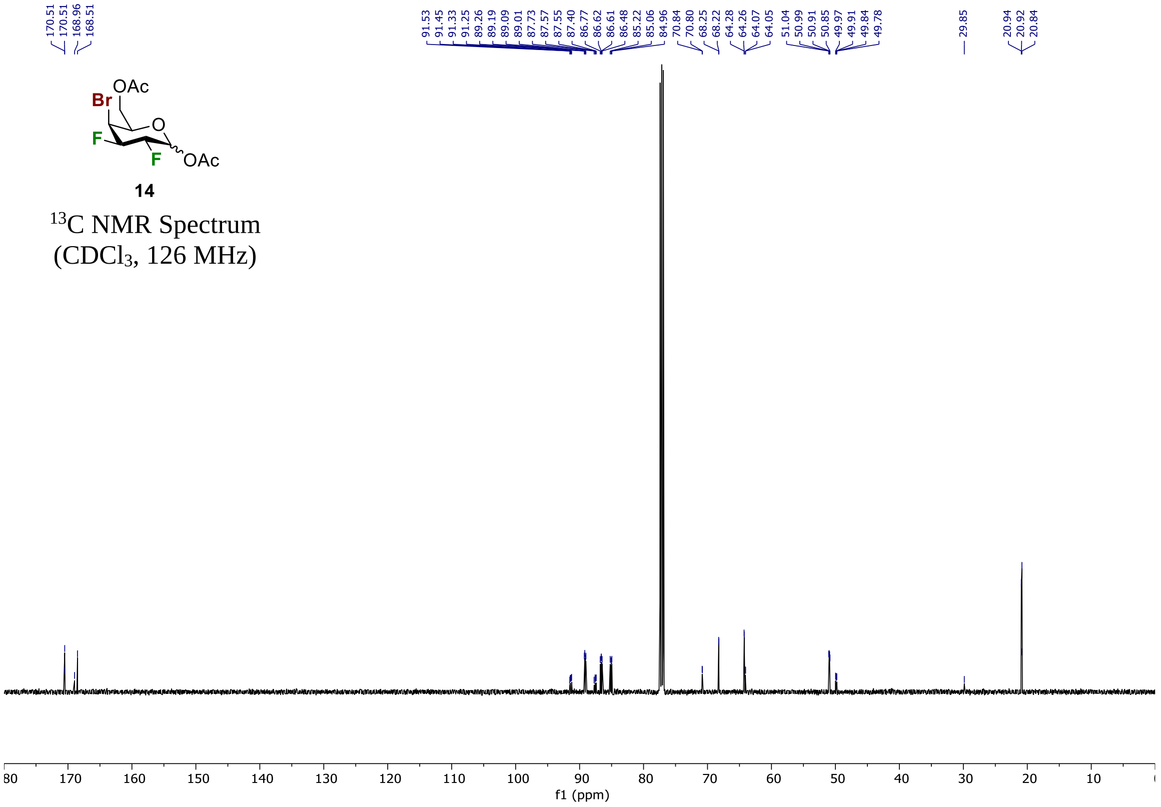

^{13}C NMR Spectrum
($CDCl_3$, 126 MHz)

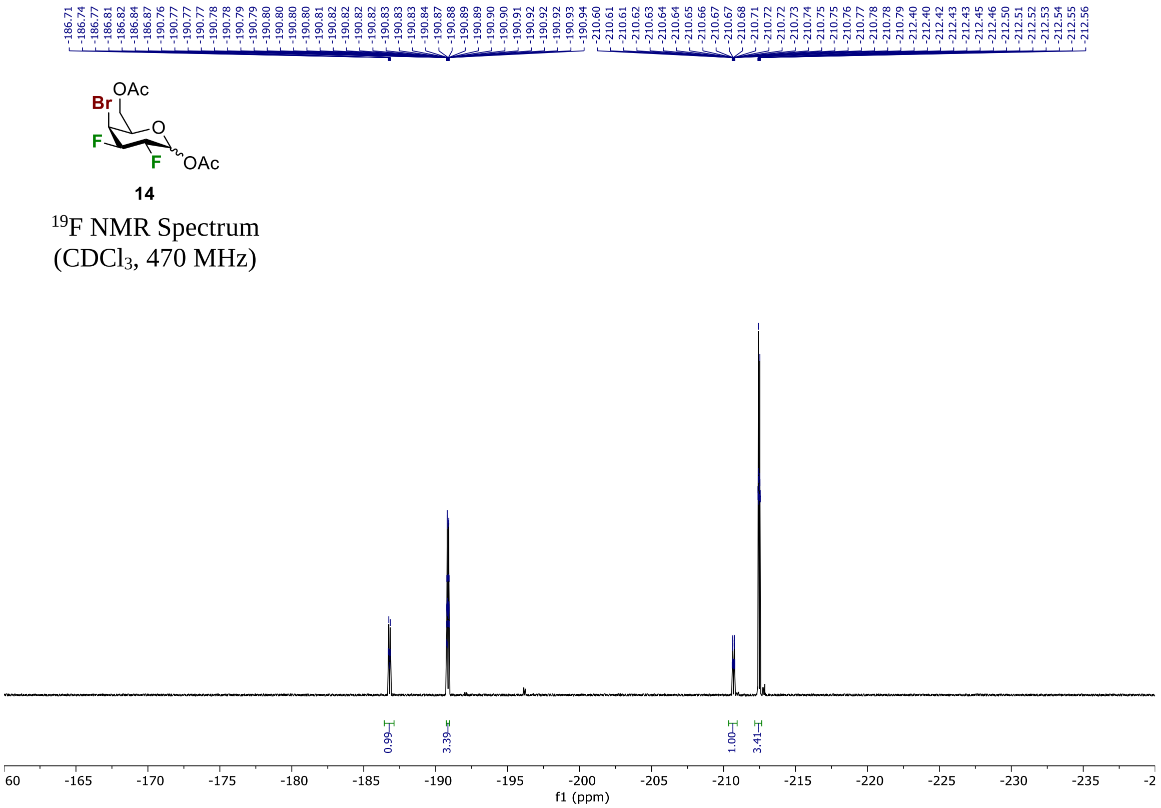
OAc
Br
O
F
F
OAc
14
19F NMR Spectrum
(CDCl3, 470 MHz)
0.99
3.39
1.00
3.41
f1 (ppm)

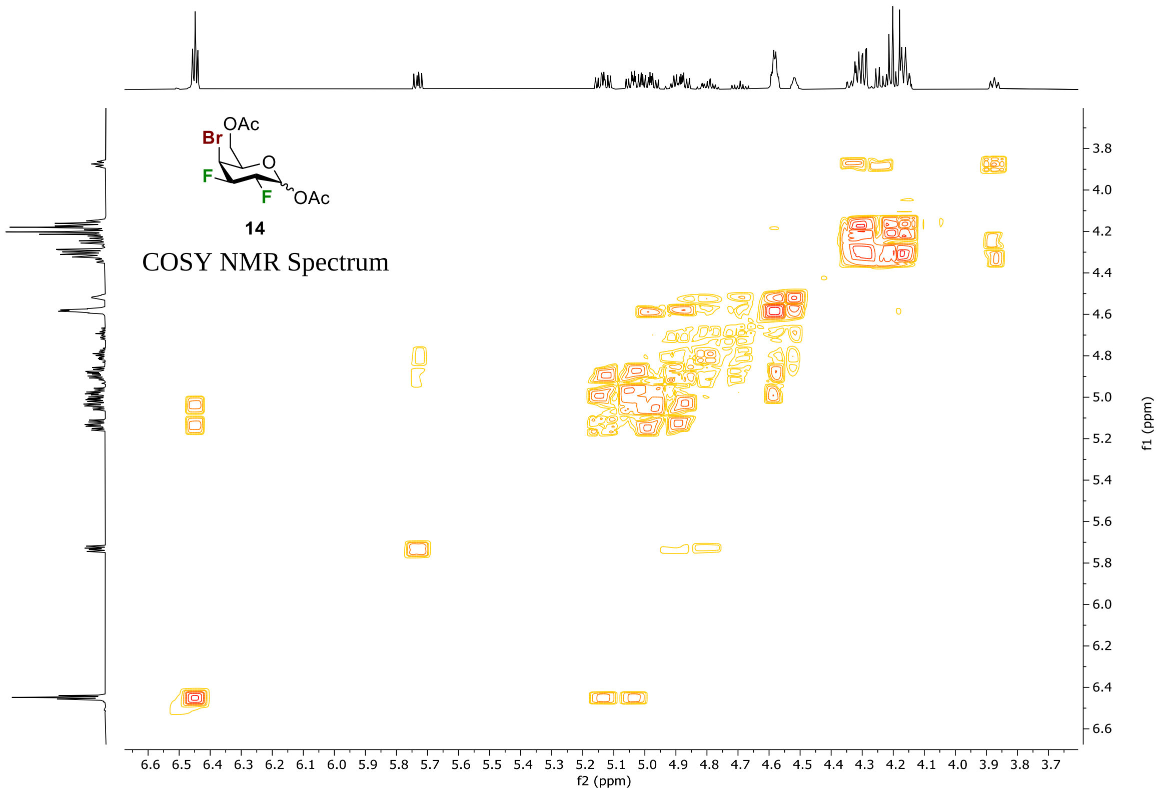
OAc
Br
O
F
F
OAc
14
COSY NMR Spectrum
f2 (ppm)
f1 (ppm)

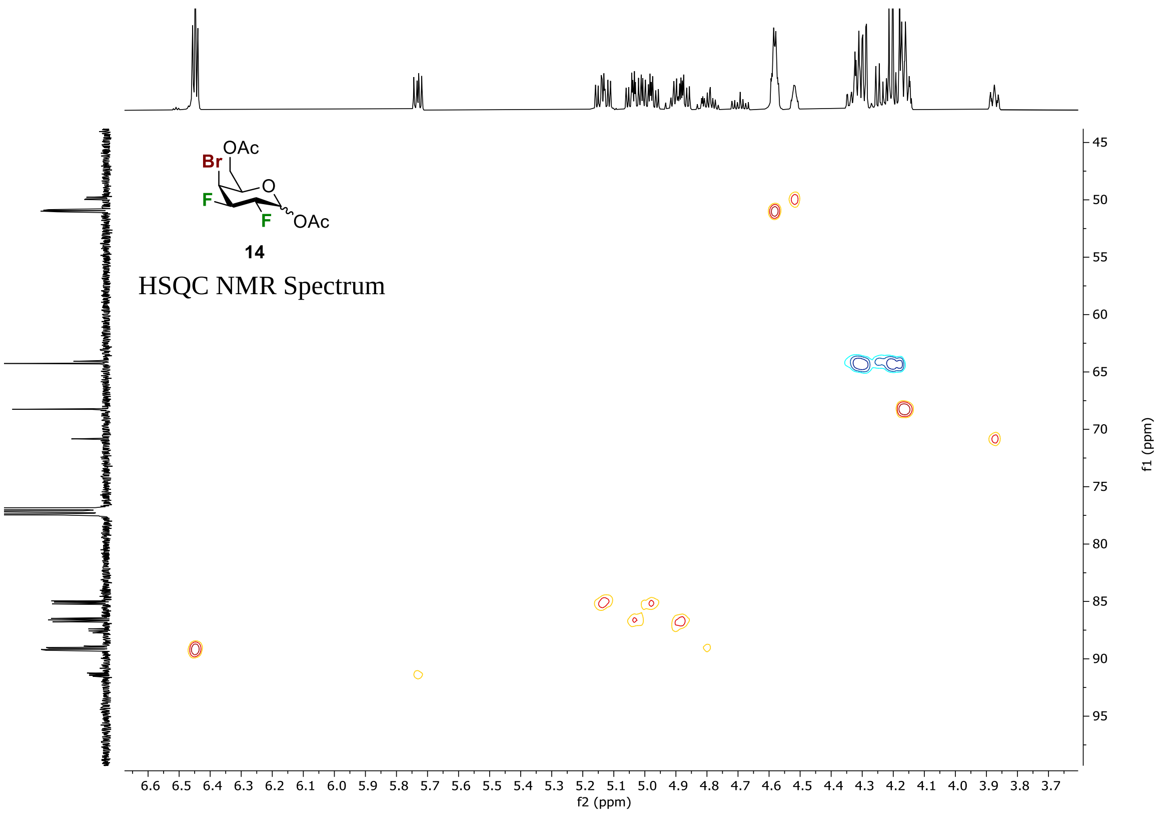
OAc
Br
F
O
F
OAc
14
HSQC NMR Spectrum
f2 (ppm)
f1 (ppm)

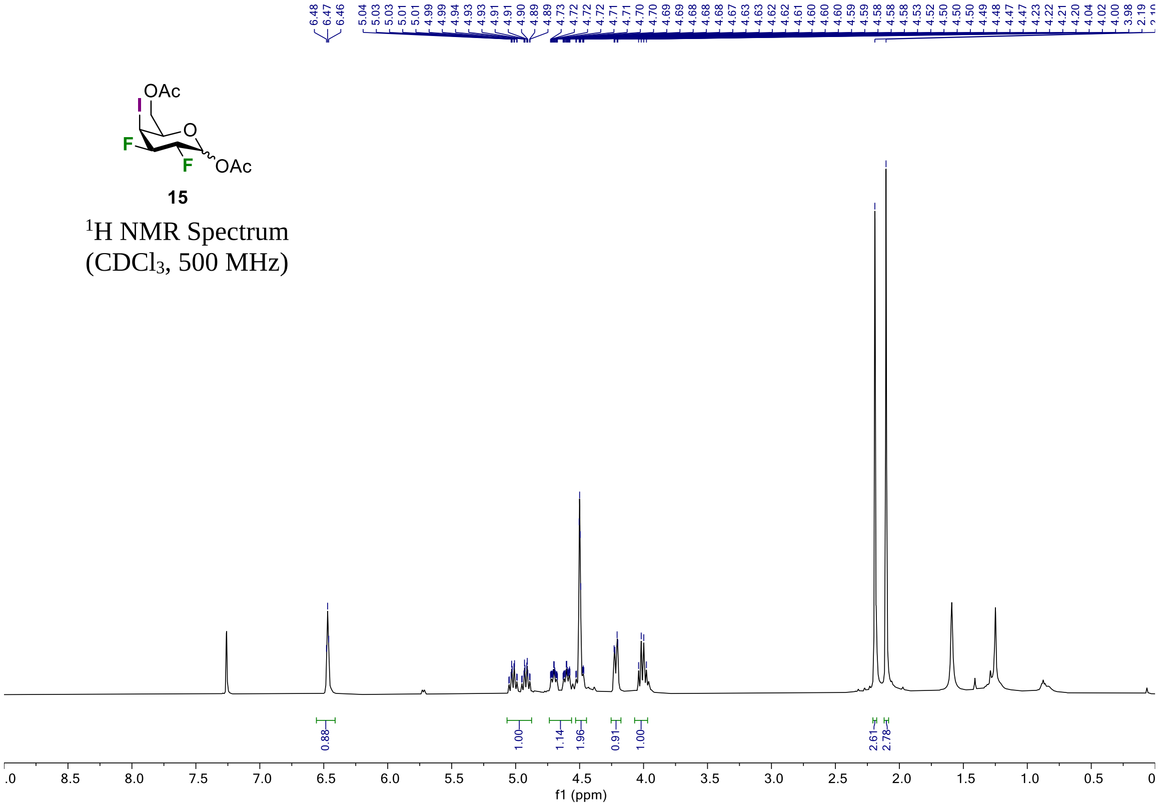
OAc
I
F
O
F
OAc
15
^{1}H NMR Spectrum
($CDCl_3$, 500 MHz)
f1 (ppm)

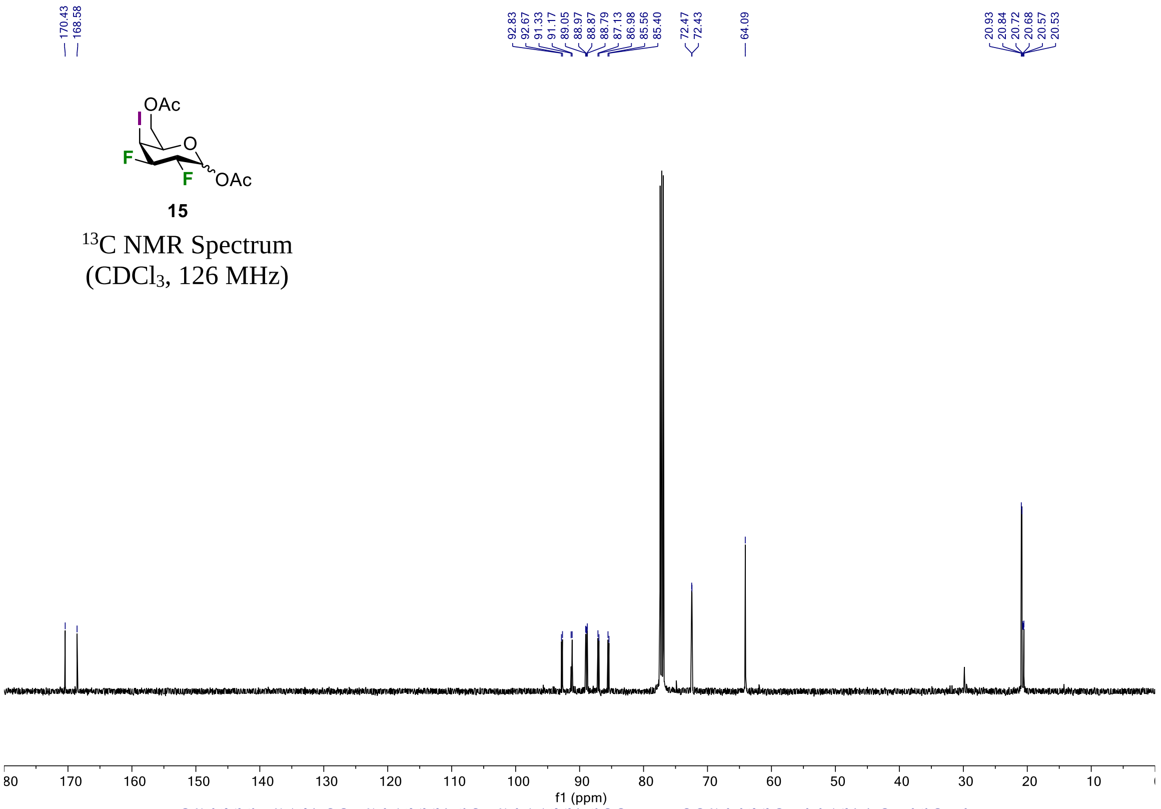
170.43
168.58
92.83
92.67
91.33
91.17
89.05
88.97
88.87
88.79
87.13
86.98
85.56
85.40
72.47
72.43
64.09
20.93
20.84
20.72
20.68
20.57
20.53
OAc
I
O
F
F
OAc
15
13C NMR Spectrum
(CDCl3, 126 MHz)
80
170
160
150
140
130
120
110
100
90
80
70
60
50
40
30
20
10
f1 (ppm)

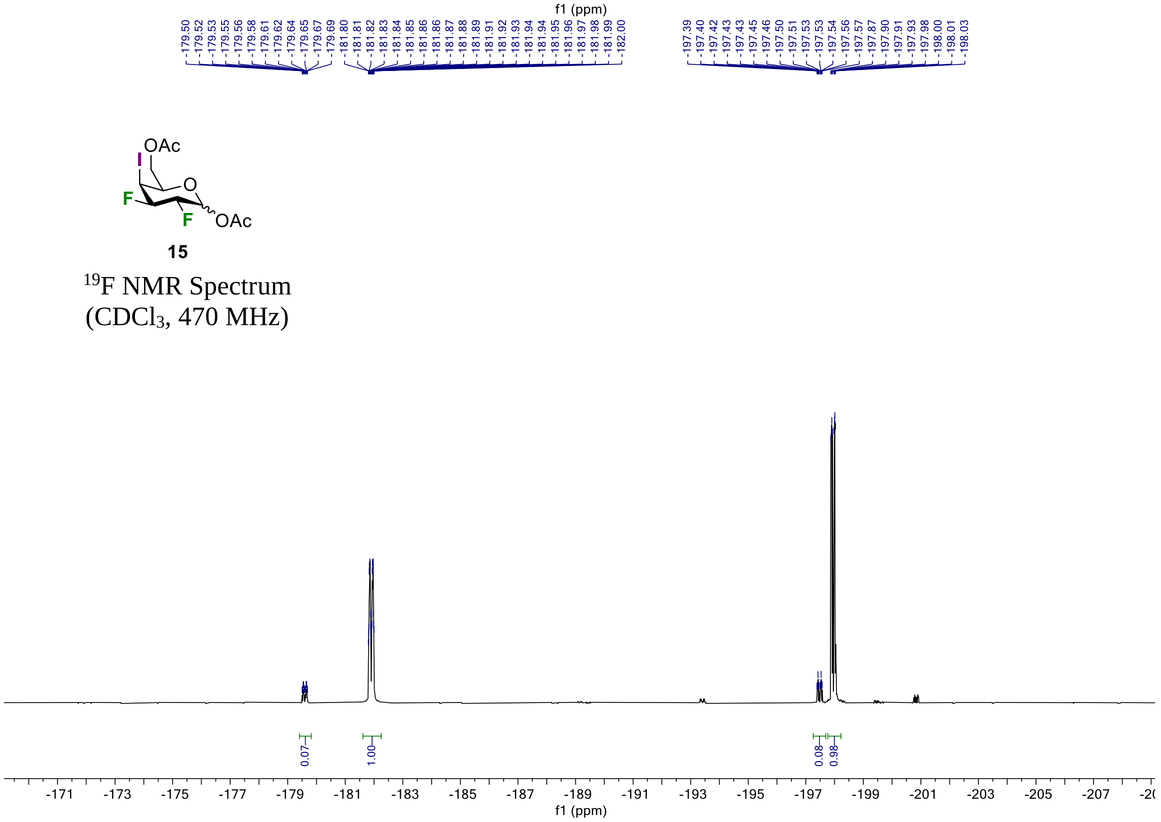
-179.50
-179.52
-179.53
-179.55
-179.56
-179.58
-179.61
-179.62
-179.64
-179.65
-179.67
-179.69
-181.80
-181.81
-181.82
-181.83
-181.84
-181.85
-181.86
-181.87
-181.88
-181.89
-181.91
-181.92
-181.93
-181.94
-181.94
-181.95
-181.96
-181.97
-181.98
-181.99
-182.00
-197.39
-197.40
-197.42
-197.43
-197.43
-197.45
-197.46
-197.50
-197.51
-197.53
-197.54
-197.56
-197.57
-197.87
-197.90
-197.91
-197.93
-197.98
-198.00
-198.01
-198.03
OAc
I
O
F
F
OAc
15
19F NMR Spectrum
(CDCl3, 470 MHz)
0.07
1.00
0.08
0.98
-171
-173
-175
-177
-179
-181
-183
-185
-187
-189
-191
-193
-195
-197
-199
-201
-203
-205
-207
f1 (ppm)

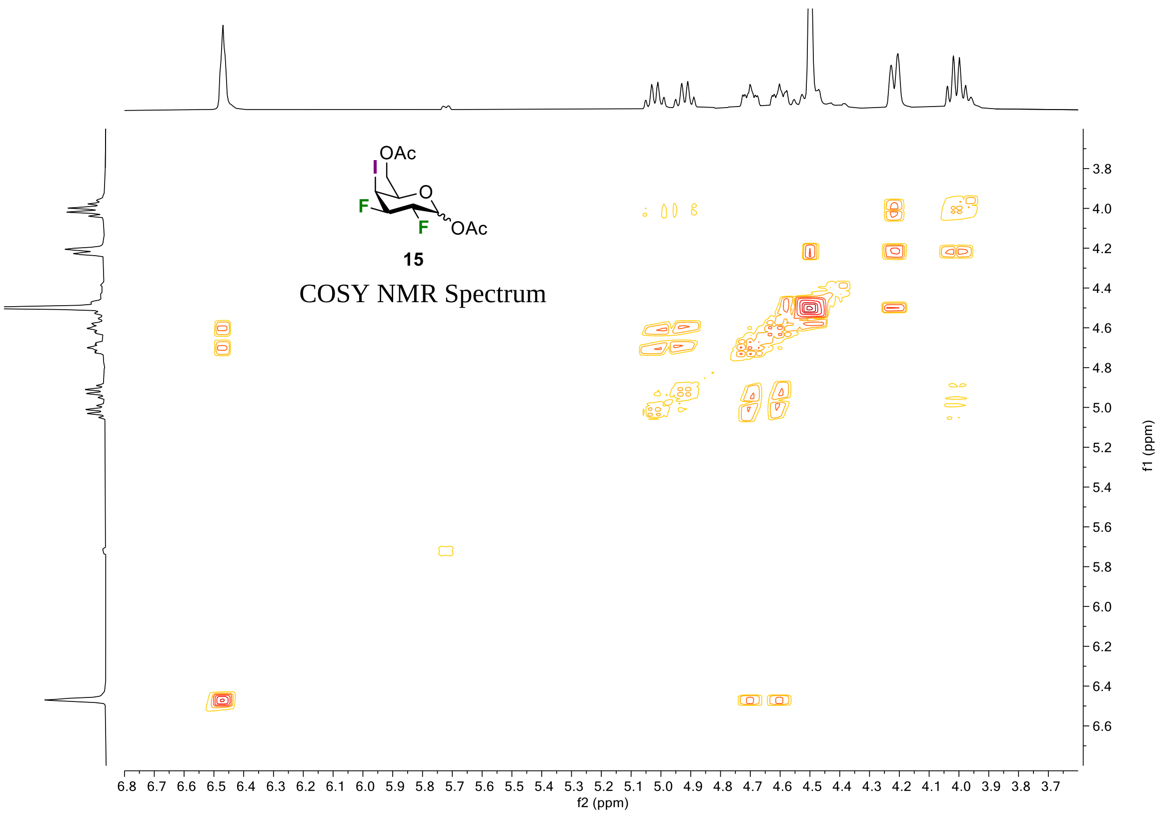

OAc
I
O
F
F
OAc
15
COSY NMR Spectrum
f2 (ppm)
f1 (ppm)

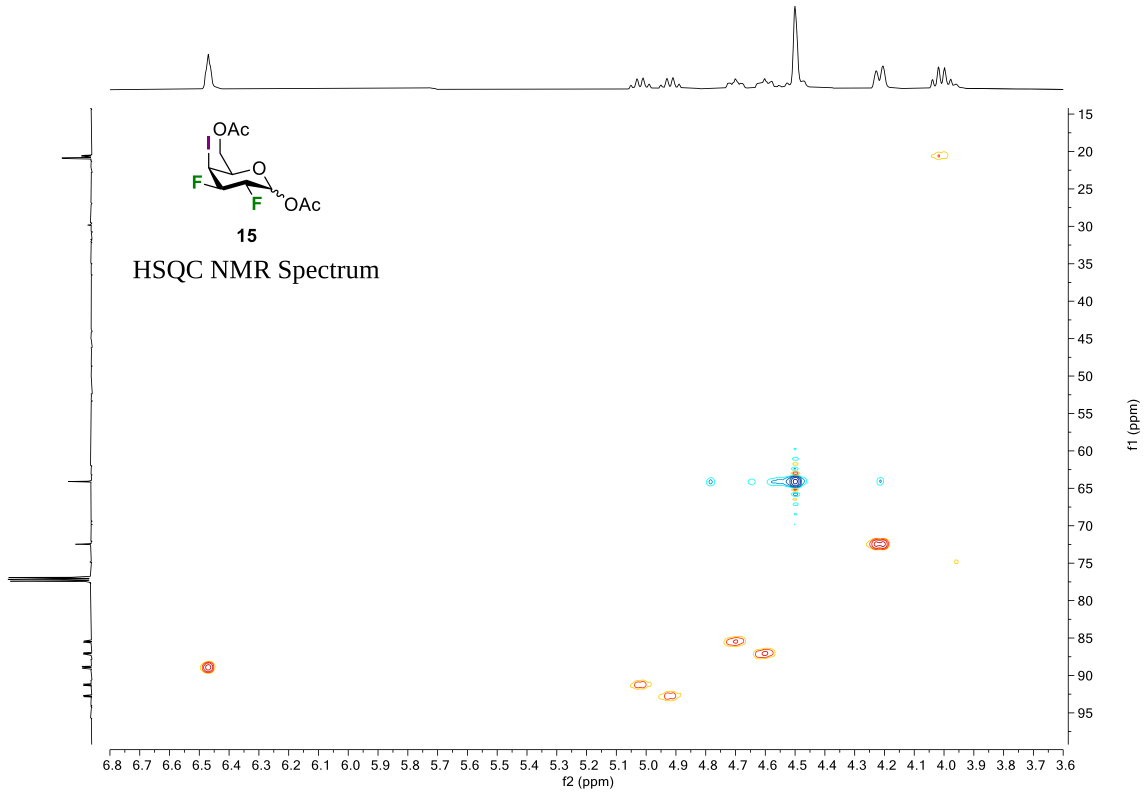

OAc
I
O
F
F
OAc
15
HSQC NMR Spectrum
f2 (ppm)
f1 (ppm)

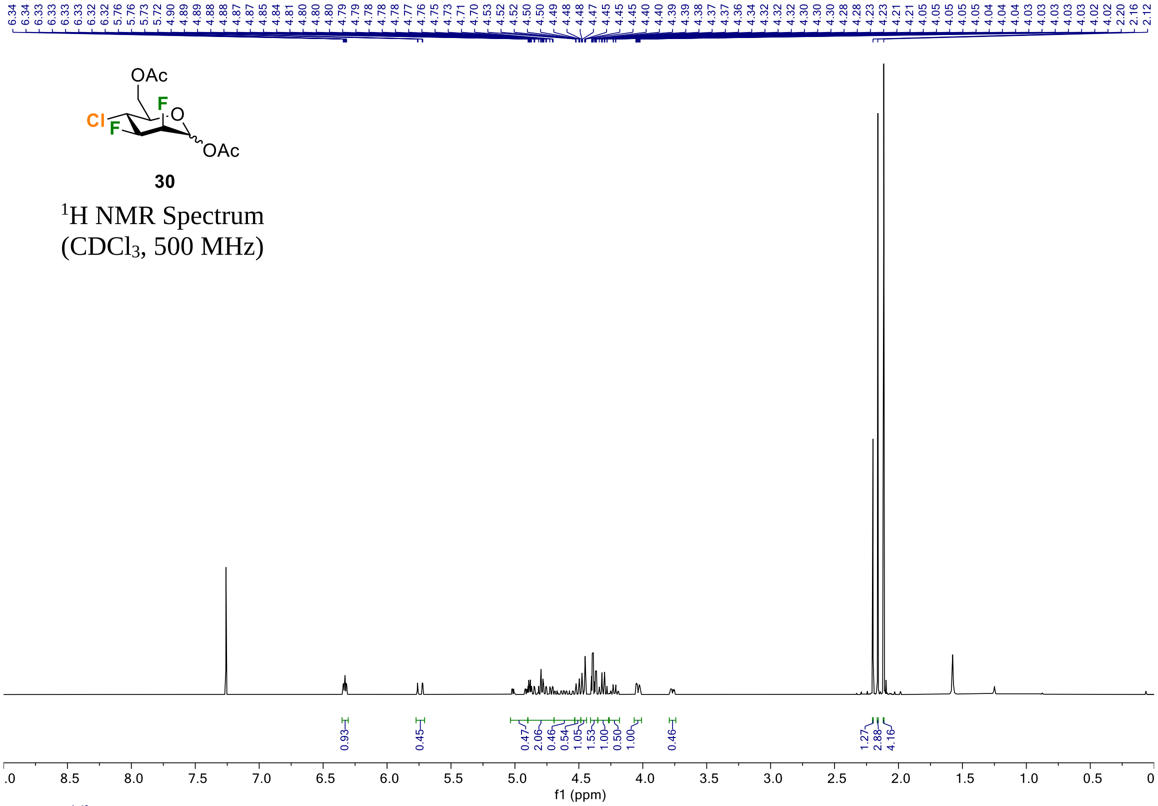

30

^{1}H NMR Spectrum
($CDCl_3$, 500 MHz)

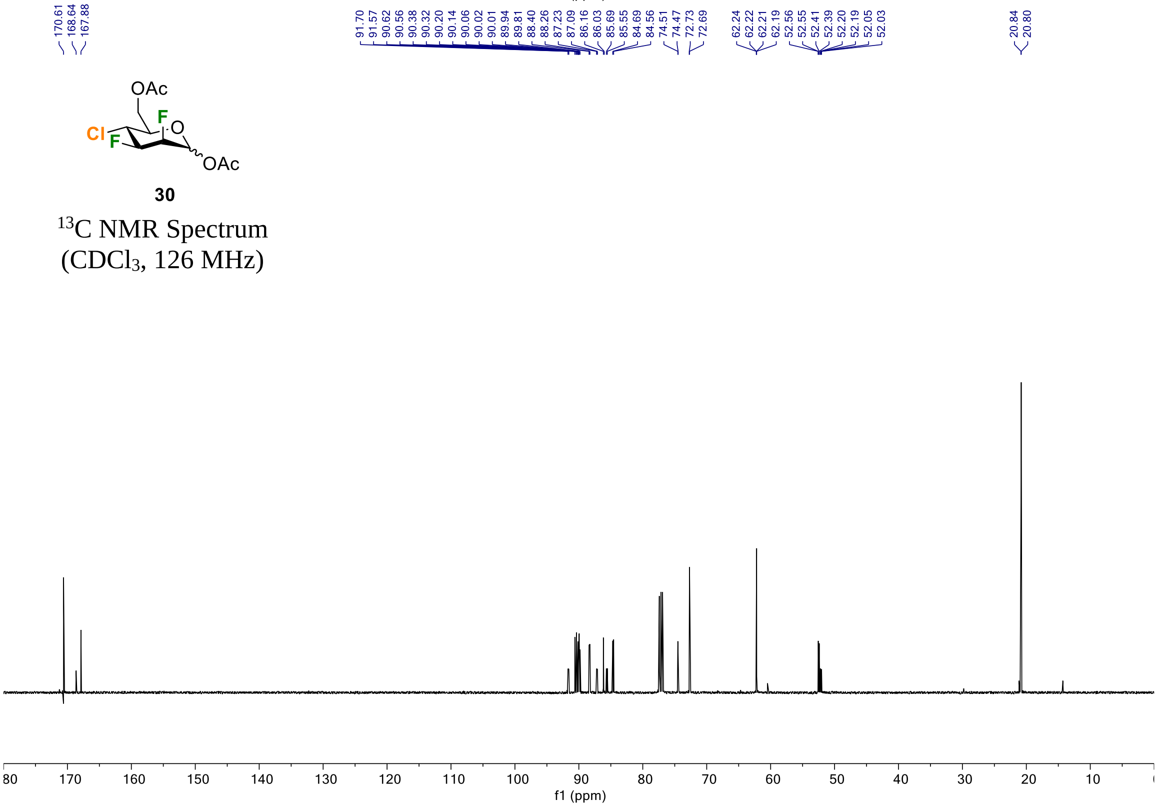

30

^{13}C NMR Spectrum
($CDCl_3$, 126 MHz)

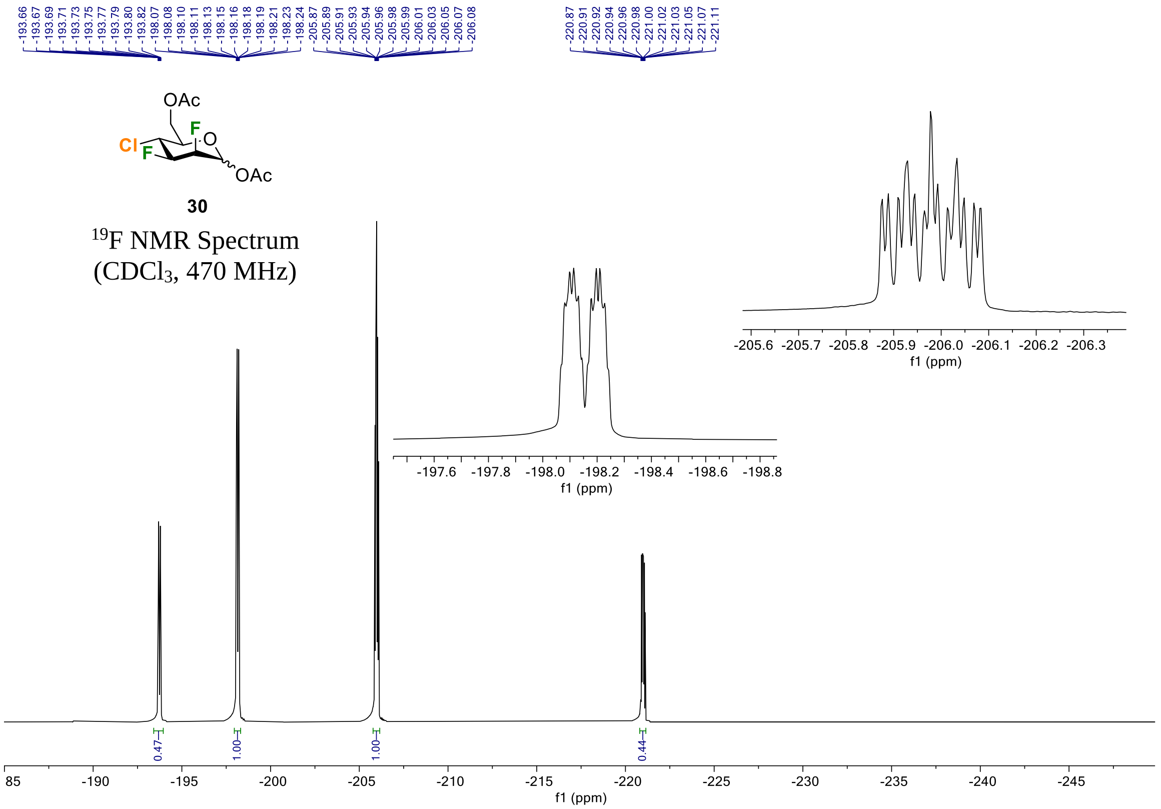

OAc
Cl
F
F
O
OAc
30
^{19}F NMR Spectrum
($CDCl_3$, 470 MHz)
f1 (ppm)

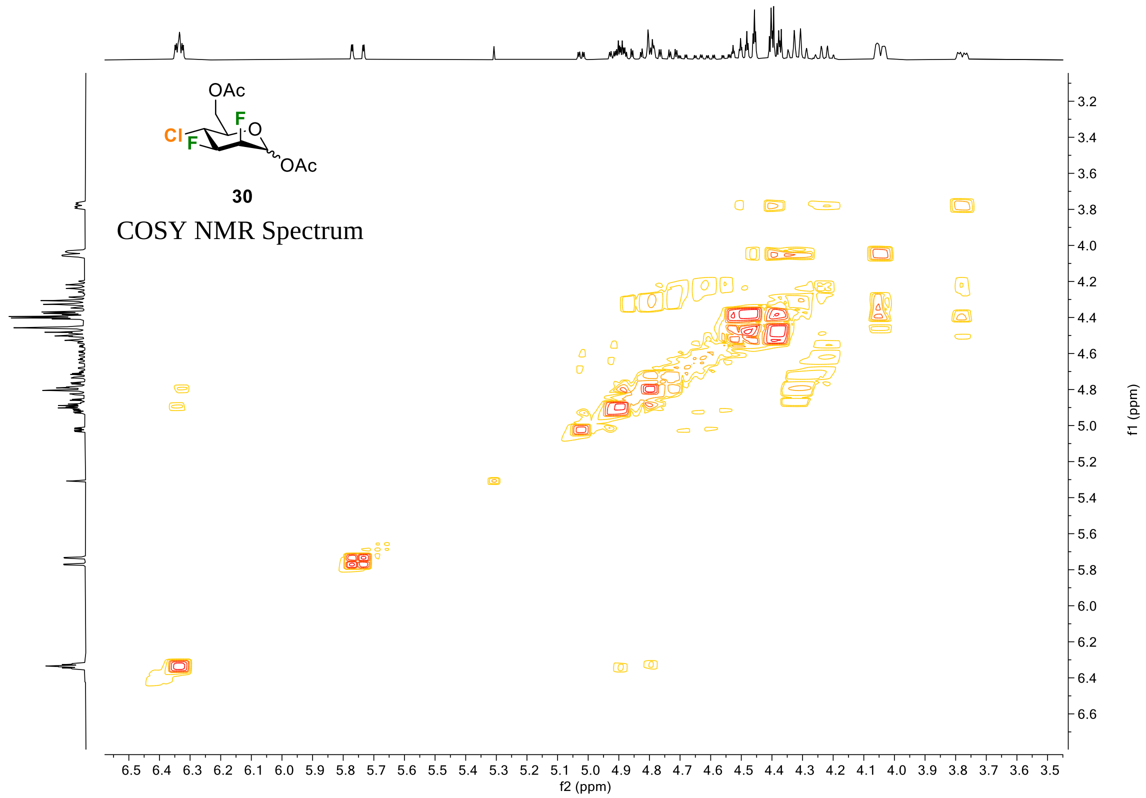

OAc
Cl
F
F
O
OAc
30
COSY NMR Spectrum
f2 (ppm)
f1 (ppm)

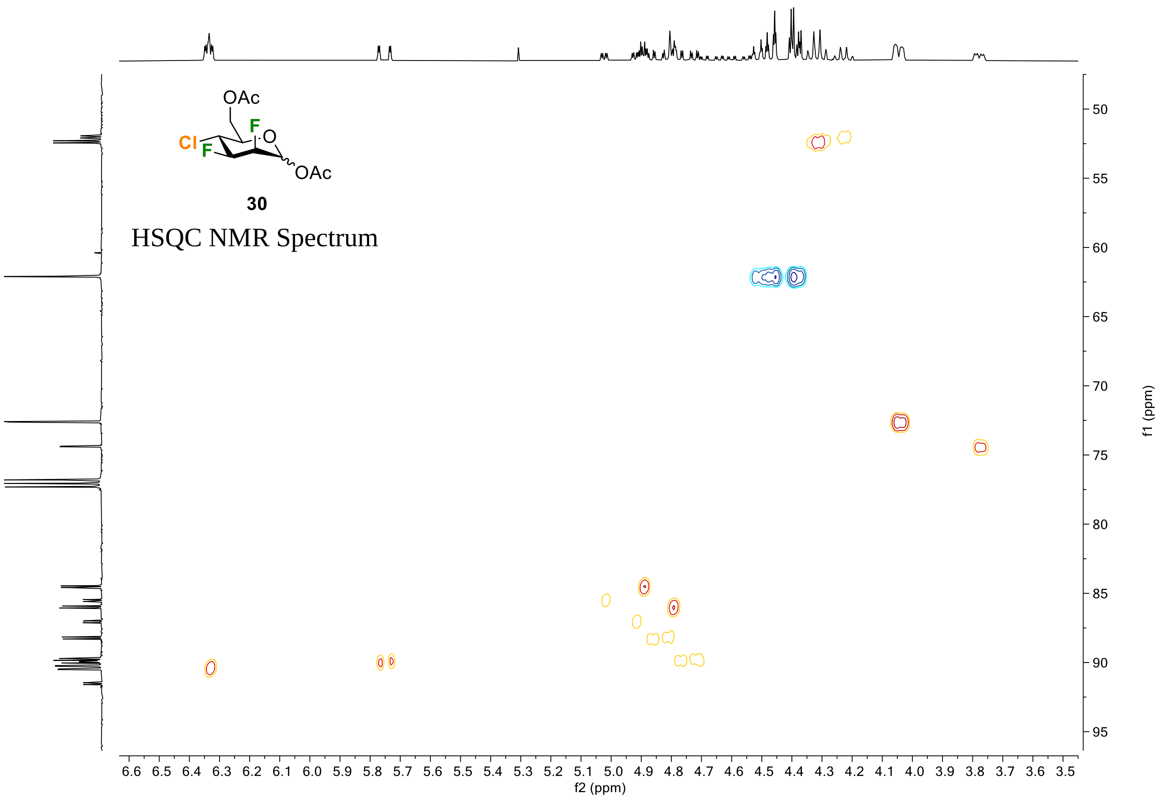
OAc
Cl
F
F
O
OAc
30
HSQC NMR Spectrum
f2 (ppm)
f1 (ppm)

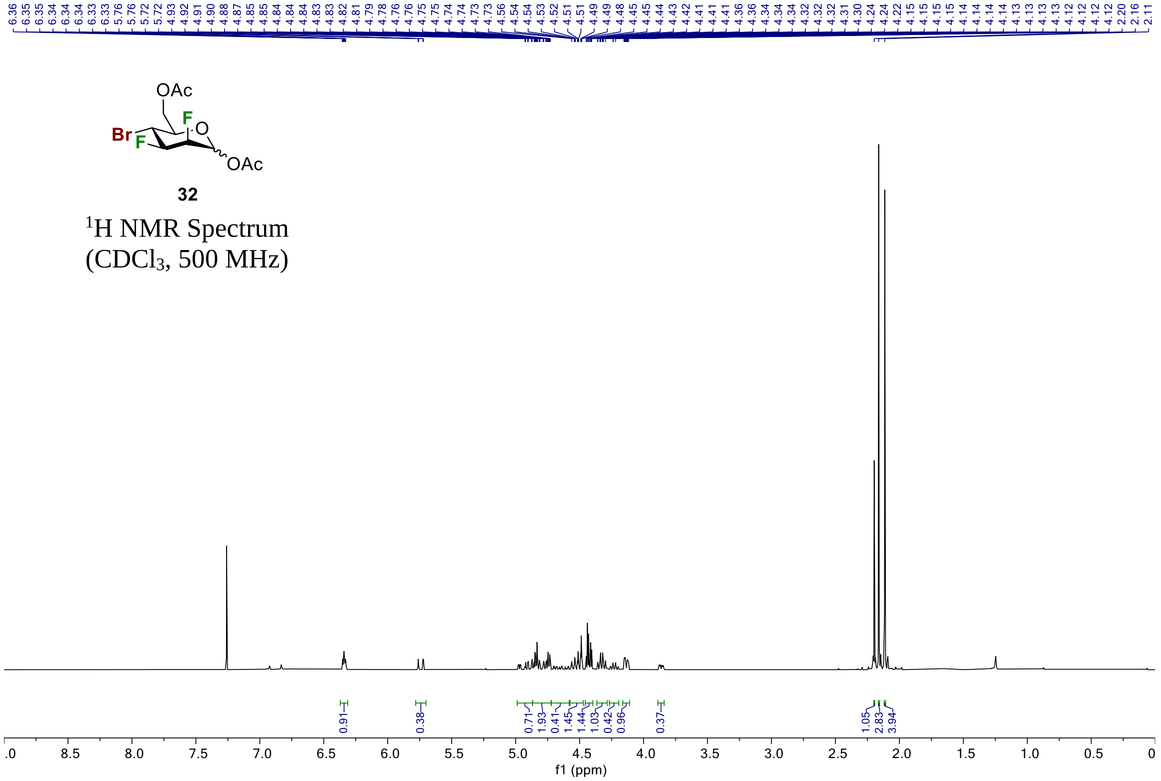
OAc
Br
F
F
O
OAc
32
1H NMR Spectrum
(CDCl3, 500 MHz)
f1 (ppm)

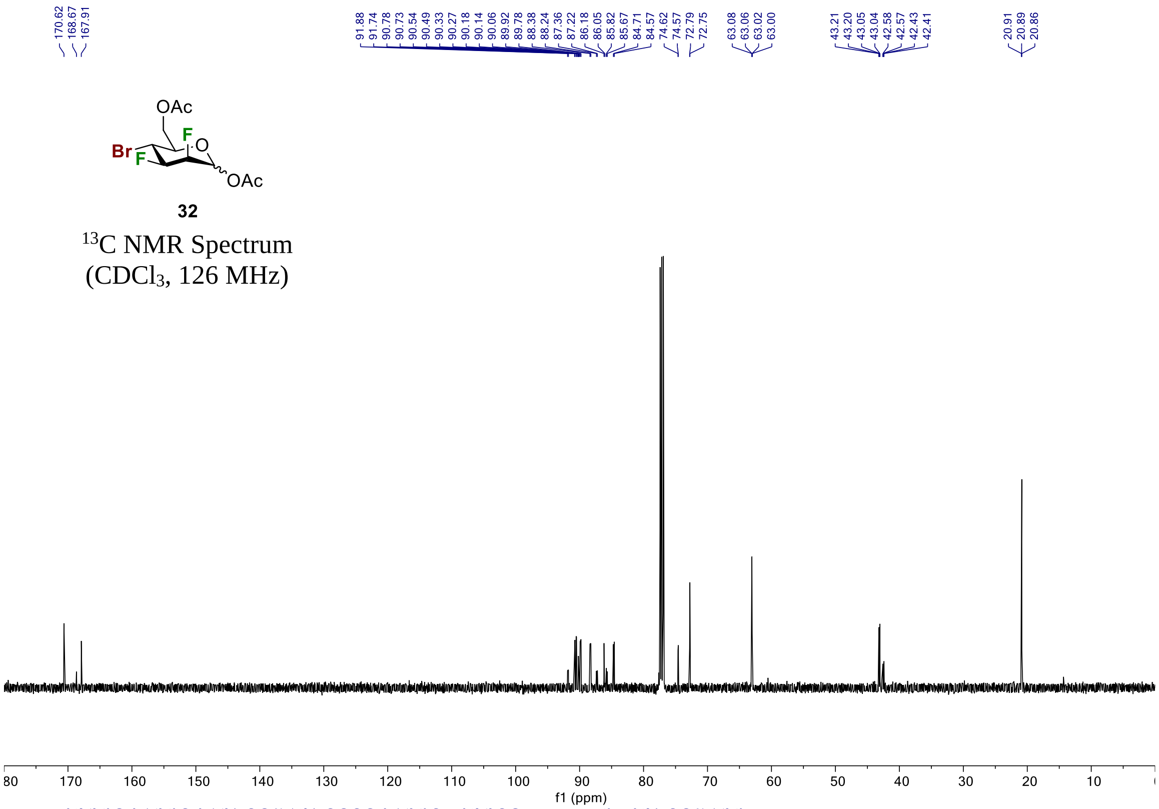
170.62
168.67
167.91
91.88
91.74
90.78
90.73
90.54
90.49
90.33
90.27
90.18
90.14
90.06
89.92
89.78
88.38
88.24
87.36
87.22
86.18
86.05
85.82
85.67
84.71
84.57
74.62
74.57
72.79
72.75
63.08
63.06
63.02
63.00
43.21
43.20
43.05
43.04
42.58
42.57
42.43
42.41
20.91
20.89
20.86
OAc
Br
F
F
O
OAc
32
13C NMR Spectrum
(CDCl3, 126 MHz)
f1 (ppm)

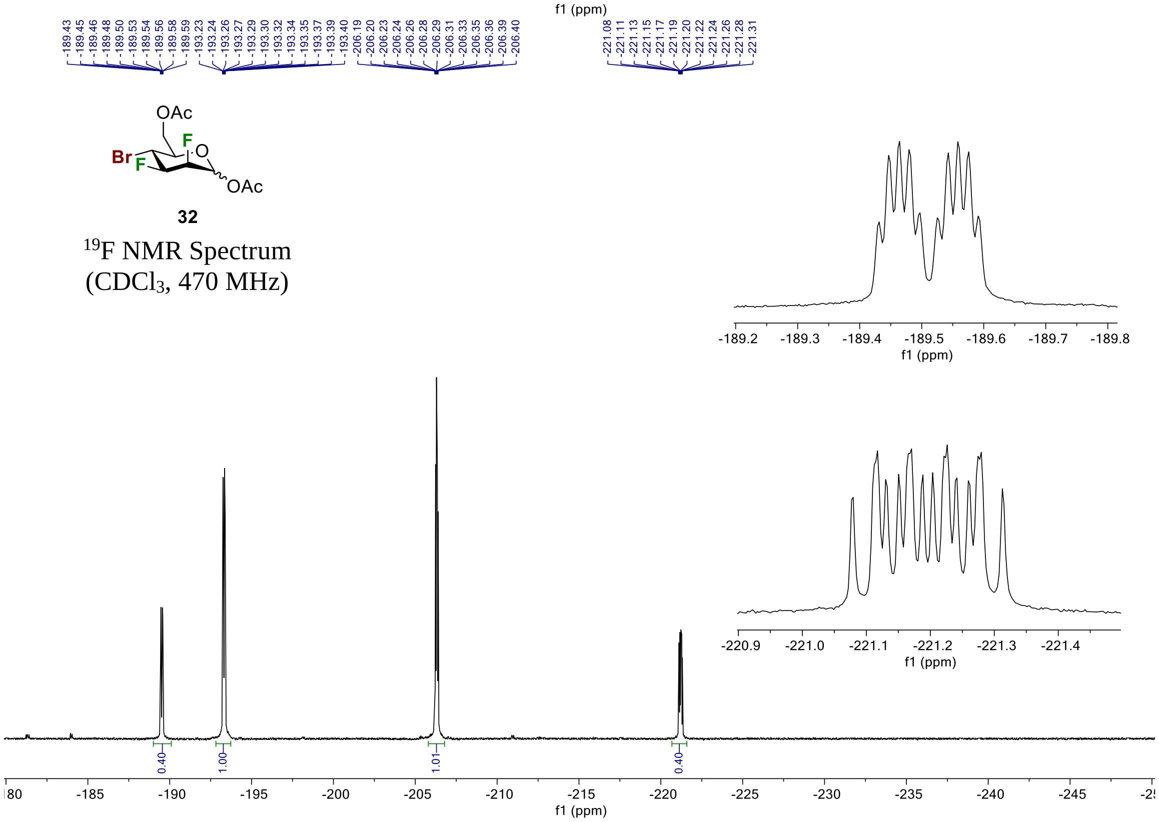
-189.43
-189.45
-189.46
-189.48
-189.50
-189.53
-189.54
-189.56
-189.58
-189.59
-193.23
-193.24
-193.26
-193.27
-193.29
-193.30
-193.32
-193.34
-193.35
-193.37
-193.39
-193.40
-206.19
-206.20
-206.23
-206.24
-206.26
-206.28
-206.29
-206.31
-206.33
-206.35
-206.36
-206.39
-206.40
-221.08
-221.11
-221.13
-221.15
-221.17
-221.19
-221.20
-221.22
-221.24
-221.26
-221.28
-221.31
OAc
Br
F
F
O
OAc
32
19F NMR Spectrum
(CDCl3, 470 MHz)
0.40
1.00
1.01
0.40
f1 (ppm)

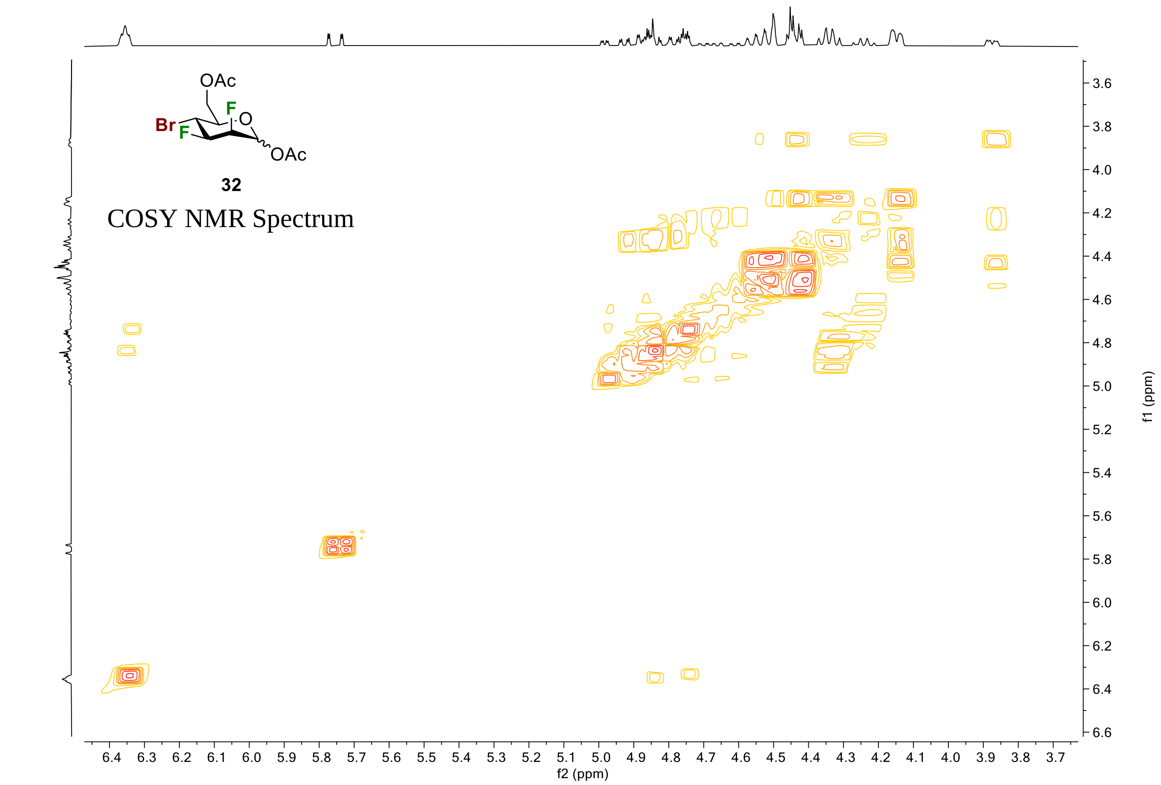
OAc
Br
F
F
O
OAc
32
COSY NMR Spectrum
f2 (ppm)
f1 (ppm)

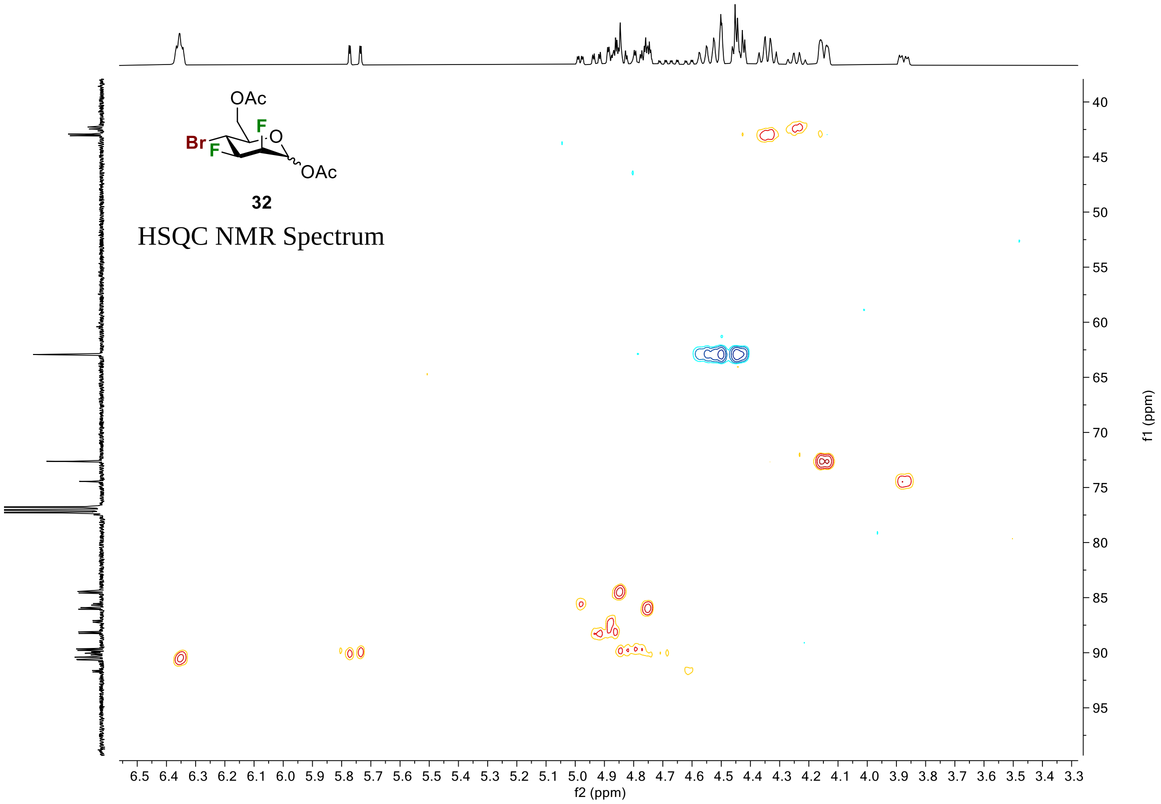
OAc
Br
F
F
O
OAc
32
HSQC NMR Spectrum
f2 (ppm)
f1 (ppm)

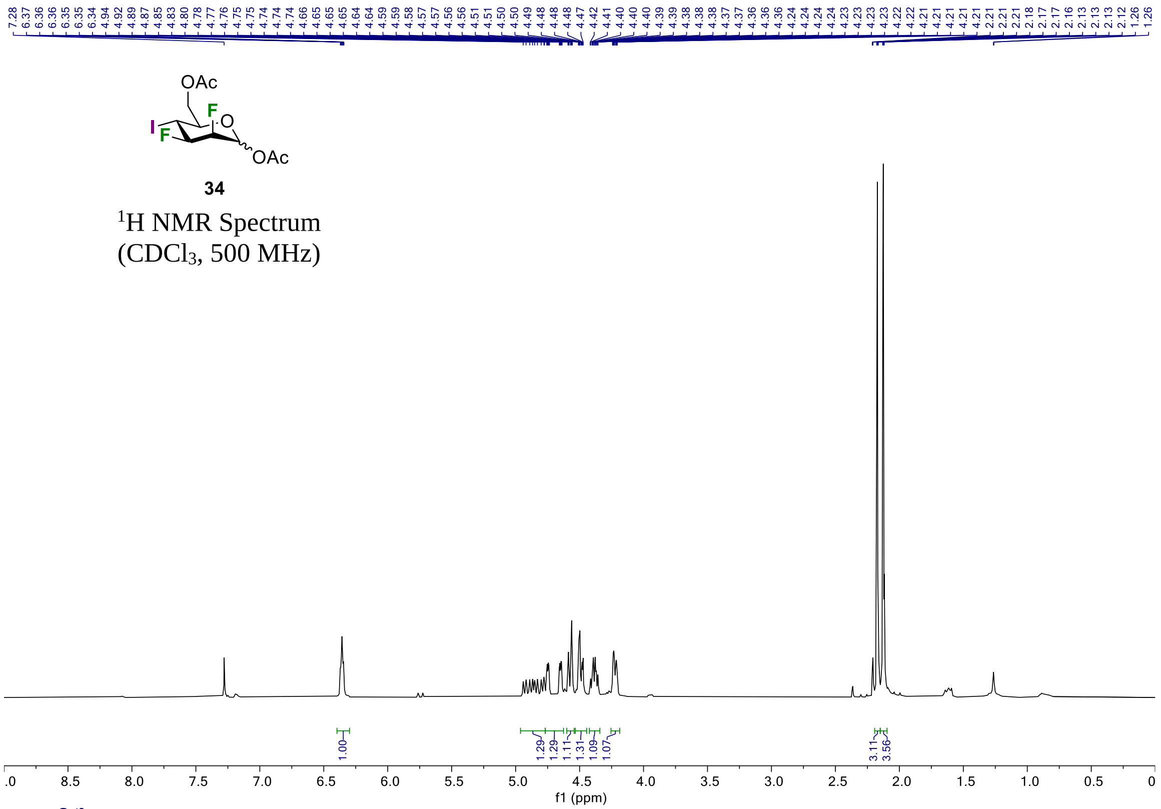
OAc
F
I
F
O
OAc
34
^{1}H NMR Spectrum
($CDCl_3$, 500 MHz)
f1 (ppm)

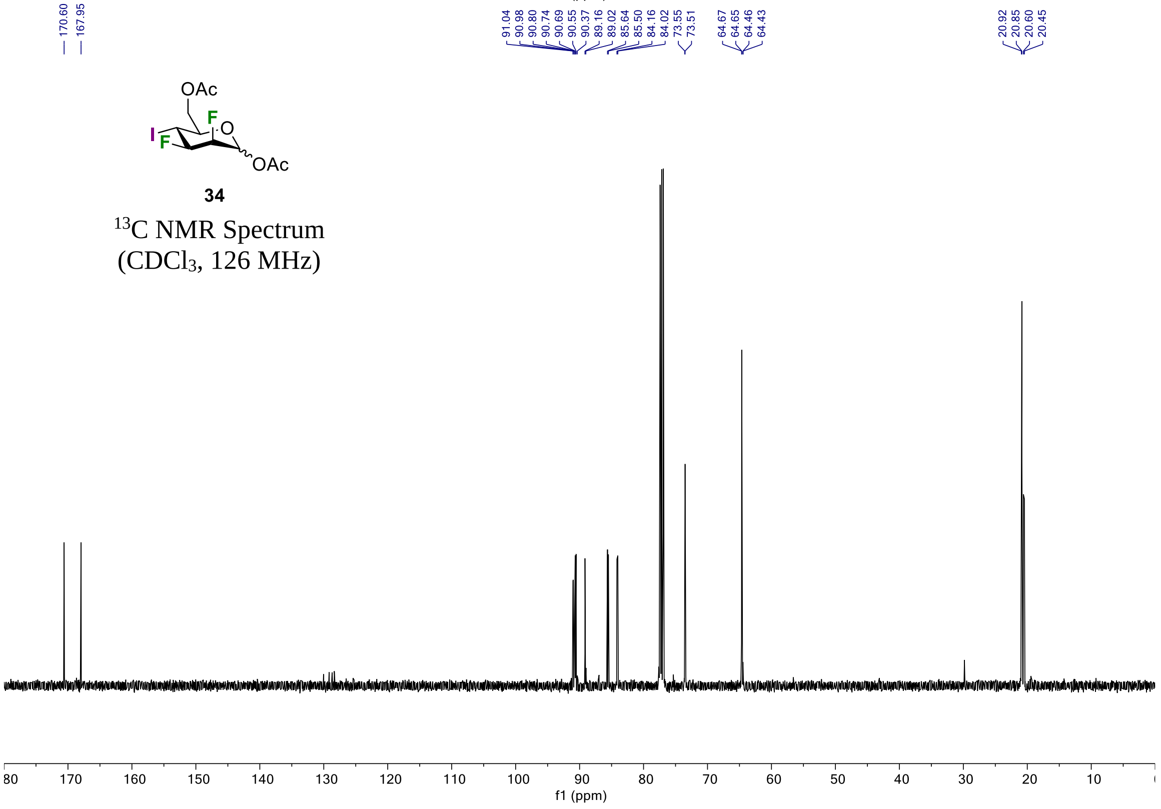
OAc
F
I
F
O
OAc
34
^{13}C NMR Spectrum
($CDCl_3$, 126 MHz)
f1 (ppm)

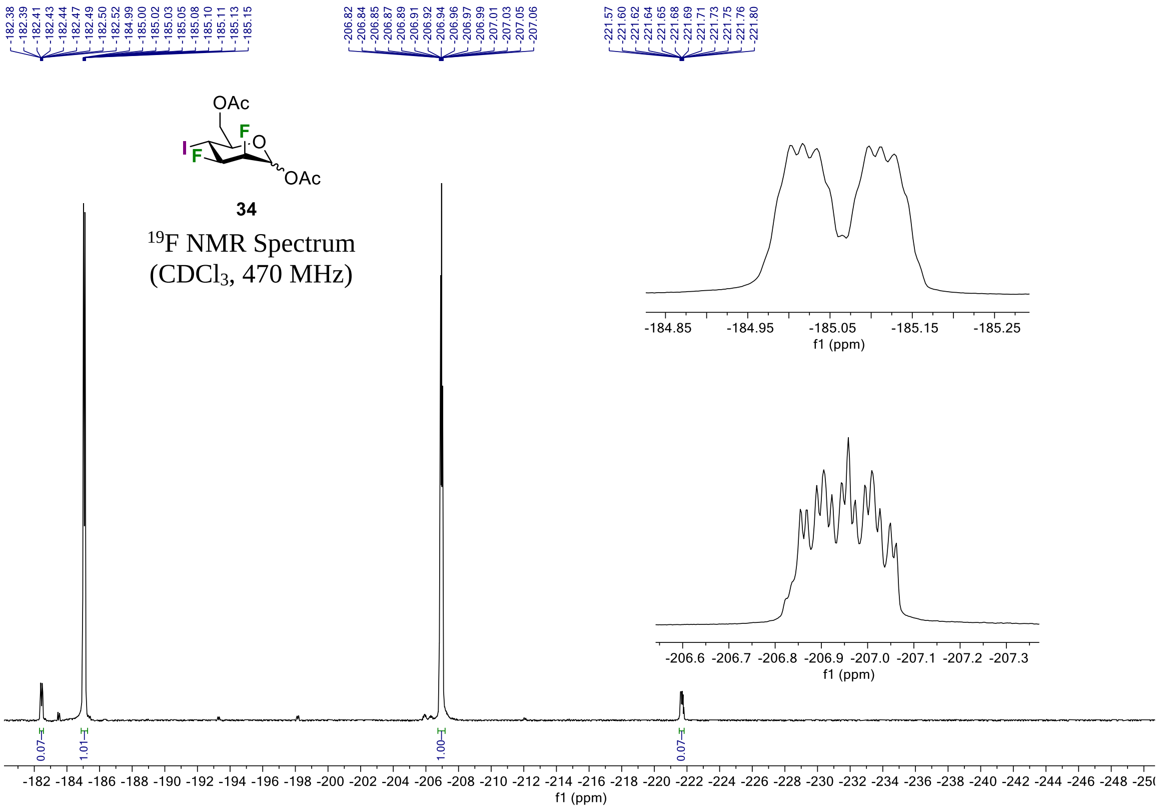
OAc
F
O
I
F
OAc
34
19F NMR Spectrum
(CDCl3, 470 MHz)
f1 (ppm)

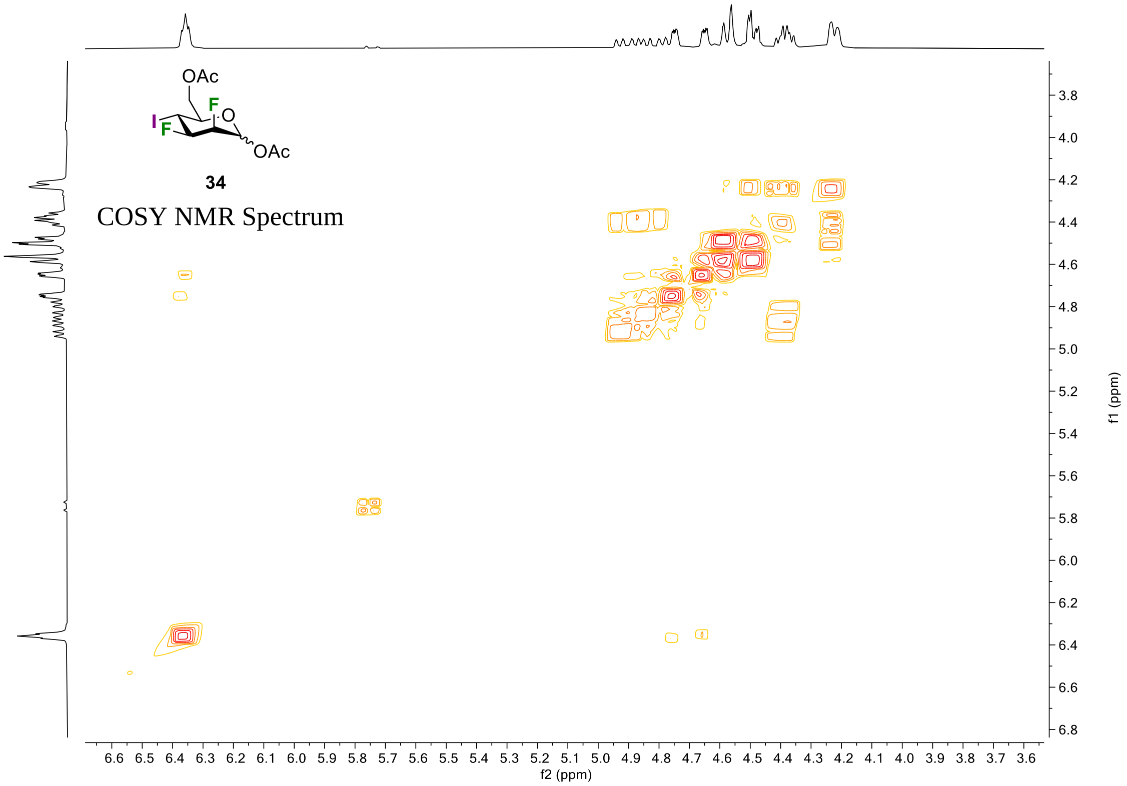
OAc
F
O
I
F
OAc
34
COSY NMR Spectrum
f2 (ppm)
f1 (ppm)

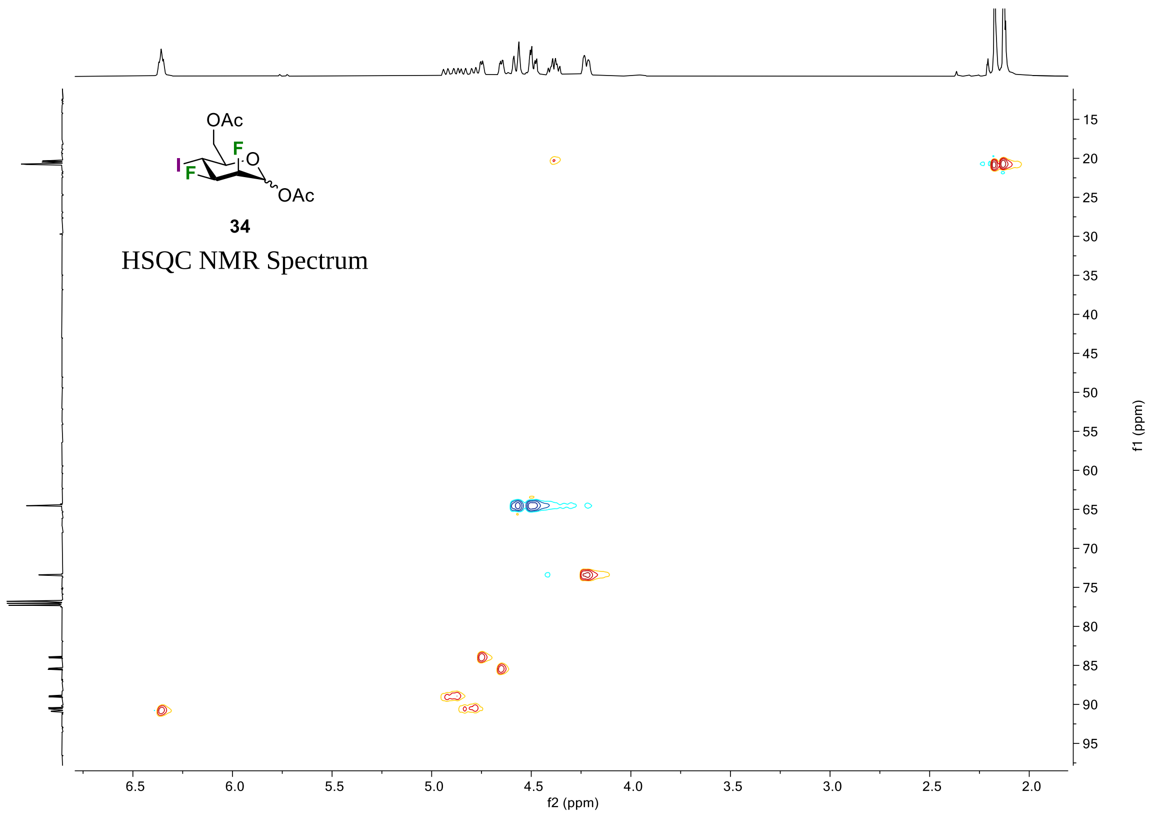

OAc
F
O
I
F
OAc
34
HSQC NMR Spectrum
f2 (ppm)
f1 (ppm)

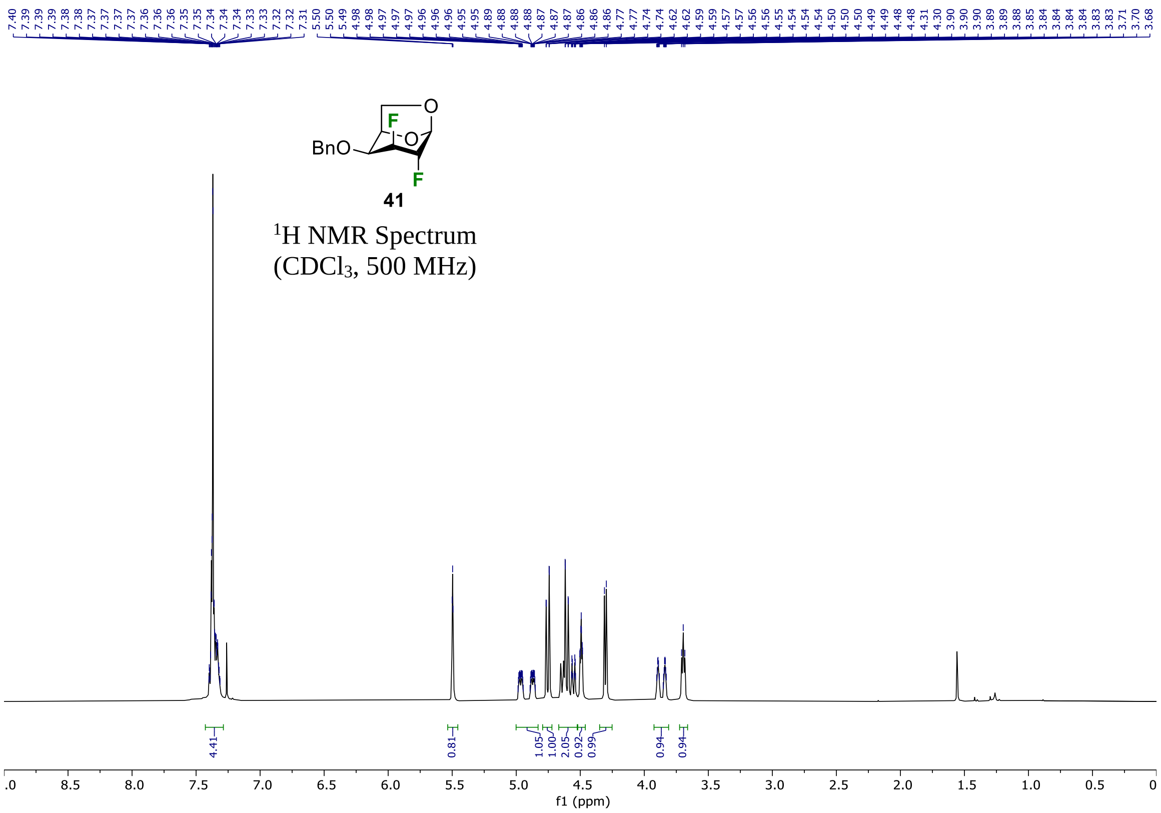

BnO
F
O
O
F
41
^{1}H NMR Spectrum
($CDCl_3$, 500 MHz)
f1 (ppm)

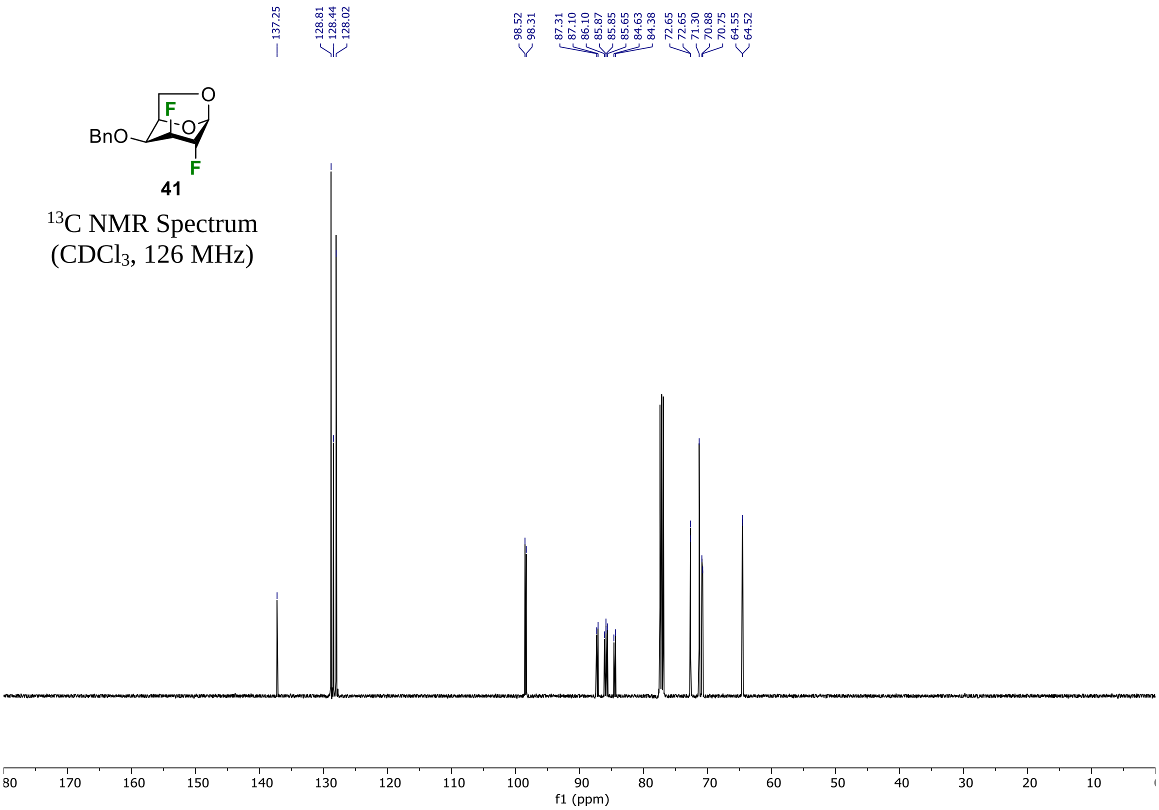
137.25
128.81
128.44
128.02
98.52
98.31
87.31
87.10
86.10
85.87
85.85
85.65
84.63
84.38
72.65
72.65
71.30
70.88
70.75
64.55
64.52
BnO
F
O
O
F
41
13C NMR Spectrum
(CDCl3, 126 MHz)
f1 (ppm)

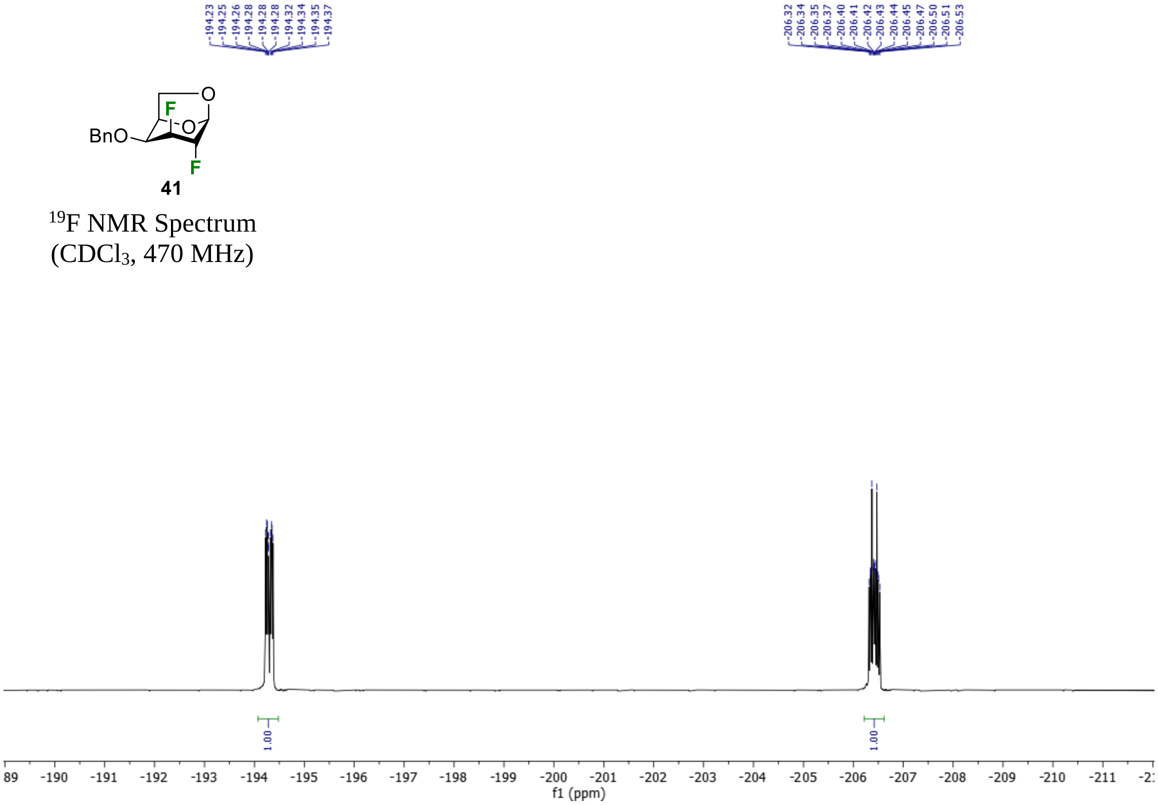
-194.23
-194.25
-194.26
-194.28
-194.28
-194.28
-194.32
-194.34
-194.35
-194.37
-206.32
-206.34
-206.35
-206.37
-206.40
-206.41
-206.42
-206.43
-206.44
-206.45
-206.47
-206.50
-206.51
-206.53
BnO
F
O
O
F
41
19F NMR Spectrum
(CDCl3, 470 MHz)
1.00
1.00
f1 (ppm)

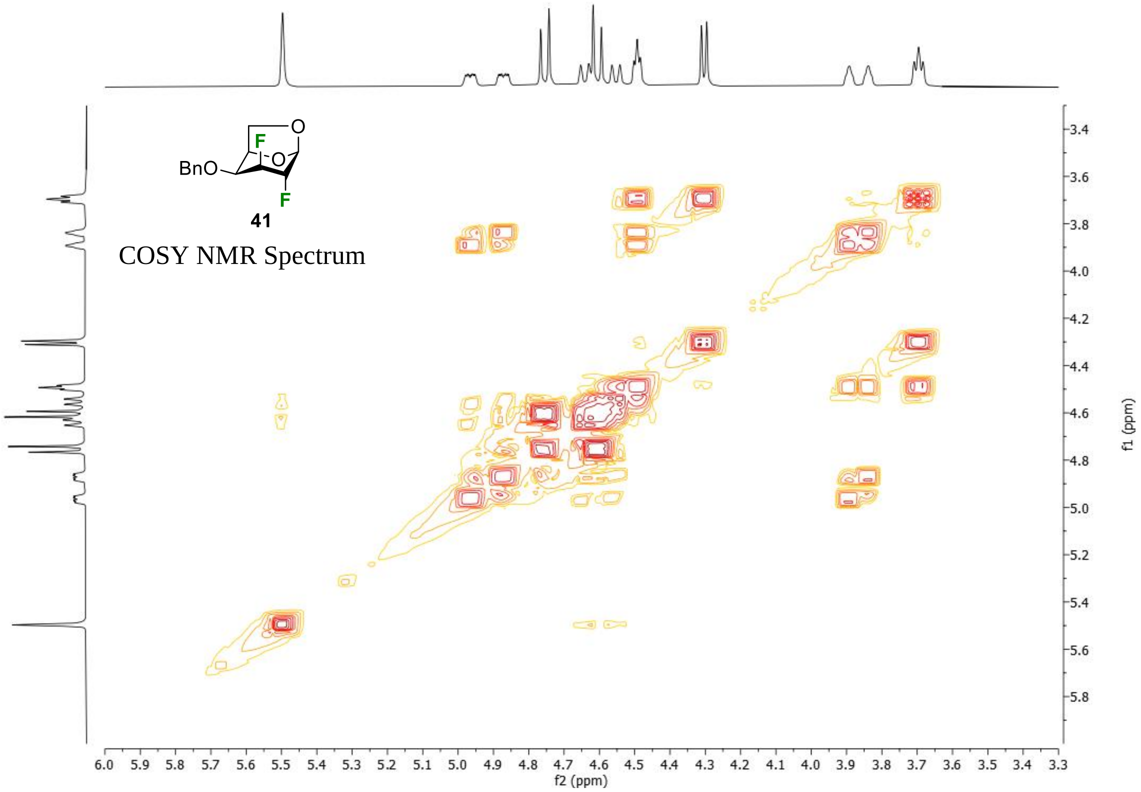
BnO
F
O
F
41
COSY NMR Spectrum
f2 (ppm)
f1 (ppm)

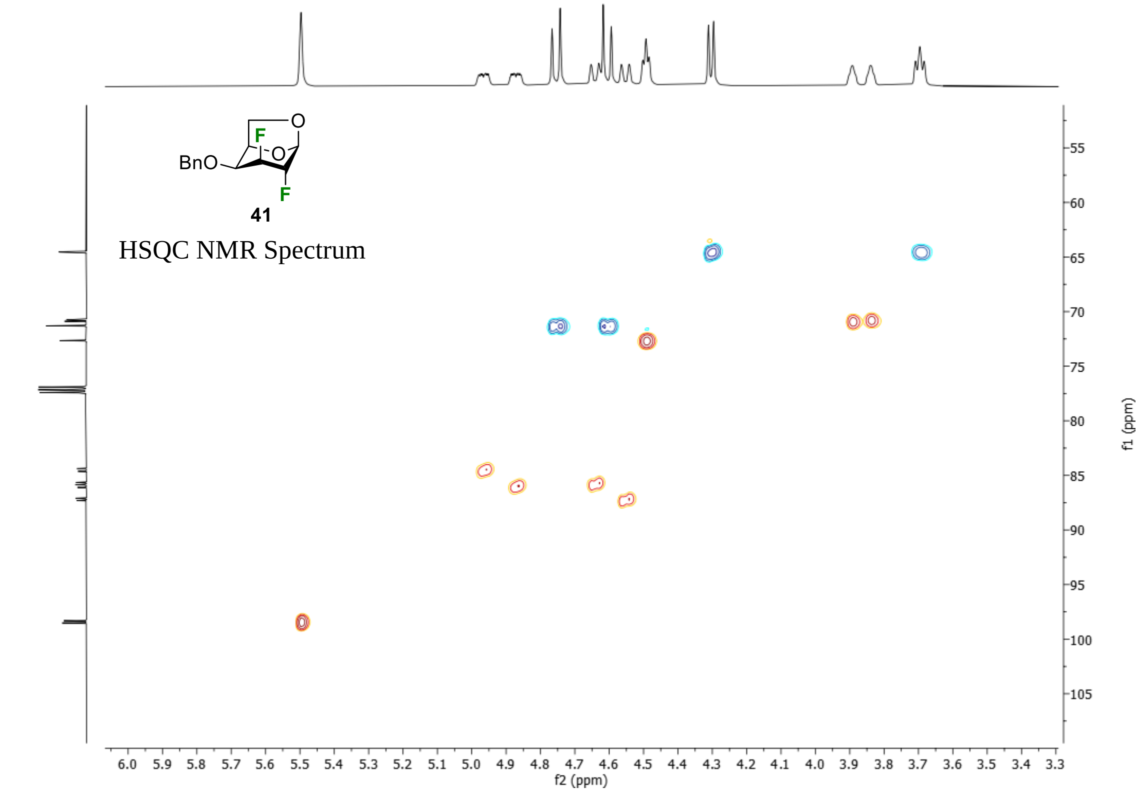
BnO
F
O
F
41
HSQC NMR Spectrum
f2 (ppm)
f1 (ppm)

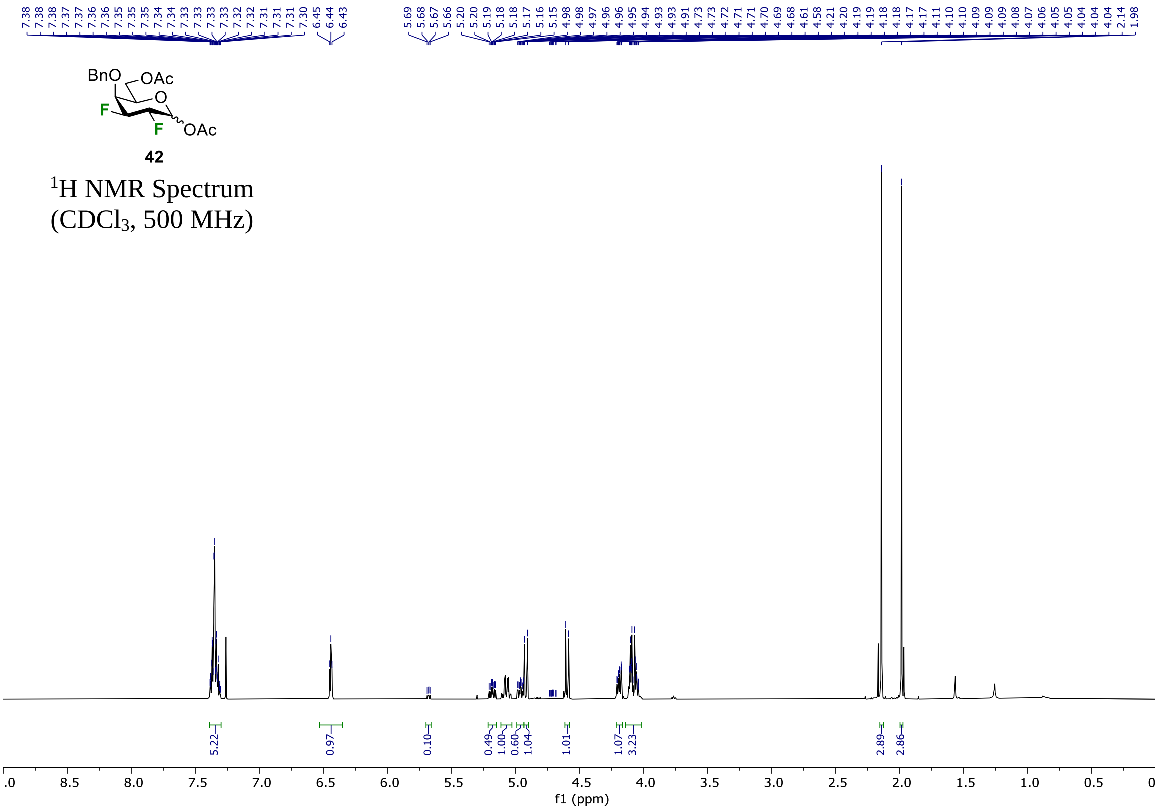
7.38 7.38 7.38 7.37 7.37 7.36 7.36 7.35 7.35 7.35 7.34 7.34 7.33 7.33 7.33 7.33 7.32 7.32 7.31 7.31 7.30 6.45 6.44 6.43 5.69 5.68 5.67 5.66 5.20 5.20 5.19 5.18 5.18 5.17 5.16 5.15 4.98 4.98 4.97 4.96 4.96 4.95 4.94 4.93 4.93 4.93 4.91 4.73 4.73 4.72 4.71 4.71 4.70 4.69 4.68 4.61 4.58 4.21 4.20 4.19 4.19 4.18 4.18 4.17 4.17 4.11 4.10 4.10 4.09 4.09 4.09 4.08 4.07 4.06 4.05 4.04 4.04 4.04 2.14 1.98
BnO
OAc
O
F
F
OAc
42
¹H NMR Spectrum
(CDCl₃, 500 MHz)
5.22 0.97 0.10 0.49 1.00 0.60 1.04 1.01 1.07 3.23 2.89 2.86
.0 8.5 8.0 7.5 7.0 6.5 6.0 5.5 5.0 4.5 4.0 3.5 3.0 2.5 2.0 1.5 1.0 0.5 0
f1 (ppm)

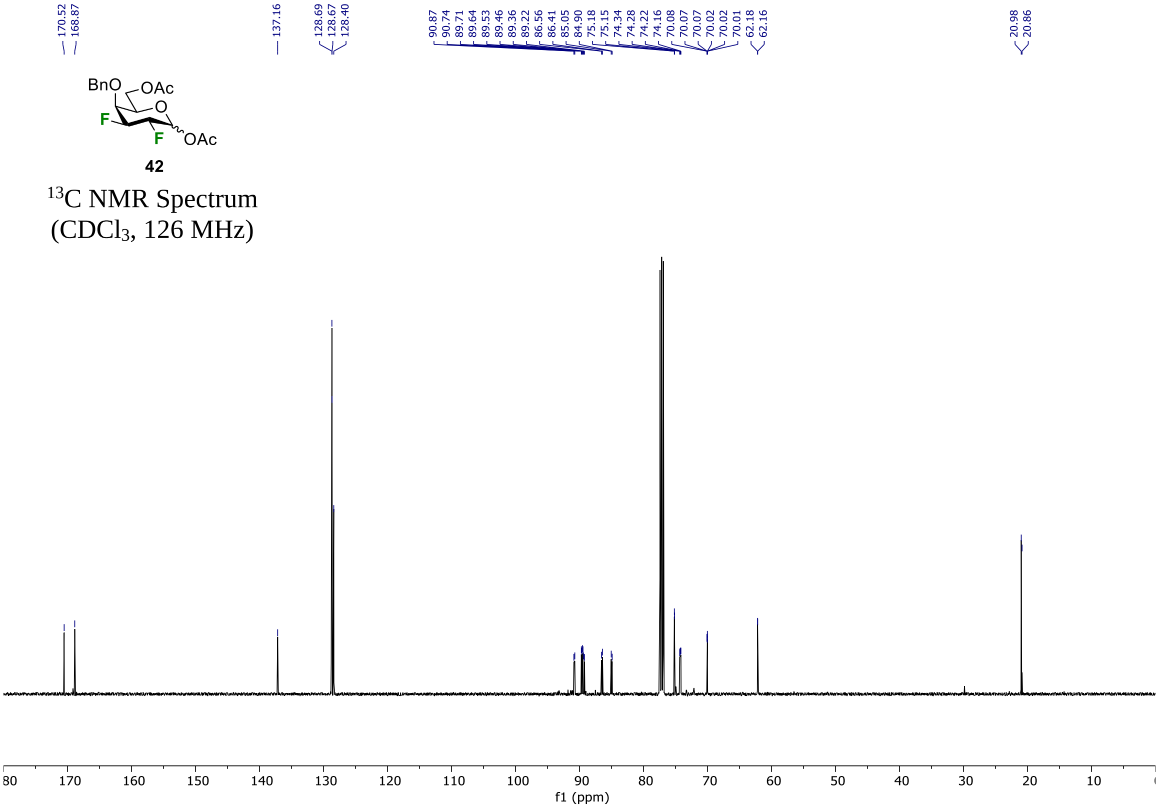
170.52 168.87 137.16 128.69 128.67 128.40 90.87 90.74 89.71 89.64 89.53 89.46 89.36 89.22 86.56 86.41 85.05 84.90 75.18 75.15 74.34 74.28 74.22 74.16 70.08 70.07 70.07 70.02 70.02 70.01 62.18 62.16 20.98 20.86
BnO
OAc
O
F
F
OAc
42
¹³C NMR Spectrum
(CDCl₃, 126 MHz)
80 170 160 150 140 130 120 110 100 90 80 70 60 50 40 30 20 10
f1 (ppm)

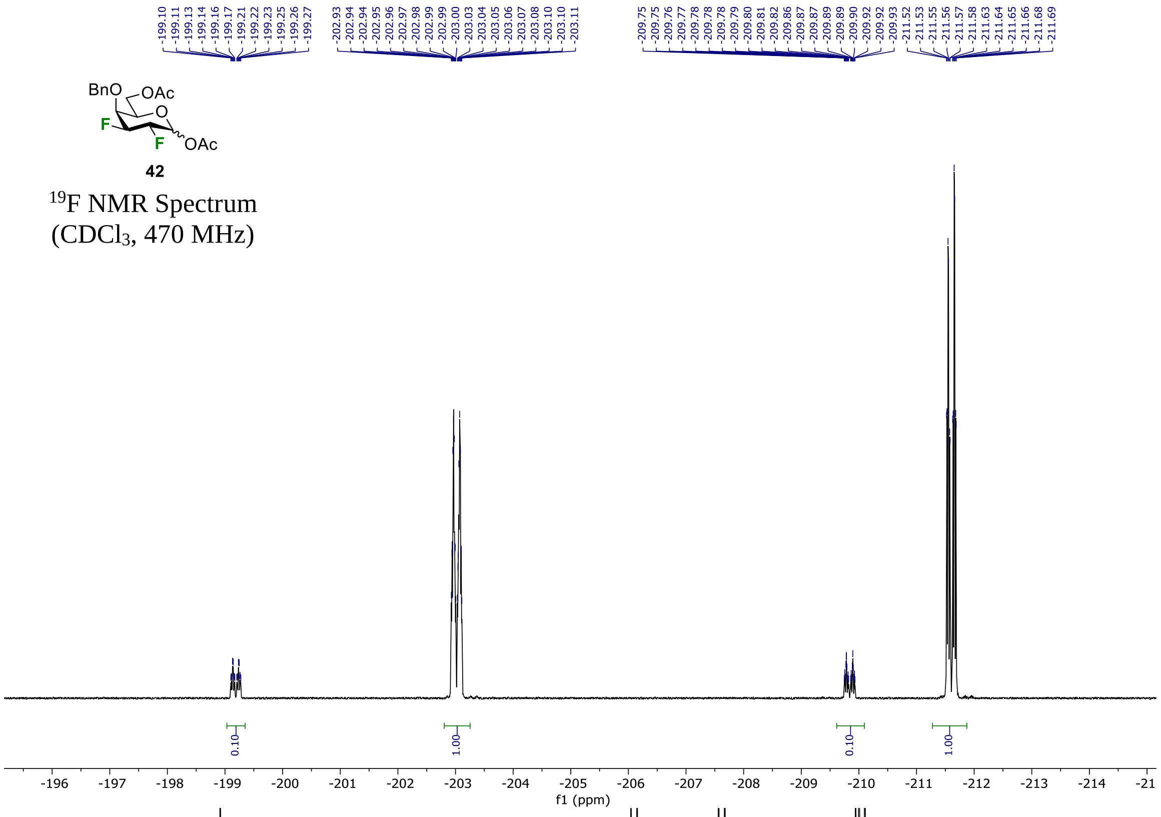
BnO
OAc
F
F
O
OAc
42
19F NMR Spectrum
(CDCl3, 470 MHz)
0.10
1.00
0.10
1.00
f1 (ppm)

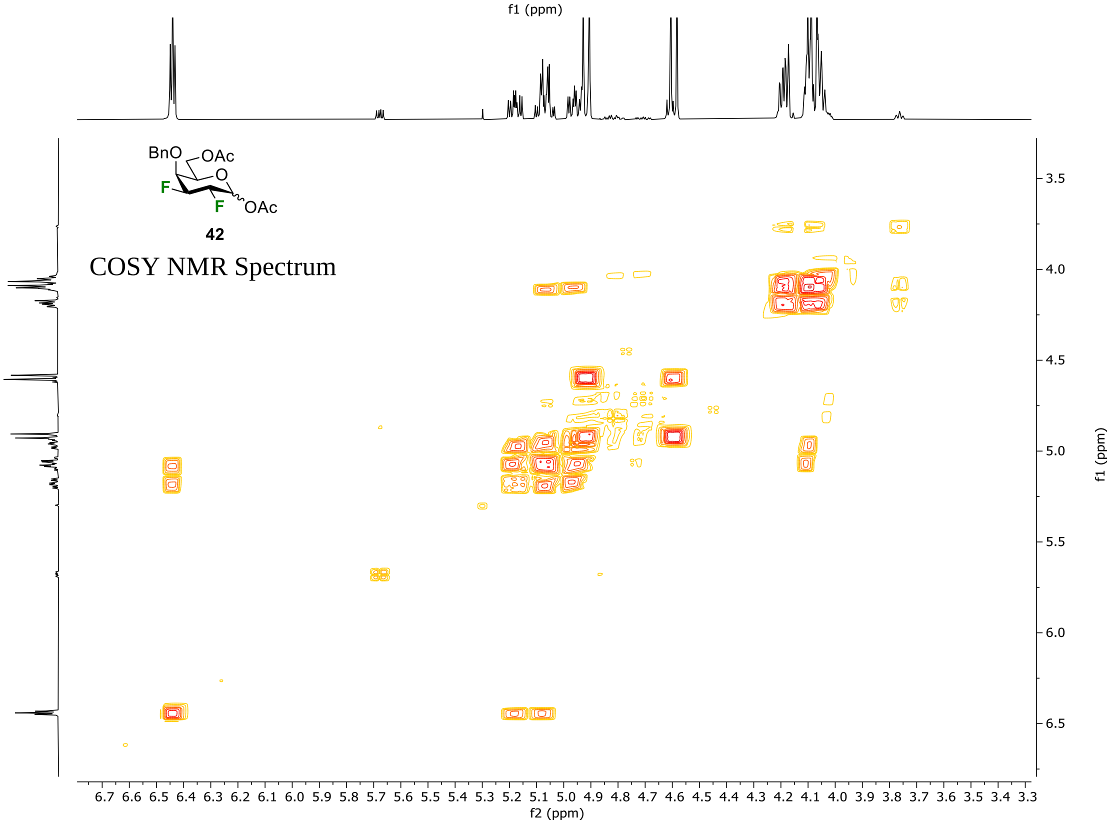
BnO
OAc
F
F
O
OAc
42
COSY NMR Spectrum
f2 (ppm)
f1 (ppm)

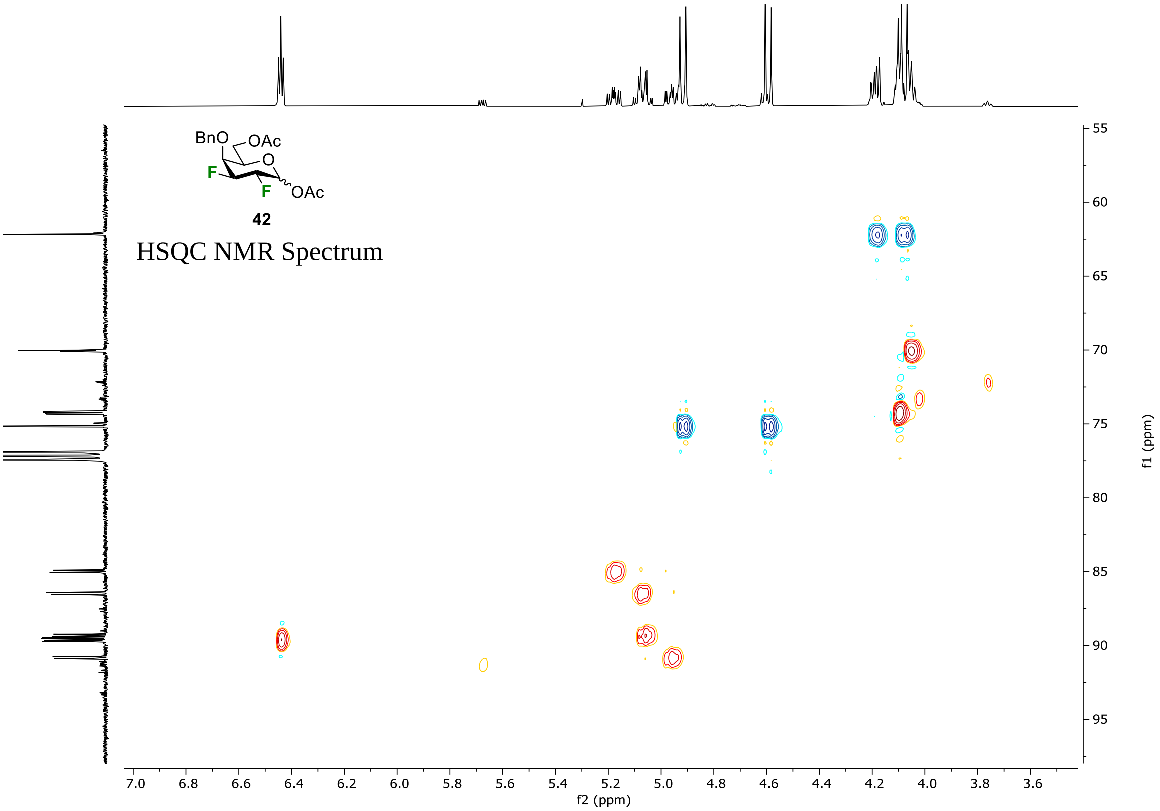
BnO
OAc
O
F
F
OAc
42
HSQC NMR Spectrum
f2 (ppm)
f1 (ppm)

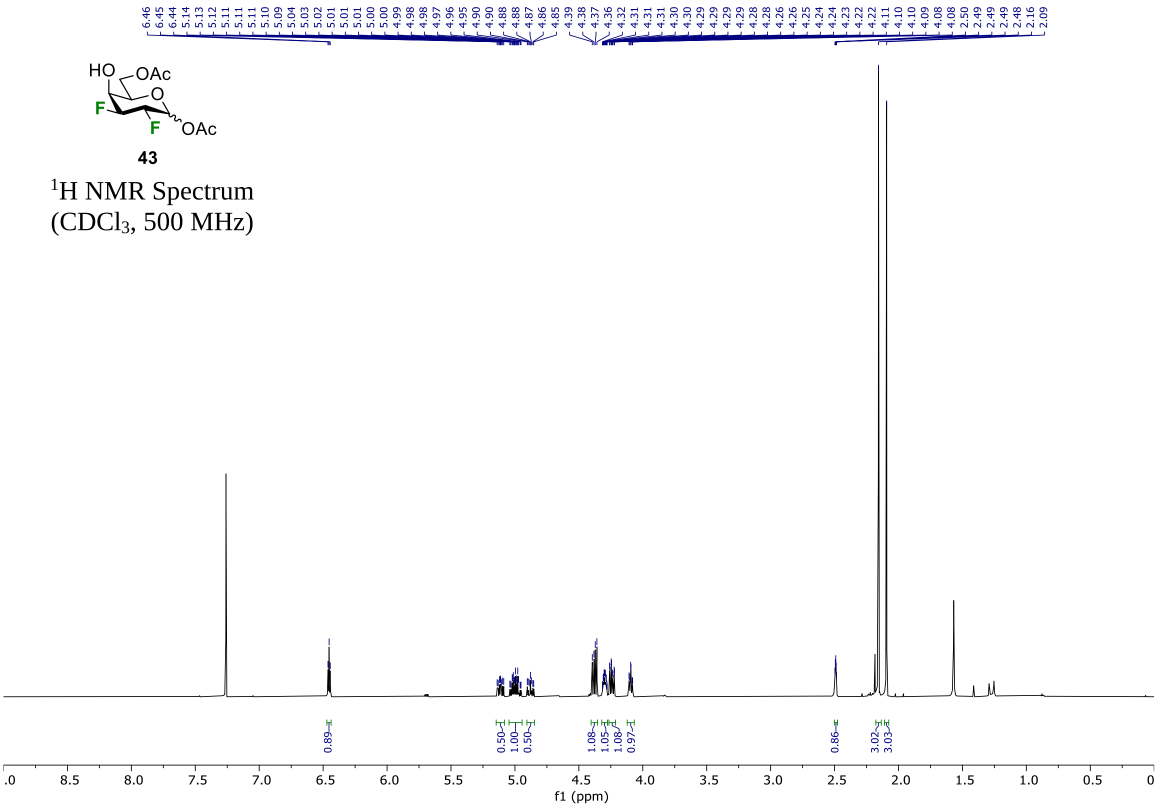
6.46 6.45 6.44 5.14 5.13 5.12 5.11 5.11 5.11 5.10 5.09 5.04 5.03 5.02 5.01 5.01 5.01 5.00 4.99 4.98 4.98 4.97 4.96 4.95 4.90 4.88 4.88 4.87 4.86 4.85 4.39 4.38 4.37 4.36 4.32 4.31 4.31 4.31 4.30 4.29 4.29 4.29 4.29 4.28 4.28 4.26 4.26 4.25 4.24 4.24 4.23 4.22 4.22 4.11 4.10 4.10 4.09 4.08 4.08 2.50 2.49 2.49 2.49 2.48 2.16 2.09
HO
OAc
O
F
F
OAc
43
¹H NMR Spectrum
(CDCl3, 500 MHz)
0.89 0.50 1.00 0.50 1.08 1.05 1.08 0.97 0.86 3.02 3.03
f1 (ppm)

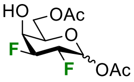

43

^{13}C NMR Spectrum
($CDCl_3$, 126 MHz)

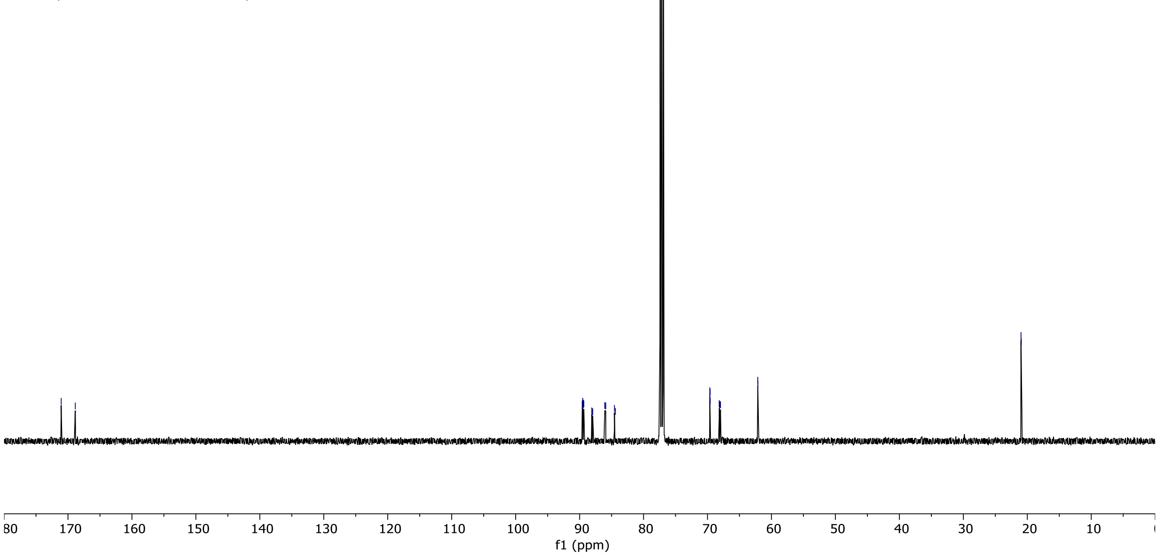

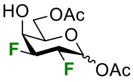

43

^{19}F NMR Spectrum
($CDCl_3$, 470 MHz)

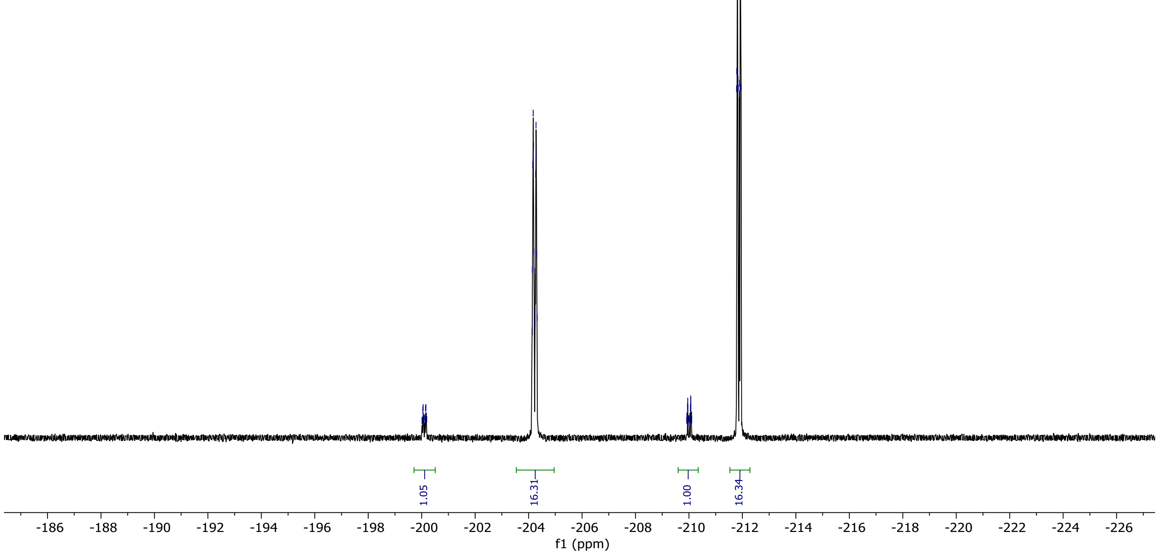

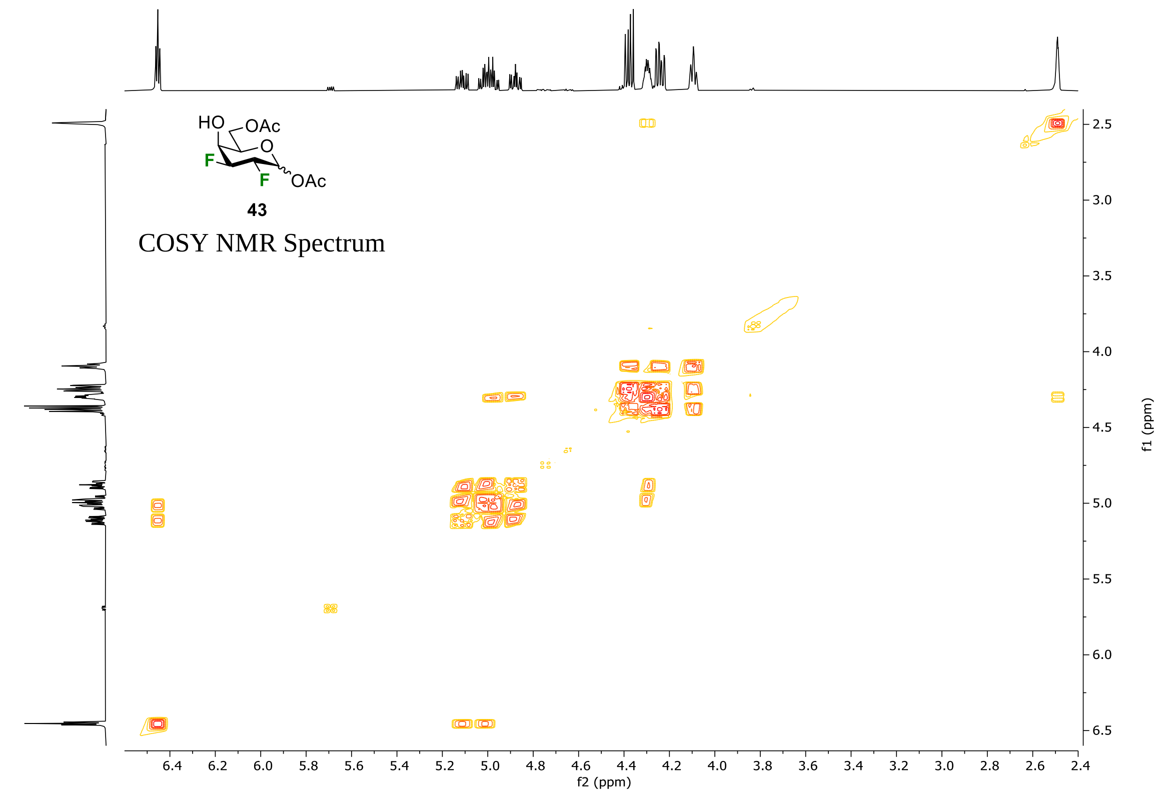
HO
OAc
O
F
F
OAc
43
COSY NMR Spectrum
f2 (ppm)
f1 (ppm)

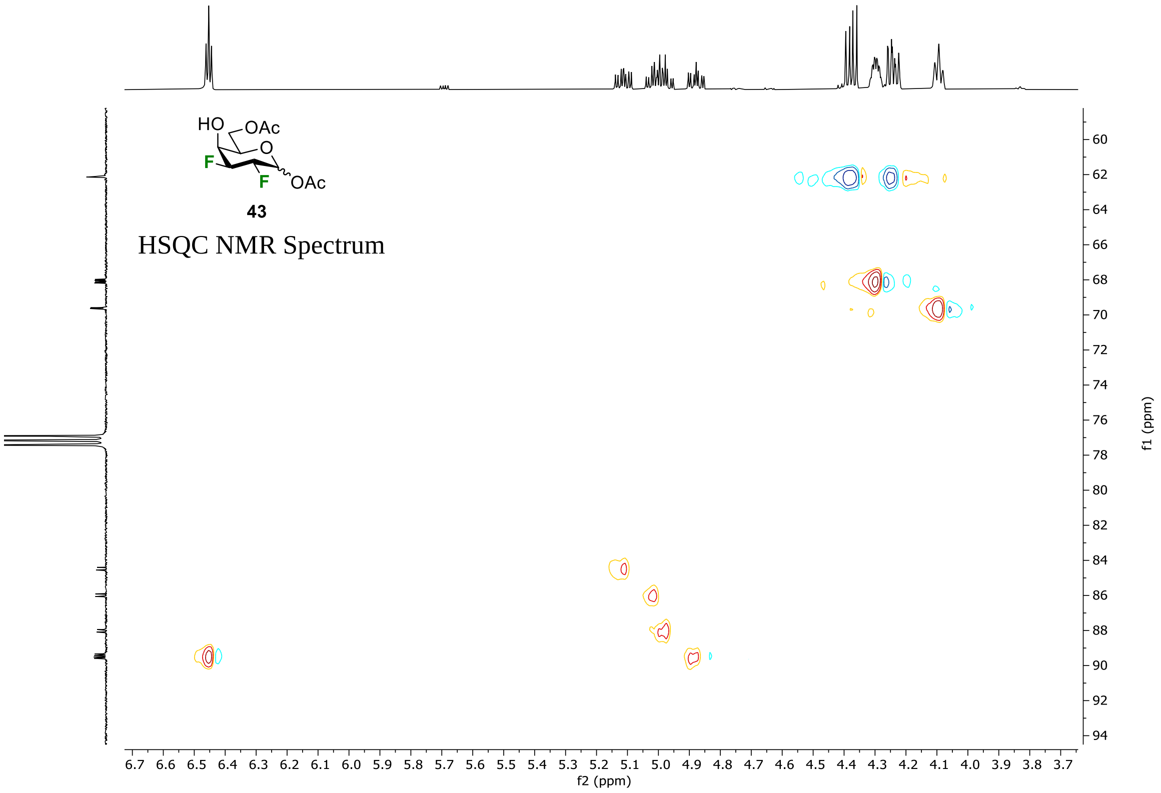
HO
OAc
O
F
F
OAc
43
HSQC NMR Spectrum
f2 (ppm)
f1 (ppm)

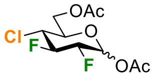

45

^{1}H NMR Spectrum
($CDCl_3$, 500 MHz)

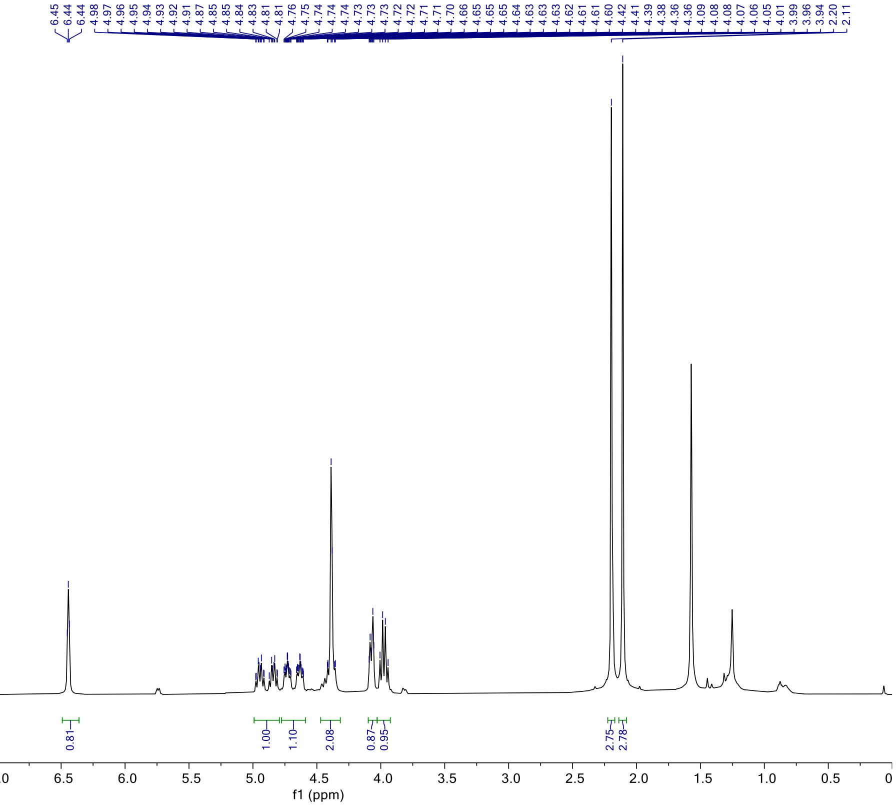

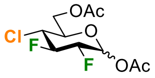

45

^{13}C NMR Spectrum
($CDCl_3$, 126 MHz)

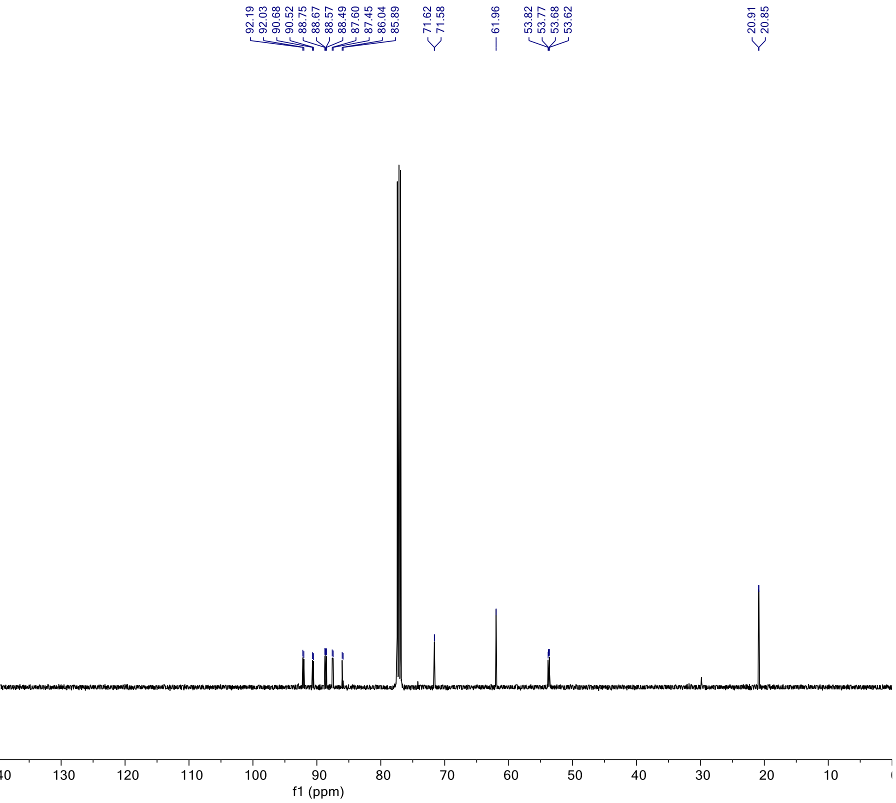

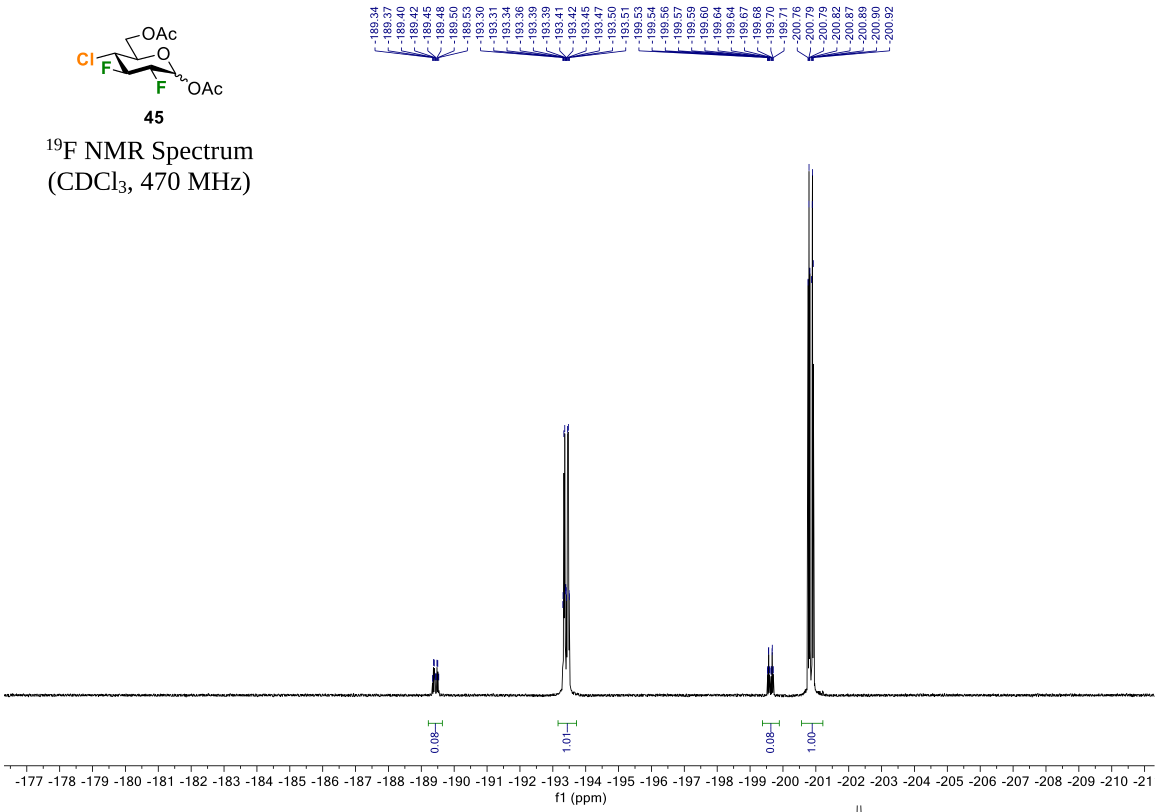

45

^{19}F NMR Spectrum
($CDCl_3$, 470 MHz)

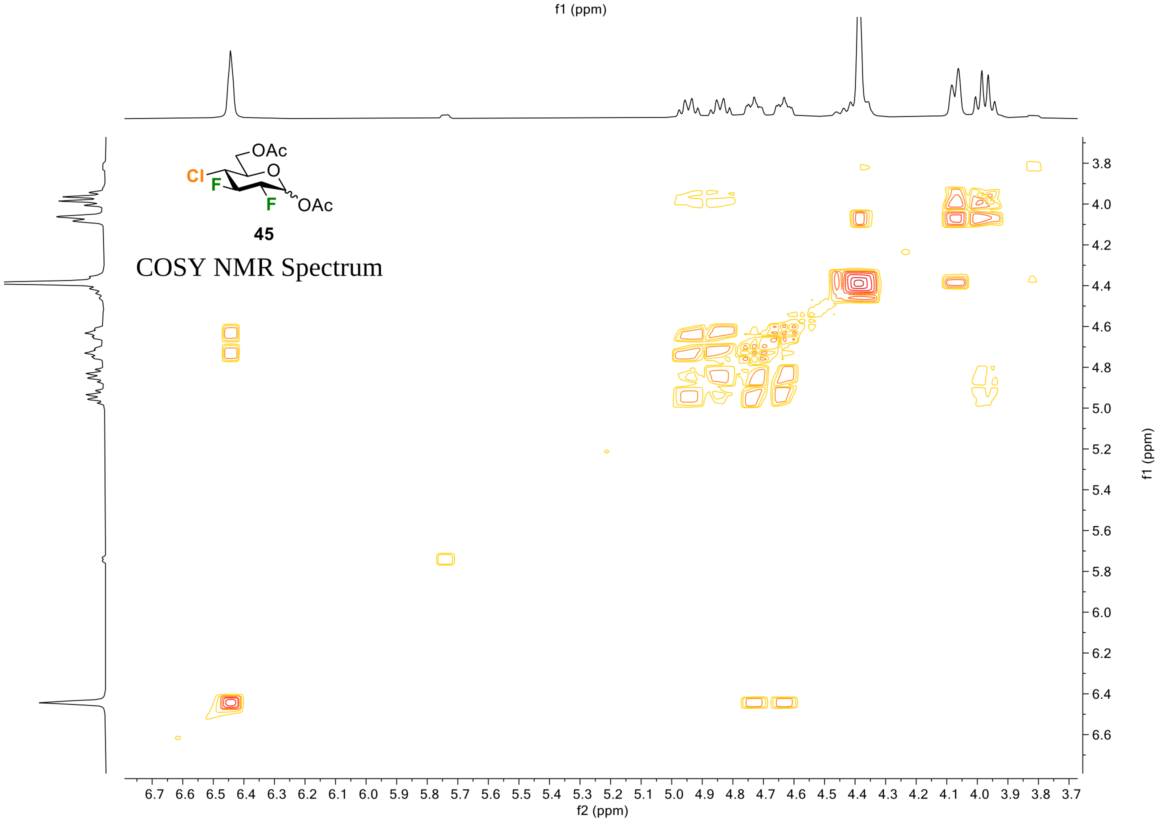

45

COSY NMR Spectrum

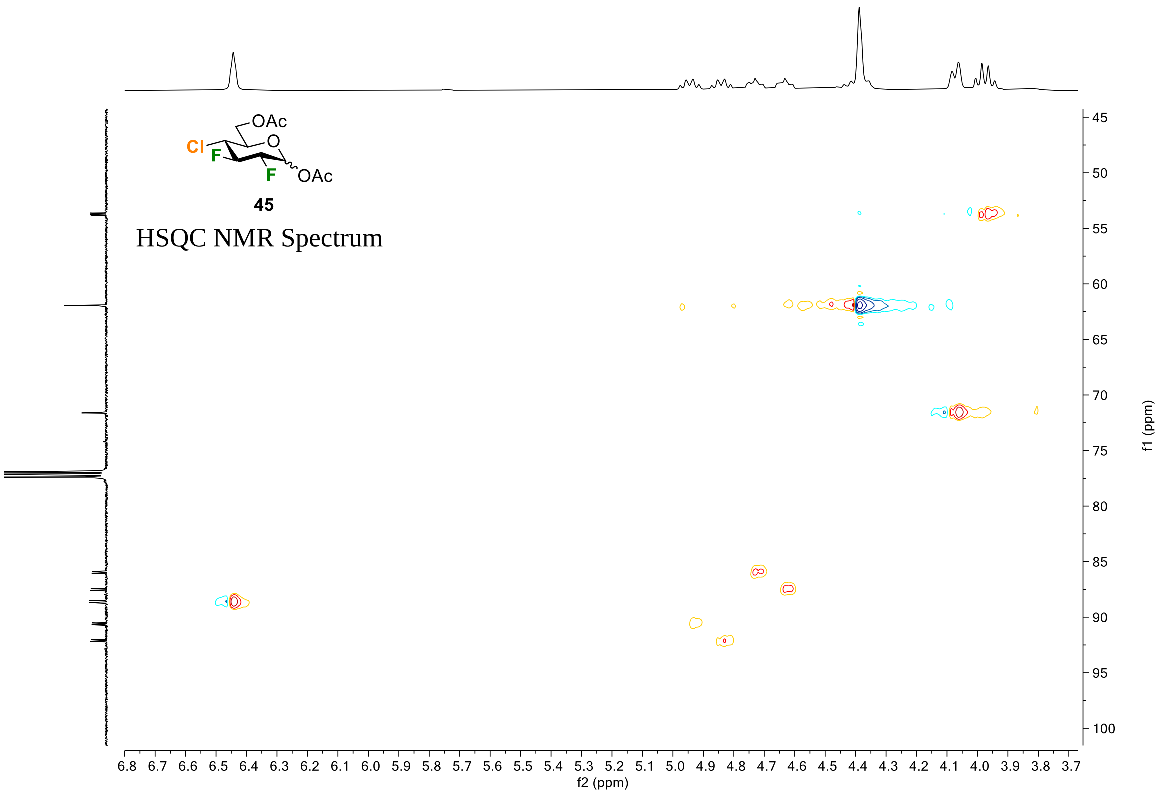
OAc
Cl
F
O
F
OAc
45
HSQC NMR Spectrum
f1 (ppm)
f2 (ppm)

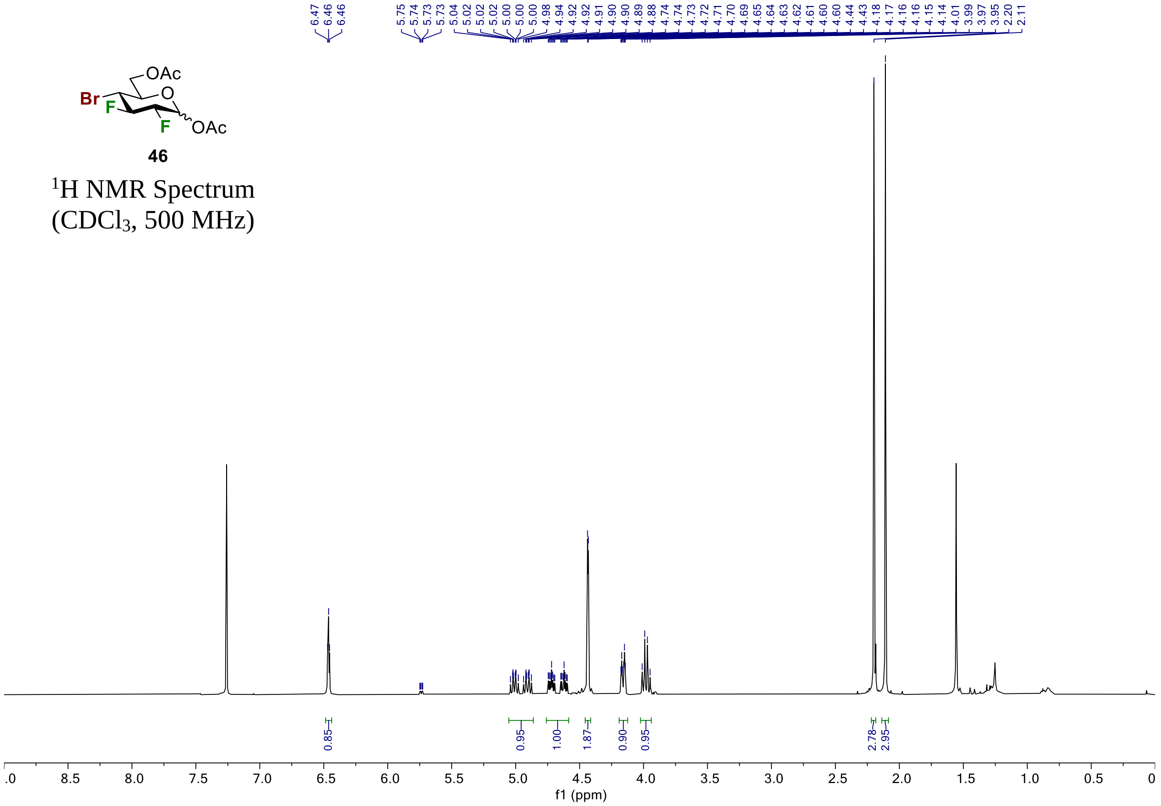
OAc
Br
F
O
F
OAc
46
¹H NMR Spectrum
(CDCl₃, 500 MHz)
f1 (ppm)

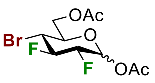

46

^{13}C NMR Spectrum
($CDCl_3$, 126 MHz)

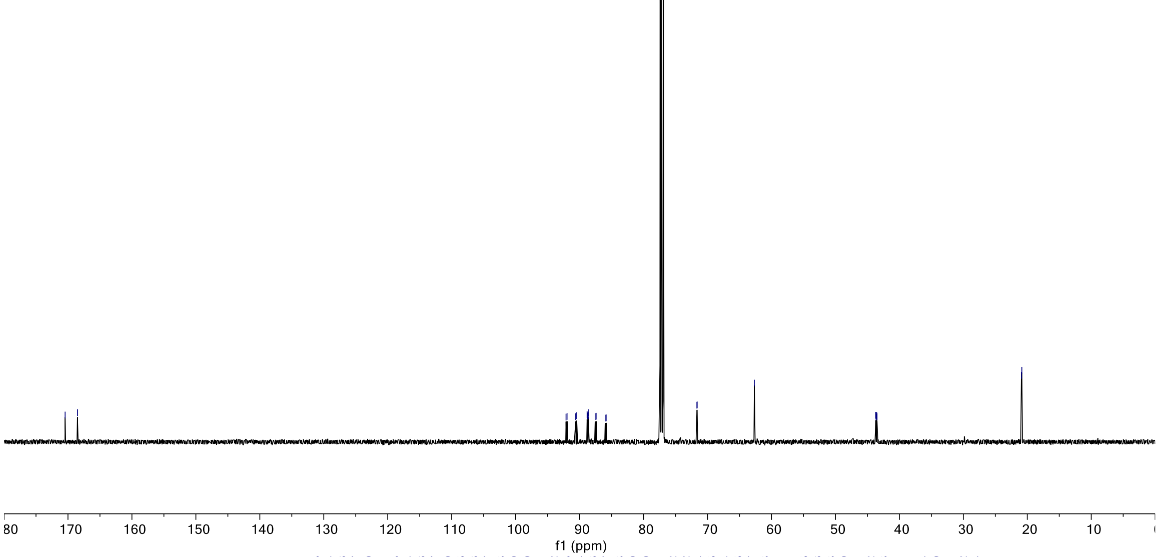

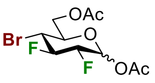

46

^{19}F NMR Spectrum
($CDCl_3$, 470 MHz)

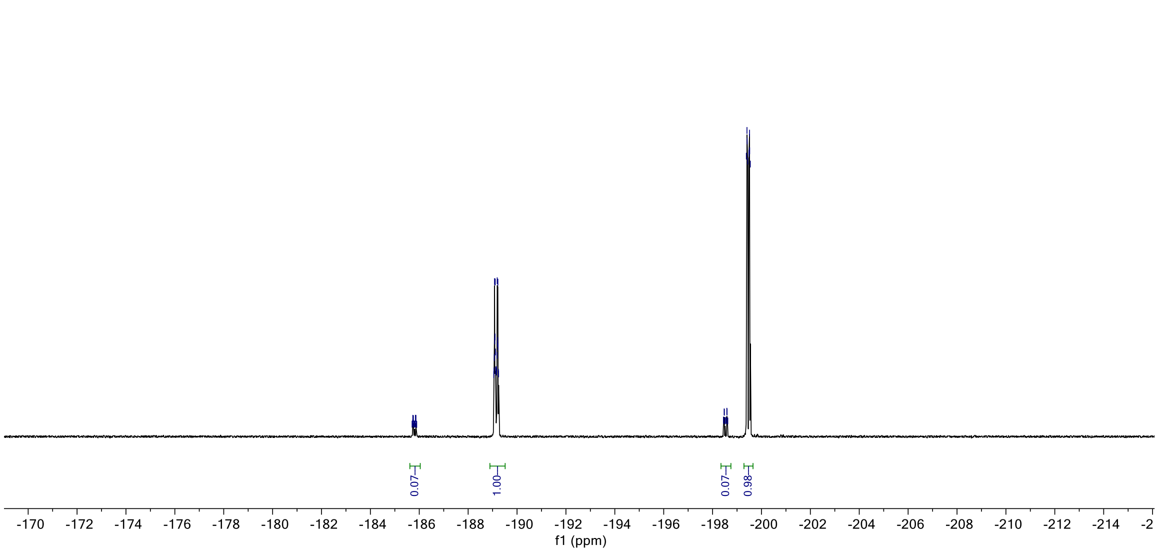

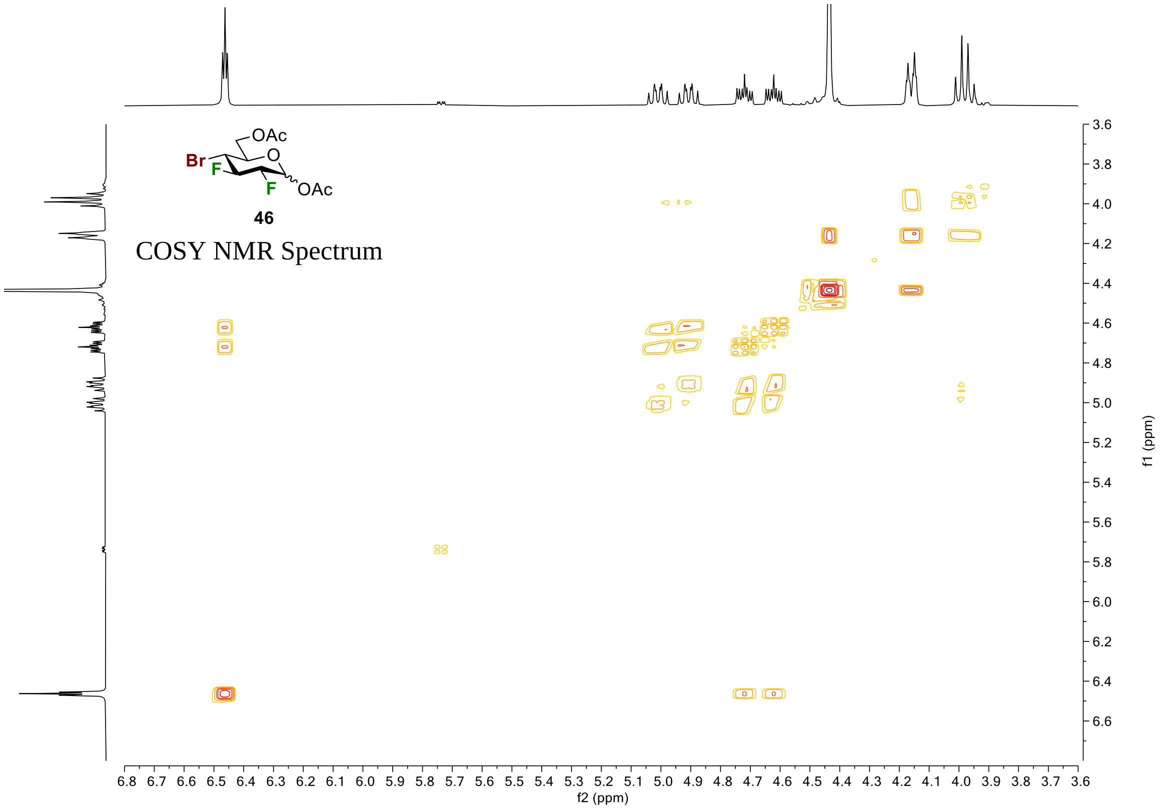
OAc
Br
F
O
F
OAc
46
COSY NMR Spectrum
f1 (ppm)
f2 (ppm)

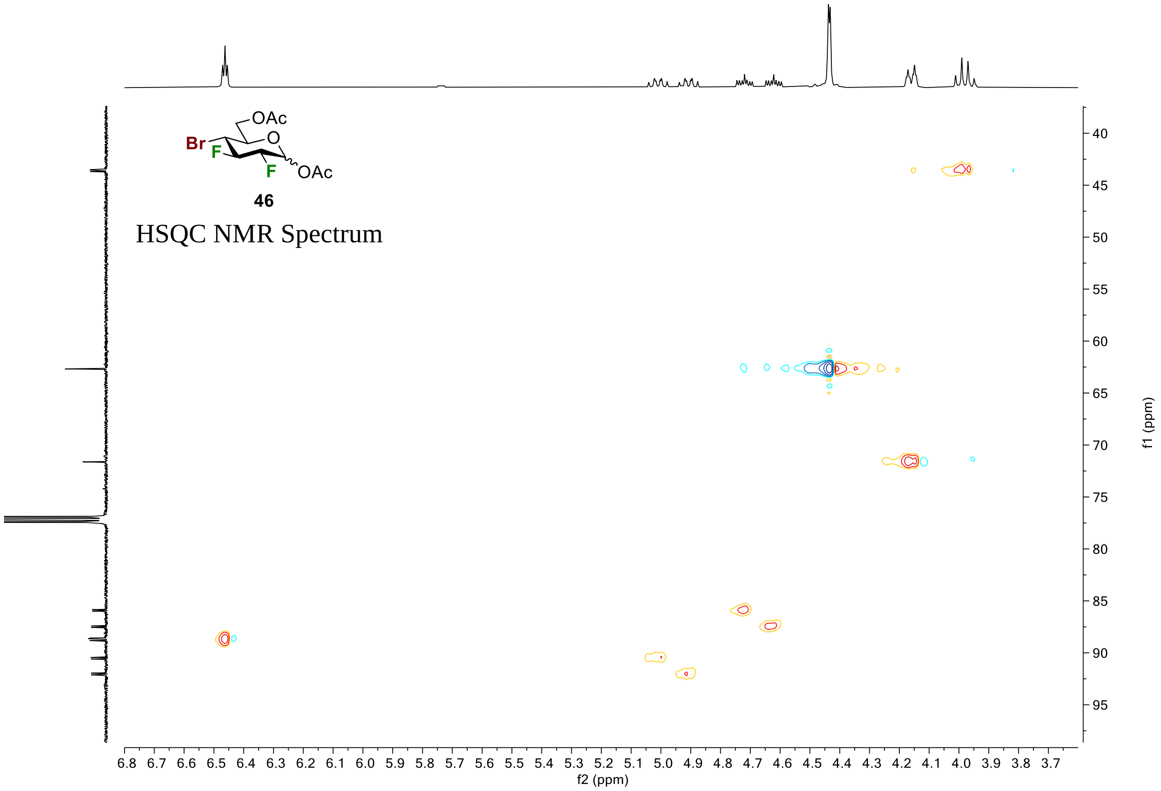
OAc
Br
F
O
F
OAc
46
HSQC NMR Spectrum
f1 (ppm)
f2 (ppm)

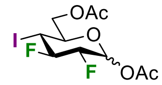

47

^{1}H NMR Spectrum
($CDCl_3$, 500 MHz)

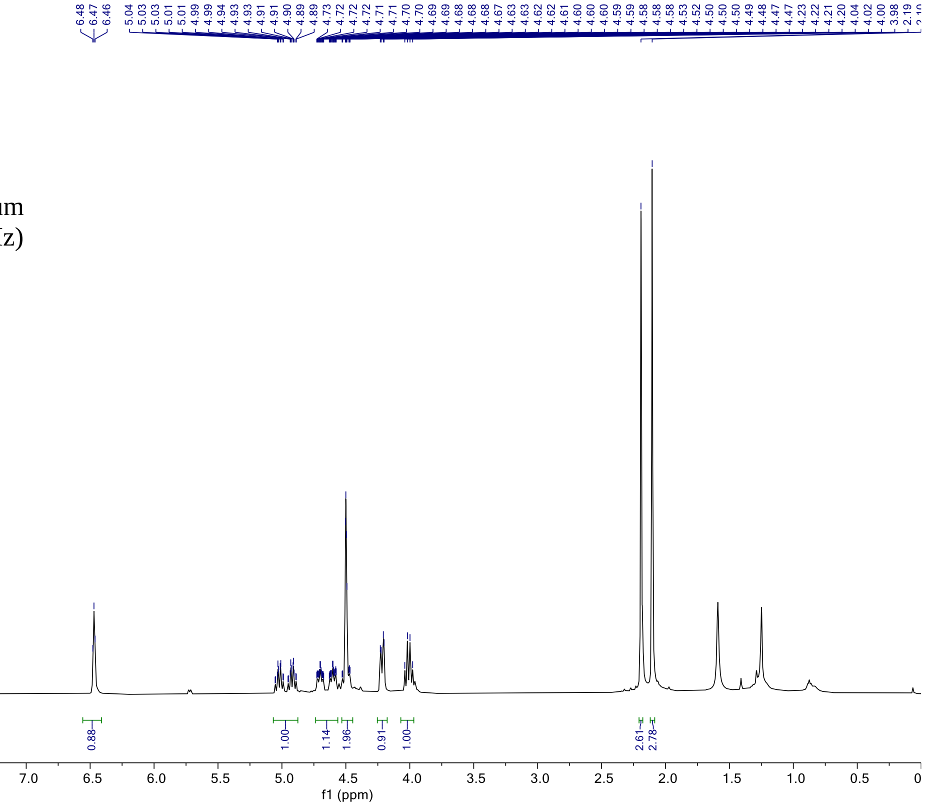

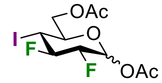

47

^{13}C NMR Spectrum
($CDCl_3$, 126 MHz)

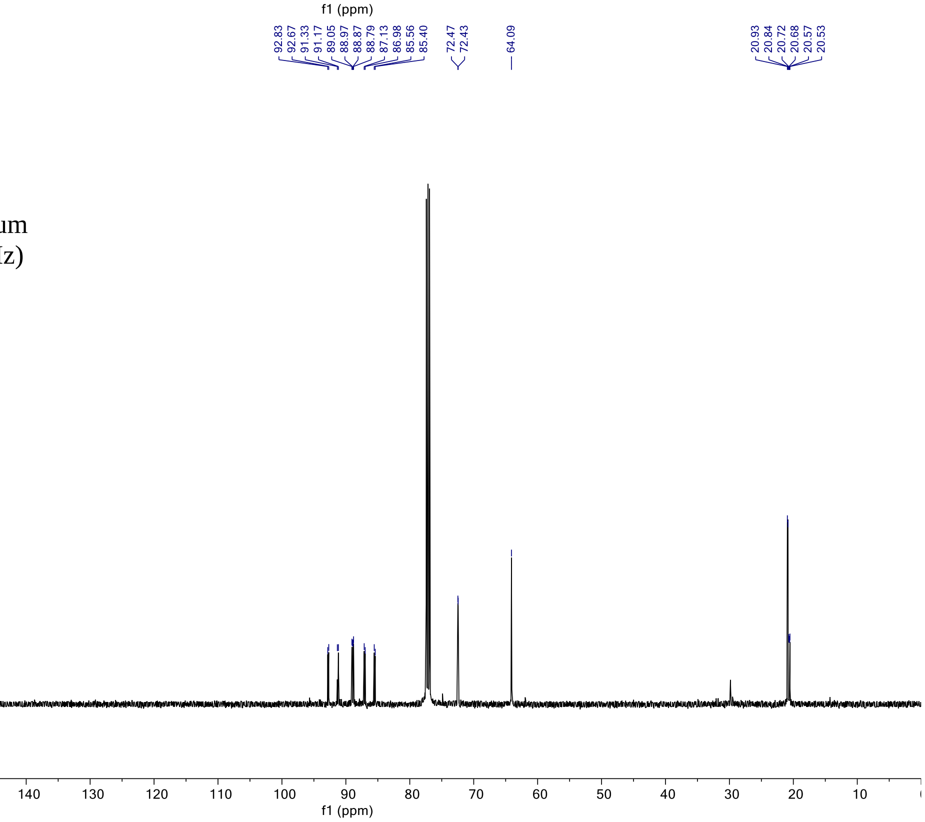

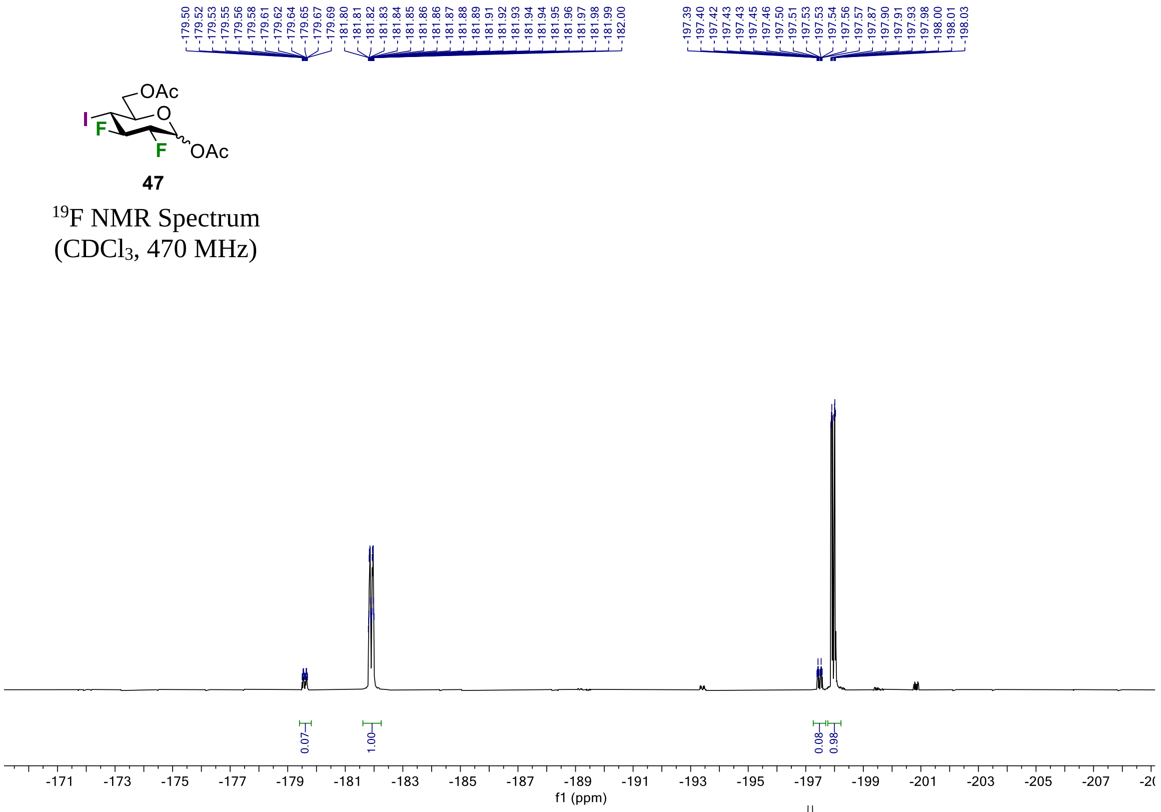

47

^{19}F NMR Spectrum
($CDCl_3$, 470 MHz)

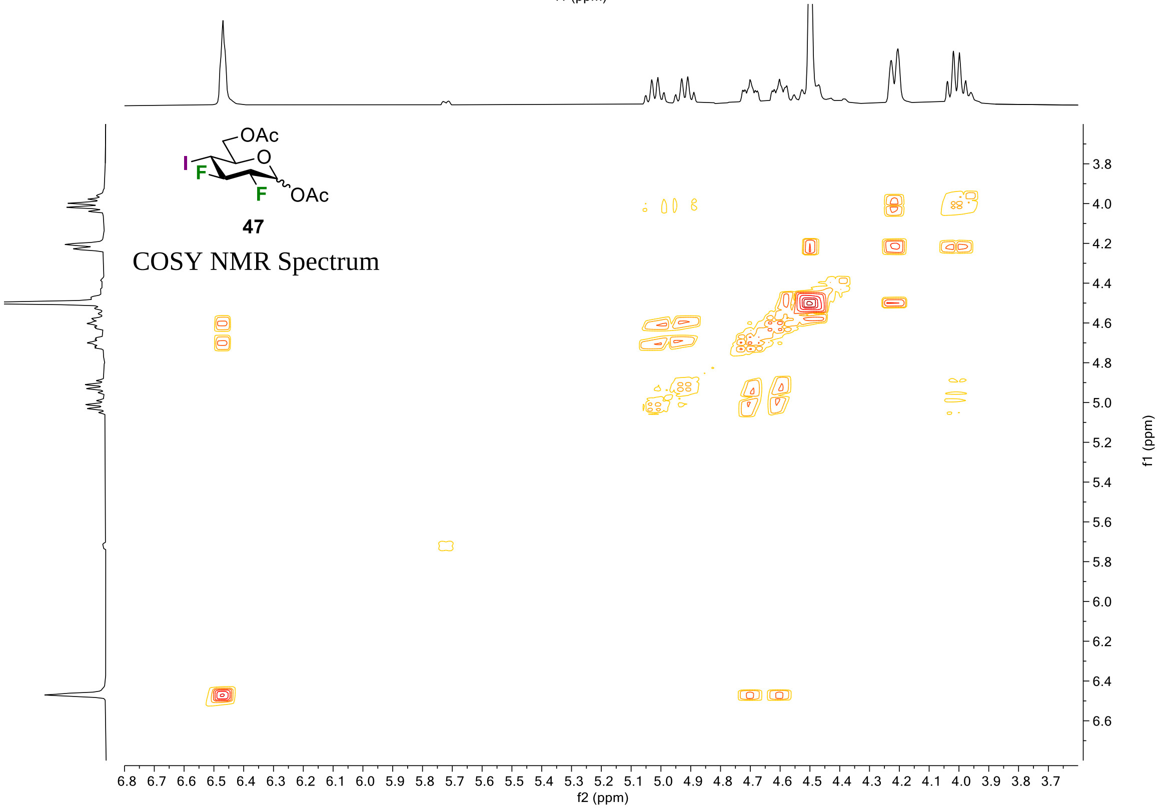

47

COSY NMR Spectrum

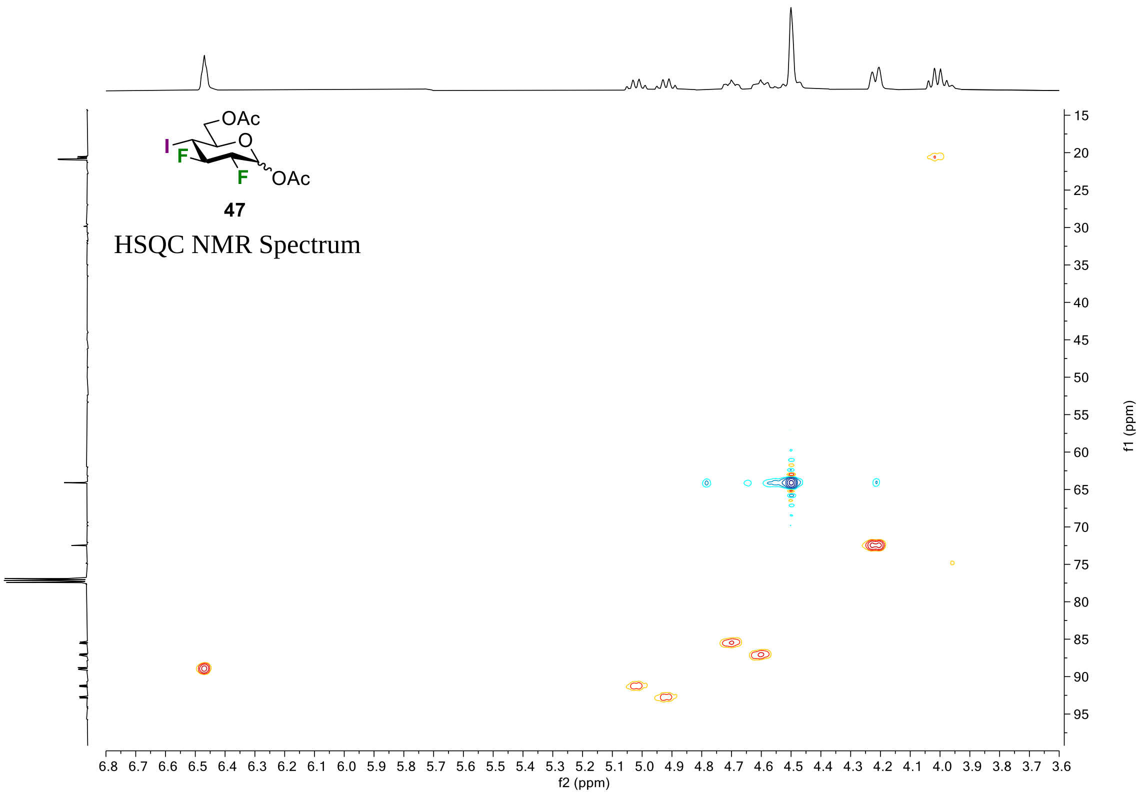

OAc
I
O
F
F
OAc
47
HSQC NMR Spectrum
f1 (ppm)
f2 (ppm)

V. References

[1] V. Denavit, D. Lainé, J. St-Gelais, P. A. Johnson, D. Giguère, *Nat. Commun.* **2018**, *9*, 4721.

[2] Y. Li, W.-S. Huang, L. Zhang, D. Su, H. Xu, X.-S. Xue, *Artificial Intelligence Chemistry* **2024**, *2*, 100043.

[3] NMRShiftDB. https://nmrshiftdb.nmr.uni-koeln.de/ (accessed 27 February 2025).
